# Supplementary material for: Aging and diet alter the protein ubiquitylation landscape in the mouse brain
Source: Nat Commun. 2025 Jun 6;16:5266. doi: 10.1038/s41467-025-60542-6 (PMC12144301; doi:10.1038/s41467-025-60542-6)
Supplement: Supplementary file 1 — Supplementary Information [file 41467_2025_60542_MOESM1_ESM.pdf]

# Aging and diet alter the protein ubiquitylation landscape in the mouse brain

Antonio Marino*, Domenico Di Fraia* *et al.*

Corresponding author: alessandro.ori@leibniz-fli.de

## Supplementary Information

**The PDF file includes:**

**Supplementary Figures S1 to S9**

**Supplementary Tables 1 and 2**

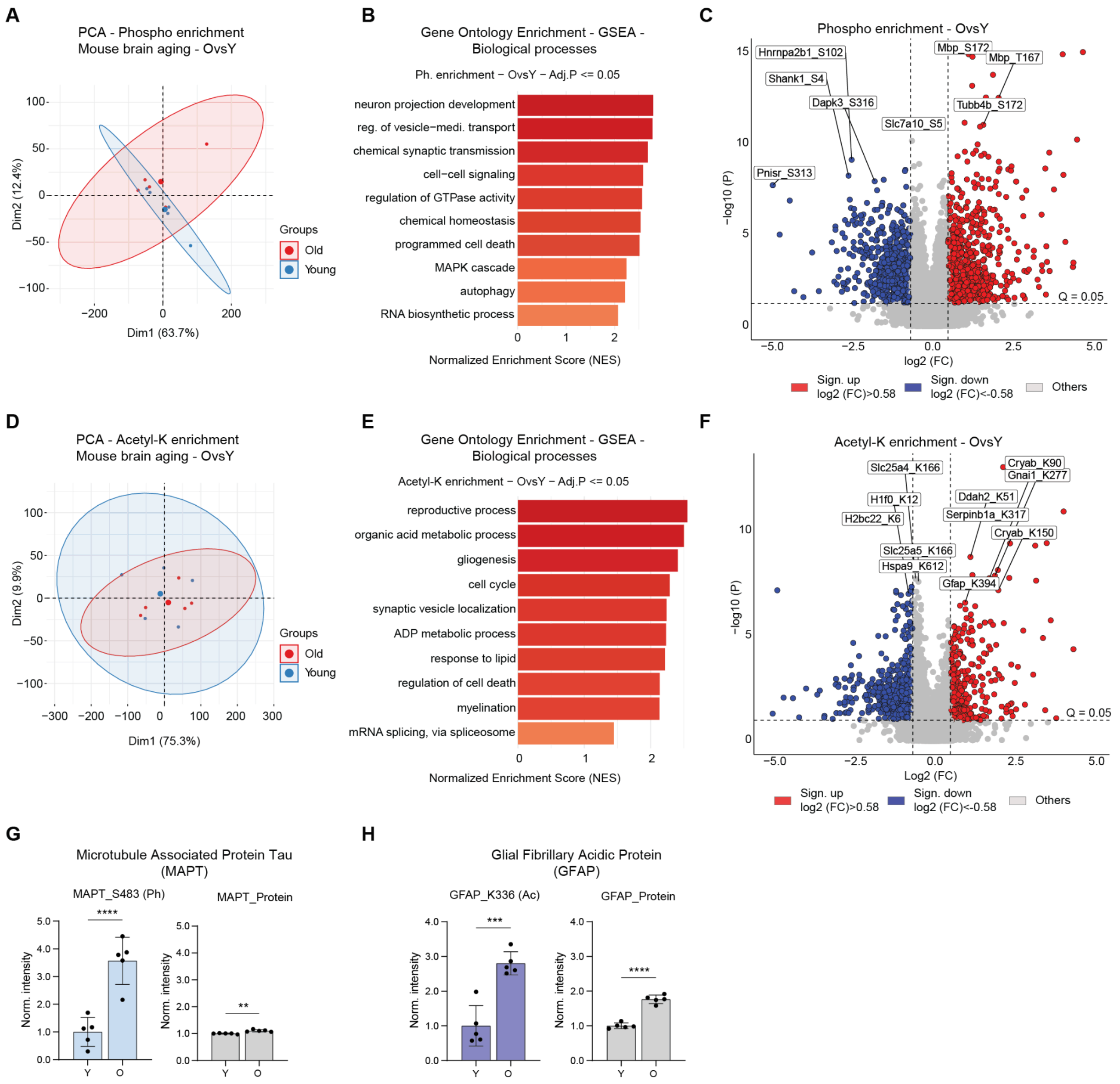


**Figure S1. Phosphorylation and acetylation characterization in the mouse aging brain**

A) PCA based on phosphorylated peptide abundances from mouse brains. Ellipses represent 95% confidence intervals. The percentage of variance explained by each principal component is indicated (N=5, biological replicates). B) Gene set enrichment analysis (GSEA) for phosphorylated peptides affected by aging based on GO biological process terms (Adj.P<0.05, weighted Kolmogorov–Smirnov test). C) Volcano plot for phospho-enrichment in mouse brain aging (N=5). D) PCA based on acetylated peptide abundances from mouse brains. Ellipses represent 95% confidence intervals. The percentage of variance explained by each principal component is indicated (N=5, biological replicates). E) Gene set enrichment analysis (GSEA) for acetylated peptides affected by aging based on GO biological process terms (Adj.P<0.05, weighted Kolmogorov–Smirnov test). F) Volcano plot for acetyl-enrichment in mouse brain aging (N=5, biological replicates). G) Changes of MAPT phosphorylation and protein levels during aging (N=5, biological replicates, Q values from Spectronaut differential abundance analysis, data shown as averages ± SD). H) Changes of GFAP

acetylation and protein levels during aging (N=5, biological replicates, Q values from Spectronaut differential abundance analysis, data shown as averages ± SD).*Q / Adj.P ≤ 0.05; **Q / Adj.P ≤ 0.01, ***Q/ Adj.P ≤ 0.001, ****Q / Adj.P ≤ 0.0001.

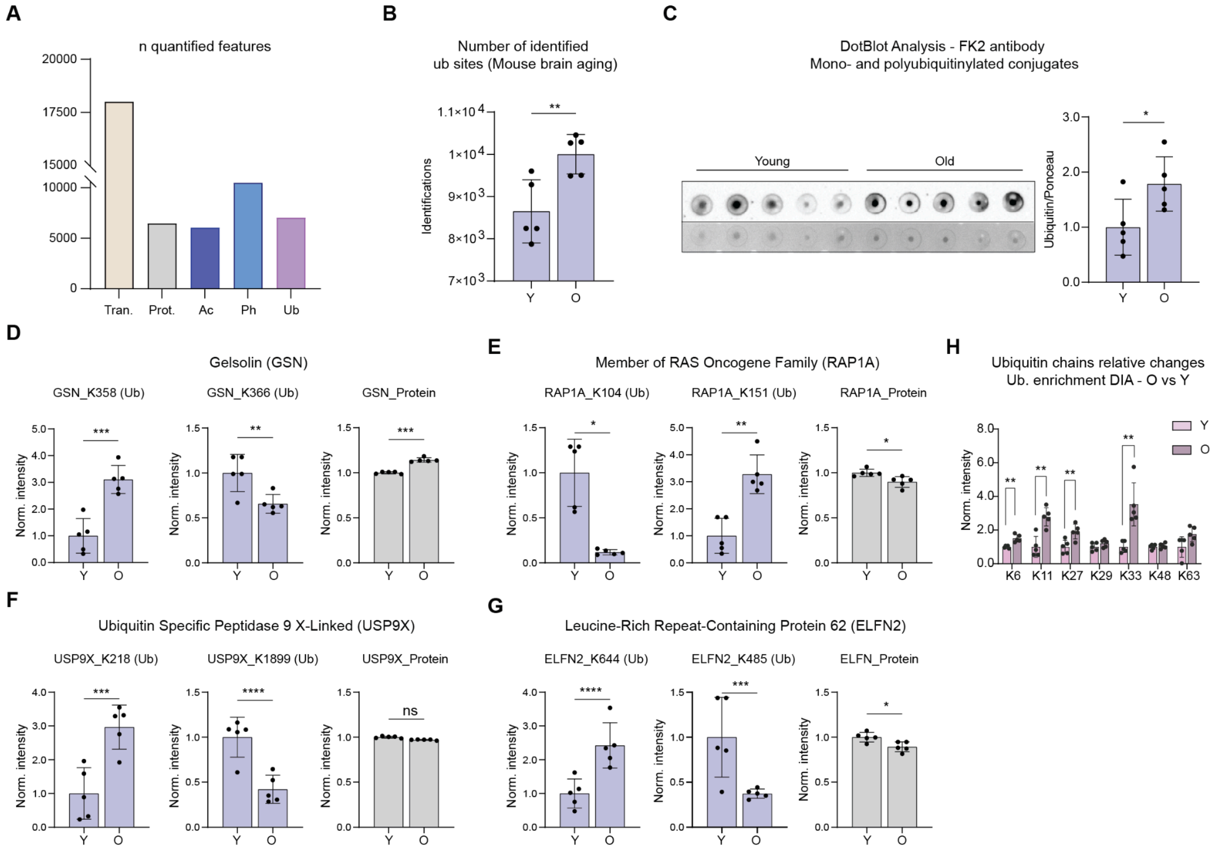


**Figure S2. Changes in ubiquitylation in mouse brain aging**

A) Total number of quantified features in each dataset. B) Barplots showing the numbers of identified ubiquitylated sites in the different age groups (N=5, biological replicates, unpaired *t*-test). C) Dot blot for total ubiquitin conjugates and free ubiquitin in young and old mouse brain lysates (N=5, biological replicates, unpaired *t*-test). D-G) Examples of proteins showing age-related changes of ubiquitylation (N=5, biological replicates, Q values from Spectronaut differential abundance analysis, data shown as averages ± SD). H) Ubiquitin chains intensity in old and young mice brains measured by DIA mass spectrometry following K-ε-GG enrichment (N=5, biological replicates, unpaired *t*-test, data shown as averages ± SD). Source data are provided as a Source Data file. Specific p values are listed in Supplementary Data 8. *Q / Adj.P ≤ 0.05; **Q / Adj.P ≤ 0.01, ***Q/ Adj.P ≤ 0.001, ****Q / Adj.P ≤ 0.0001.

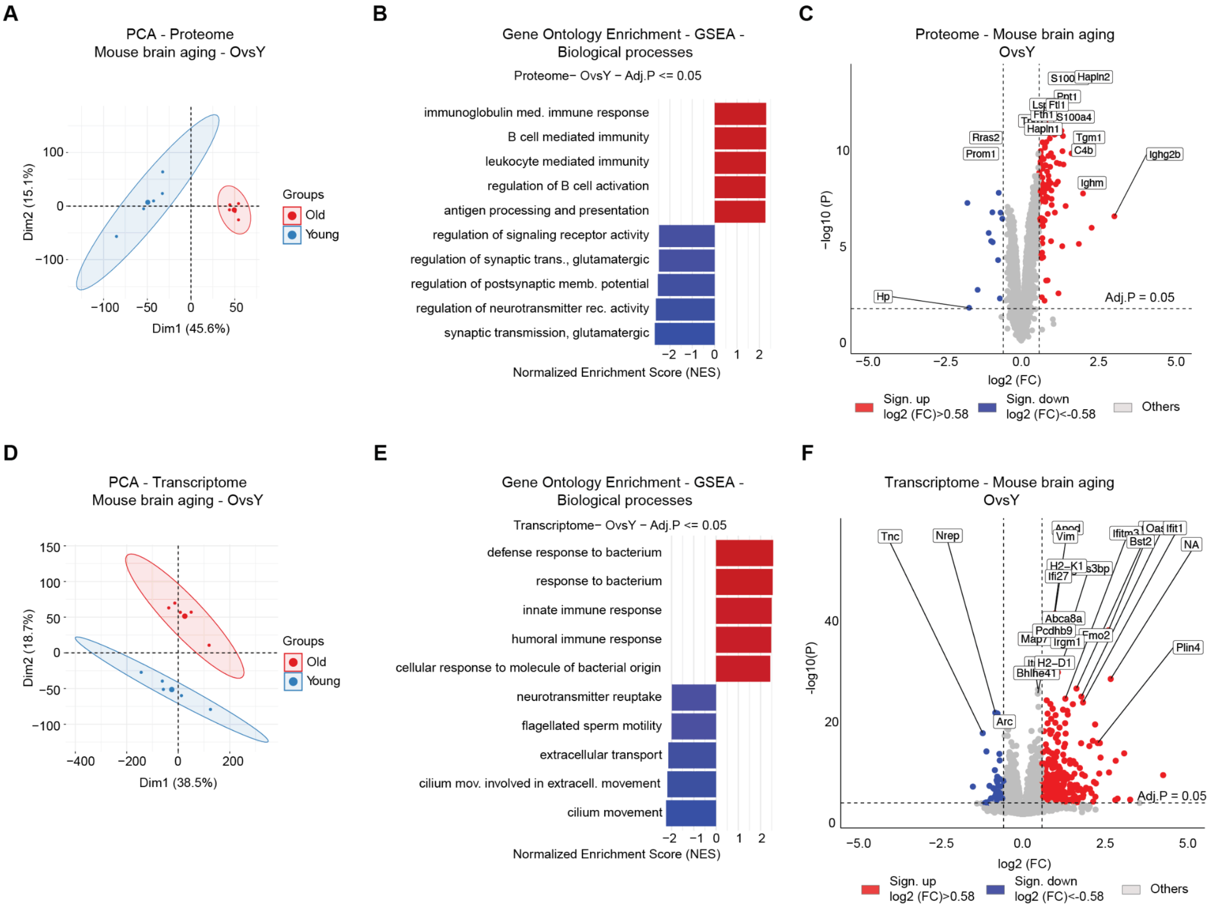


**Figure S3. Changes in whole proteome and transcriptome in mouse brain aging**

A) PCA based on total proteome data. Ellipses represent 95% confidence intervals. The percentage of variance explained by each principal component is indicated (N=5, biological replicates). B) Gene set enrichment analysis (GSEA) for proteins affected by aging based on GO biological process terms (Adj.P<0.05, weighted Kolmogorov–Smirnov test). C) Volcano plot for proteome in mouse brain aging (N=5, biological replicates). D) PCA based on transcriptome data. Ellipses represent 95% confidence intervals. The percentage of variance explained by each principal component is indicated (N=5, biological replicates). E) Gene set enrichment analysis (GSEA) for transcripts affected by aging based on GO biological process terms (Adj.P<0.05, weighted Kolmogorov–Smirnov test). F) Volcano plot for transcriptome in mouse brain aging (N=5, biological replicates).

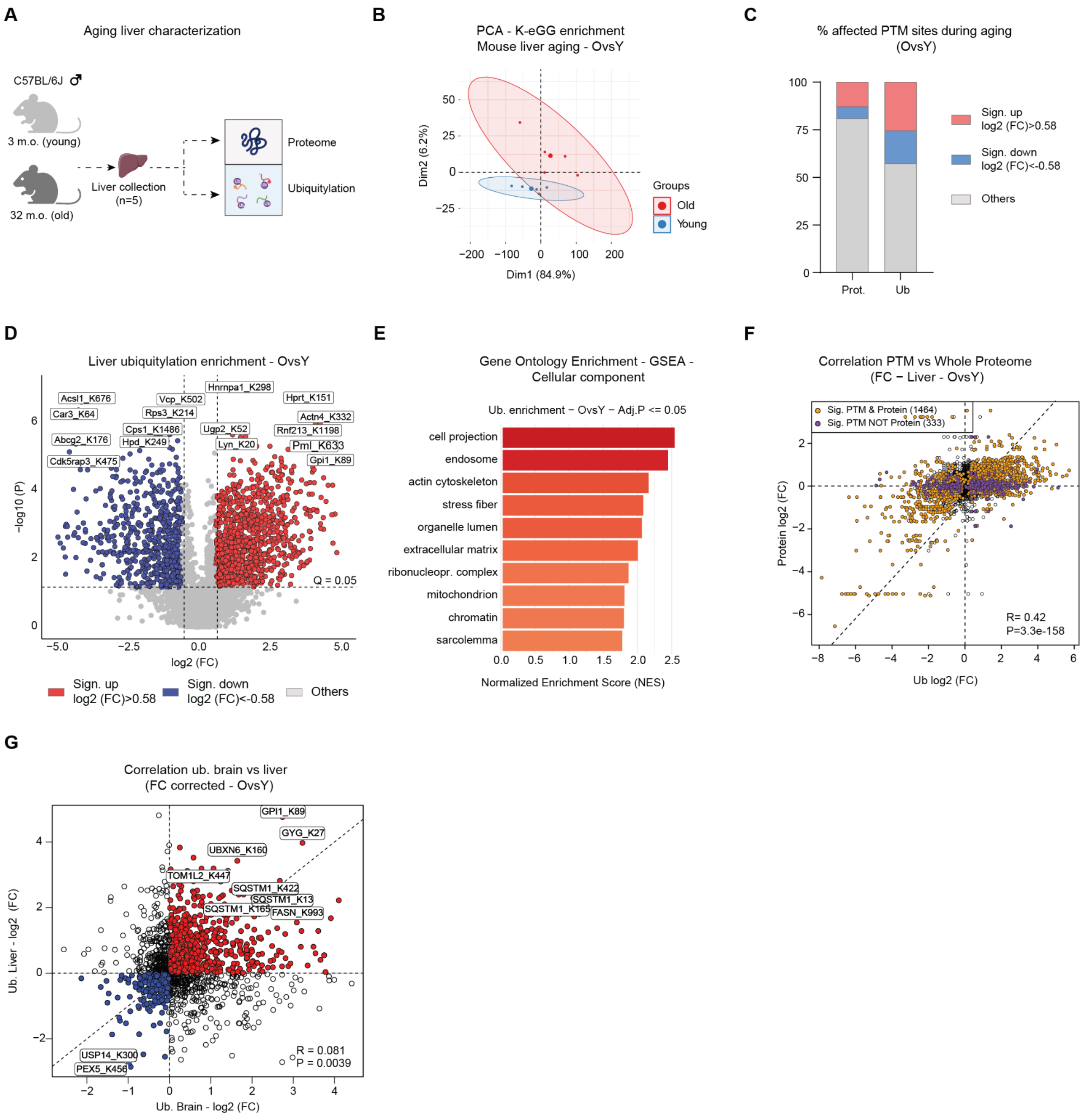


**Figure S4. Changes in ubiquitylation in mouse liver aging**

A) Scheme of the approach used to characterize changes of protein ubiquitylation in mouse liver aging (3 vs. 32 months old, N=5, biological replicates, males, C57BL/6J, one replicate in the proteome dataset was omitted following quality control assessment). Created in BioRender. Ori, A. (2025) https://BioRender.com/2947h3v. B) PCA plot based on ubiquitylated peptides' abundances. Ellipses represent 95% confidence intervals. The percentage of variance explained by each principal component is indicated (N=5, biological replicates). C) Percentage of significantly affected proteins, or ubiquitylated peptides (Q<0.05 from Spectronaut differential abundance analysis and absolute log2 (FC)>0.58). D) Volcano plot for ubiquitin enrichment in mouse liver aging (N=5, biological replicates). E) Gene set enrichment analysis (GSEA) for ubiquitylated peptides affected by aging based on GO cellular component terms (Adj.P<0.05, weighted Kolmogorov–Smirnov test). F) Scatterplot illustrating the relationship between fold changes in protein abundance and corresponding ubiquitylated peptide levels with age. Proteins showing significant

age-related changes at both the total protein and ubiquitylated peptide level (Q<0.05) are highlighted in orange. Proteins with significant changes in ubiquitylation only (protein Q≥0.05 and ubiquitylated peptide Q<0.01) are shown in purple. Q values from Spectronaut differential abundance analysis. Two-sided Pearson's correlation test. G) Scatterplot showing the correlation between ubiquitylated peptide abundance changes with age in the brain (x-axis) and liver (y-axis). Ubiquitylated peptide levels were corrected for protein abundance changes. Two-sided Pearson's correlation test.

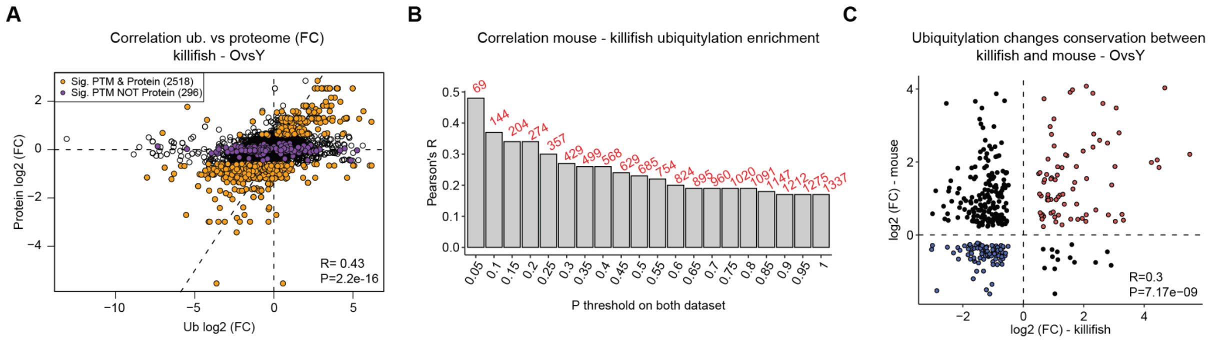


**Figure S5. Age-related ubiquitylation changes comparison between killifish and mouse**

A) Scatterplot illustrating the relationship between fold changes in protein abundance and corresponding ubiquitylated peptide levels with age. Proteins showing significant age-related changes at both the total protein (Q<0.05 and absolute log2 (FC)>0.58) and ubiquitylated peptide level (Q<0.05) are highlighted in orange. Proteins with significant changes in ubiquitylation only (protein Q≥0.05 and ubiquitylated peptide Q<0.01) are shown in purple (N=4 for young and N=3 for old killifish, biological replicates). Q values from Spectronaut differential abundance analysis. Two-sided Pearson's correlation test. B) Barplot illustrating the correlation between ubiquitylated peptide abundance changes in mouse and killifish based on different P value thresholds. The red numbers indicate the number of conserved ubiquitylation sites that pass the indicated P value threshold. C) Scatterplot showing the relationship between ubiquitylated peptide abundance changes with age in killifish (x-axis) and mouse (y-axis) brain. Conserved sites that show increased or decreased ubiquitylation with age in both species (P<0.25, limma's empirical Bayes moderated t-test) are highlighted in red and blue, respectively. Ubiquitylated peptide levels were corrected for protein abundance changes. Two-sided Pearson's correlation test.

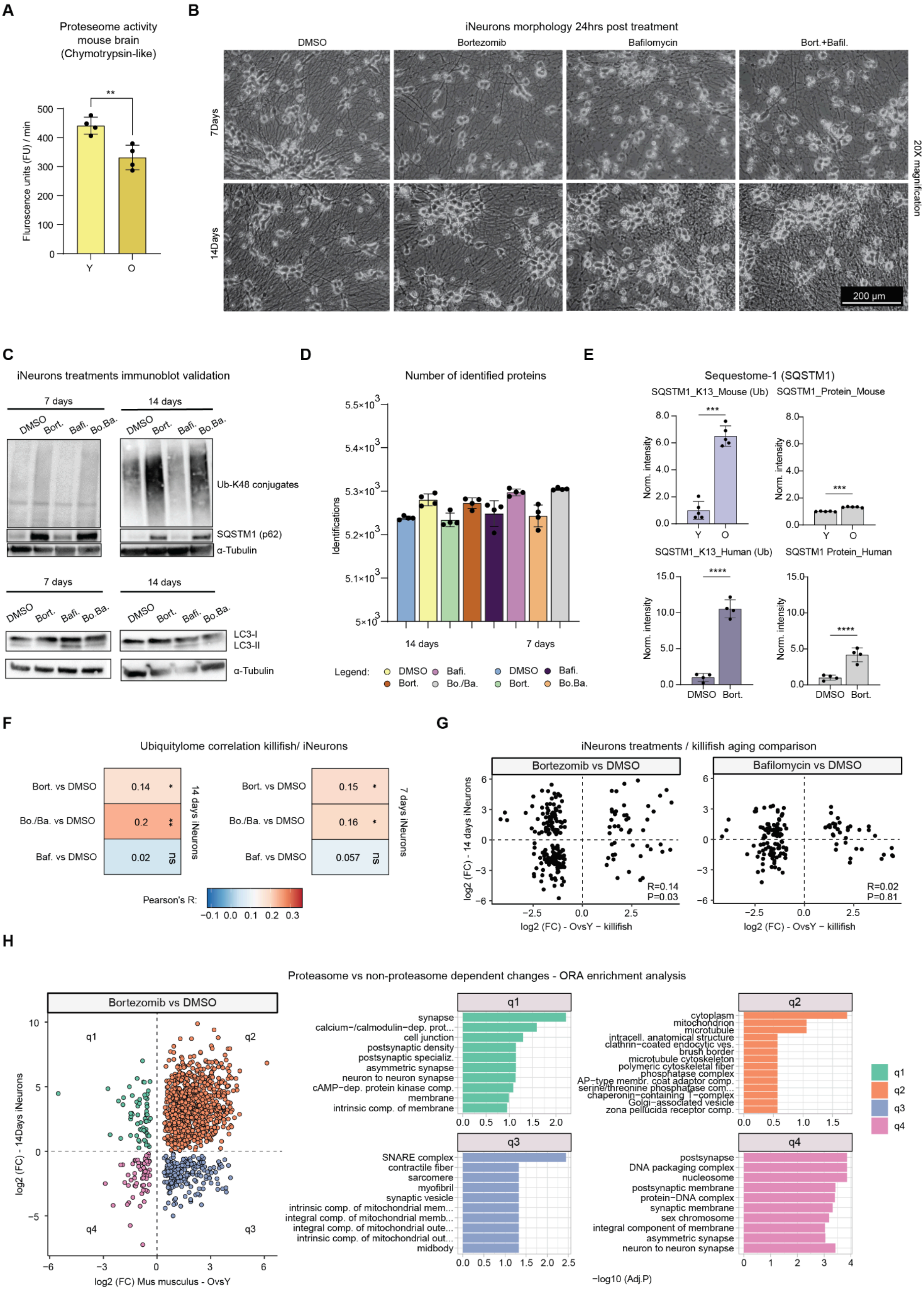
A
Proteseome activity
mouse brain
(Chymotrypsin-like)
Fluroscence units (FU) / min
Y
O
B
iNeurons morphology 24hrs post treatment
DMSO
Bortezomib
Bafilomycin
Bort.+Bafil.
7Days
14Days
20X magnification
200 µm
C
iNeurons treatments immunoblot validation
7 days
14 days
Ub-K48 conjugates
SQSTM1 (p62)
α-Tubulin
LC3-I
LC3-II
D
Number of identified proteins
Identifications
14 days
7 days
Legend:
DMSO Bafi. Bort. Bo./Ba.
E
Sequestome-1 (SQSTM1)
SQSTM1_K13_Mouse (Ub)
SQSTM1_Protein_Mouse
SQSTM1_K13_Human (Ub)
SQSTM1 Protein_Human
Norm. intensity
F
Ubiquitylome correlation killifish/ iNeurons
Bort. vs DMSO
Bo./Ba. vs DMSO
Baf. vs DMSO
14 days iNeurons
7 days iNeurons
Pearson's R:
G
iNeurons treatments / killifish aging comparison
Bortezomib vs DMSO
Bafilomycin vs DMSO
log2 (FC) - 14 days iNeurons
log2 (FC) - OvsY − killifish
R=0.14
P=0.03
R=0.02
P=0.81
H
Proteasome vs non-proteasome dependent changes - ORA enrichment analysis
Bortezomib vs DMSO
log2 (FC) - 14Days iNeurons
log2 (FC) Mus musculus - OvsY
q1
q2
q3
q4
−log10 (Adj.P)

**Figure S6. Proteasome inhibition in iNeurons and comparison with the aging datasets.**

A) Proteasome chymotrypsin-like activity in mouse brain lysates from young and old mice (N=4, biological replicates, unpaired *t*-test). B) Representative pictures of 7 days and 14 days iNeurons morphology 24 hours post-treatment (20X magnification, scale 200 μm). C) Immunoblots for K48 ubiquitin chains and LC3-II/LC3-I. Representative blots from N=3 biological replicates. D) Barplots showing the number of identified proteins in global proteome profiling of treated iNeurons. Data are shown as averages ± SD. E) Quantification of SQSTM1 (p62) ubiquitylation during aging in mouse brain (light purple) and upon proteasome inhibition in 14 days iNeurons (dark purple). Changes in total SQSTM1 protein level are shown in gray (N=5 for mouse and N=4 for iNeurons, biological replicates, Q values from Spectronaut for ubiquitylation and in Neurons proteome, Adj. P from limma's empirical Bayes moderated t-test for mouse proteome, data shown as averages ± SD). F) Correlation between changes of ubiquitylation observed during killifish brain aging and 14 days iNeurons treated with bortezomib or bafilomycin (P<0.05 in both datasets, two-sided Pearson's correlation test). G) Scatterplot comparing ubiquitylation changes observed in killifish brain aging and 14 days iNeurons treated with bortezomib (left panel) or bafilomycin (right panel) (P<0.05 in both datasets, two-sided Pearson's correlation test). H) Left: scatterplot comparing ubiquitylation changes observed in mouse brain aging and 14 days iNeurons treated with bortezomib (P<0.05 in both datasets). Same figure panel as Fig. 3H upper panel, reproduced here for orientation of the GO enrichment analysis. Right: GO over-representation analysis for each quadrant (q1:q4) of the scatterplot shown on the left. Analysis based on GO cellular component terms. Top 10 terms with P<0.05 (Fisher's Exact Test) for each quadrant are reported. Source data are provided as a Source Data file. Specific p values are listed in Supplementary Data 8. *Q / Adj.P ≤ 0.05; **Q / Adj.P ≤ 0.01, ***Q/ Adj.P ≤ 0.001, ****Q / Adj.P ≤ 0.0001.

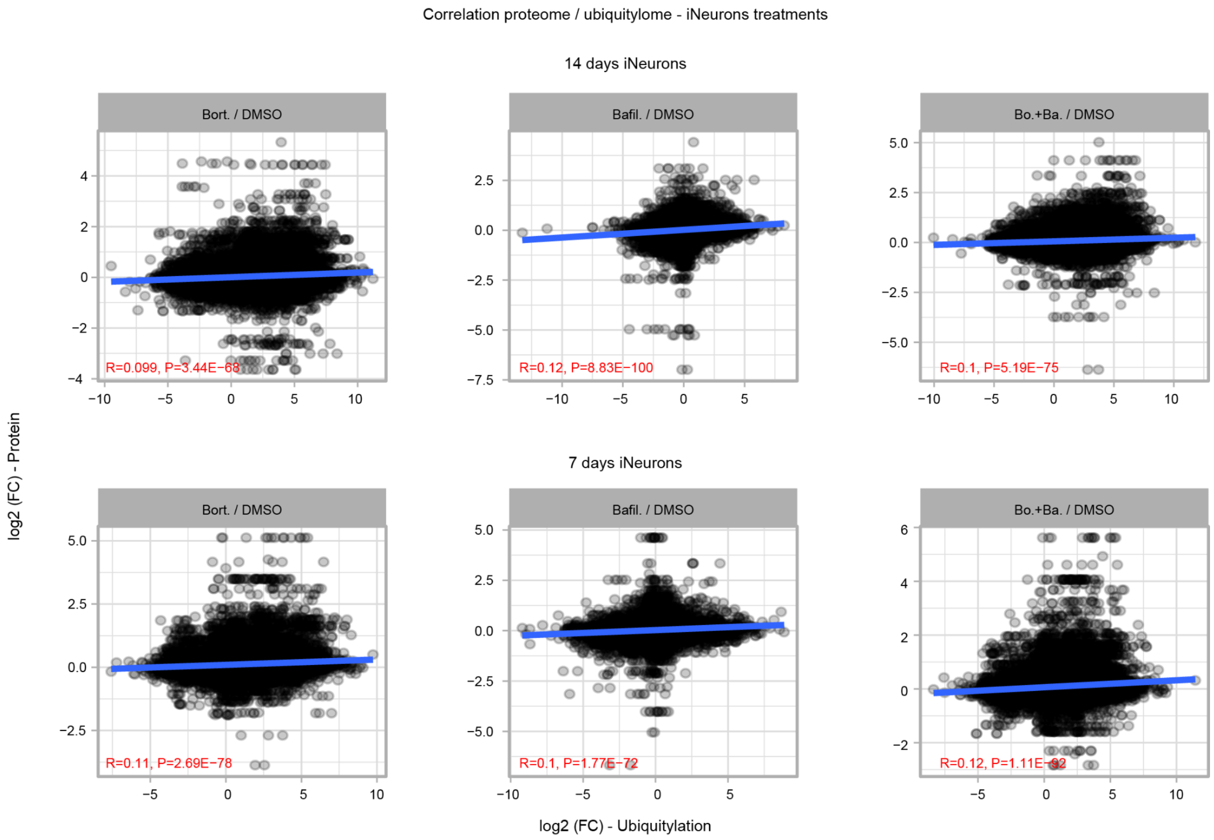


**Figure S7. Correlation of protein abundance and ubiquitylation changes in iNeurons**
Scatter plots comparing protein and ubiquitylated peptide abundance changes in iNeurons treated with bortezomib (Bort.), bafilomycin (Bafil.) or a combination of both drugs (Bo. + Ba.). Two-sided Pearson's correlation test (N=4, biological replicates).

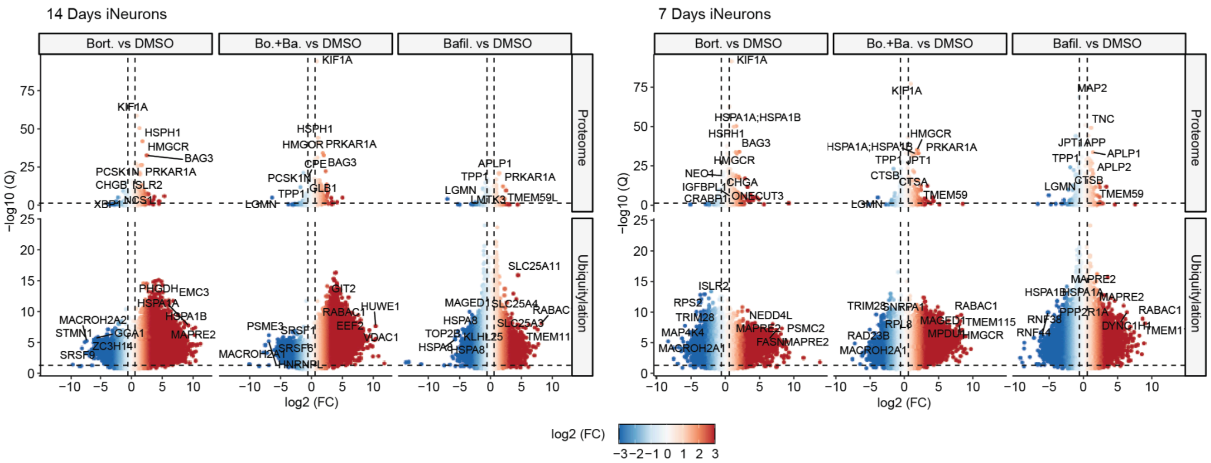


## Figure S8. Protein abundance and ubiquitylation changes in iNeurons

Volcano plots depicting changes in protein (upper panels) and ubiquitylated peptide abundance in iNeurons treated with bortezomib (Bort.), bafilomycin (Bafil.) or a combination of both drugs (Bo. + Ba.). Horizontal dashed line shows Q=0.05 while vertical lines show log2 (FC) = -0.58 or 0.58 (N=4, biological replicates, Q values from Spectronaut differential abundance analysis).

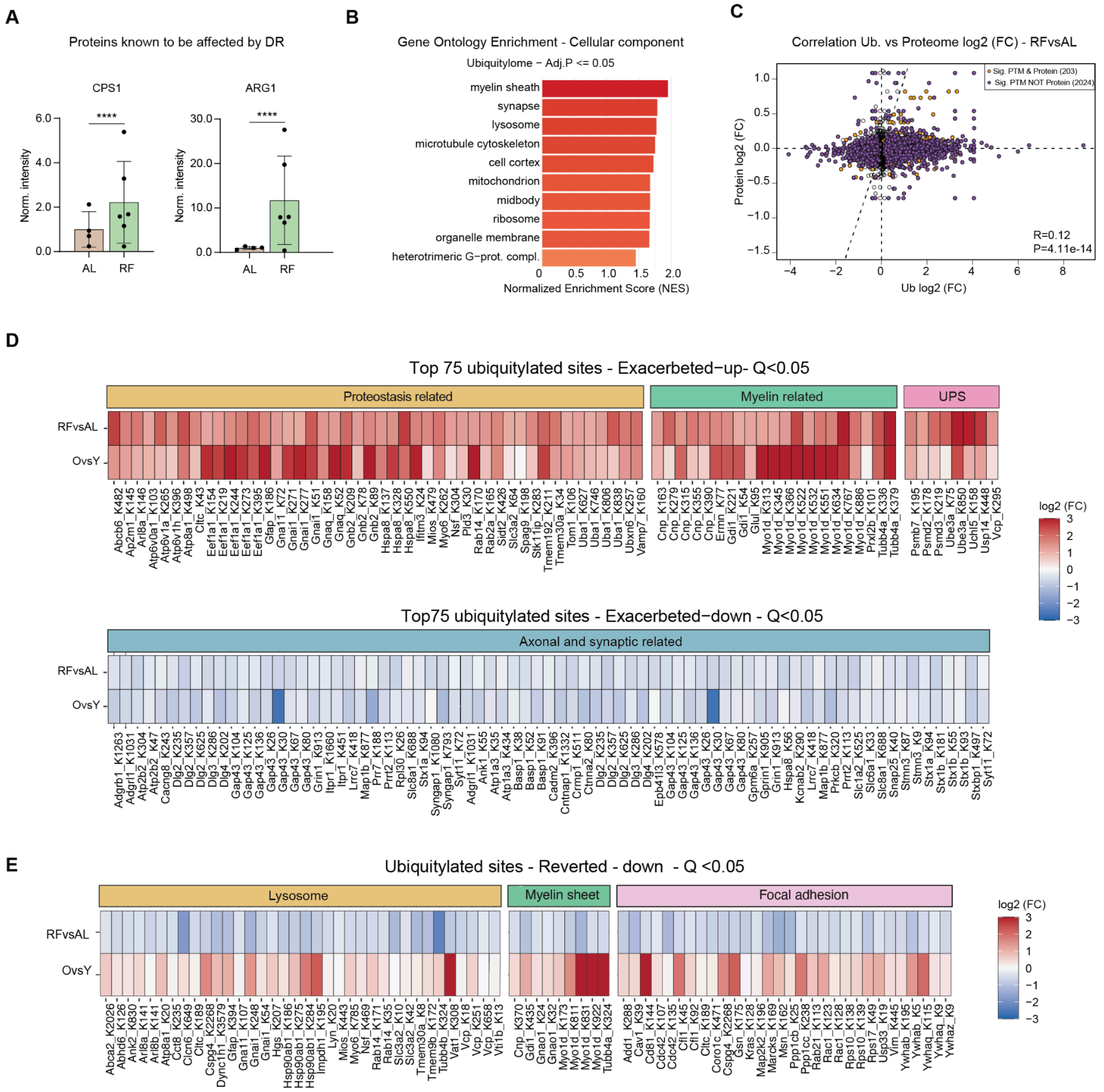


**Figure S9. Effect of dietary intervention on proteome and ubiquitylome of old mice**

A) CPS1 and ARG1 protein level changes upon re-feeding (RF) compared to the ad-libitum (AL) fed mice (N=4 for AL mice, N=6 for RF mice, biological replicates, Q values from Spectronaut, data shown as averages ± SD). B) Gene set enrichment analysis (GSEA) for ubiquitylated peptides affected by dietary intervention based on GO cellular component terms (Adj.P<0.05, weighted Kolmogorov–Smirnov test). C) Scatterplot illustrating the relationship between fold changes in protein abundance and corresponding ubiquitylated peptide levels following dietary intervention in old mice. Proteins showing significant age-related changes at both the total protein (Q<0.05 and absolute log2 (FC)>0.58) and ubiquitylated peptide level (Q<0.05) are highlighted in orange. Proteins with significant changes in ubiquitylation only (protein Q≥0.05 and ubiquitylated peptide Q<0.01) are shown in purple (N=4 for AL mice, N=6 for RF mice, biological replicates). Q values from Spectronaut differential abundance analysis. Two-sided Pearson's correlation test. D) Heatmap highlighting age-related changes in ubiquitylation that are exacerbated by dietary intervention (Q<0.05 in response to dietary

intervention and Adj.P<0.05 in response to aging and same log2 (FC)). Top 75 affected ubiquitylation sites (ranked according to fold change) displaying increased (top) or decreased (bottom) abundance are shown. E) Heatmap highlighting age-related changes in ubiquitylation that are reverted by dietary intervention (Q<0.05 in response to dietary intervention and of opposite log2 (FC) in response to aging). *Q / Adj.P ≤ 0.05; **Q / Adj.P ≤ 0.01, ***Q/ Adj.P ≤ 0.001, ****Q / Adj.P ≤ 0.0001.

**Supplementary Table 1: MS data used for library generation on Spectronaut Software.**

| ***Dataset*** | ***Data used for the library*** | ***Library size*** |
|---|---|---|
| Ubiquitin- Mouse | DIA / DDA | 41949 modified peptides (5950 proteins) |
| Ubiquitin- iNeurons | DIA / DDA | 69359 modified peptides (7374 proteins) |
| Phosphorylation - Mouse | DIA | 40711 modified peptides (3630 proteins) |
| Acetylation - Mouse | DIA / DDA | 28952 modified peptides (4451 proteins) |
| iNeu Proteome | DIA | 6165 proteins |

**Supplementary Table 2: Number of corrected ubiquitylation sites using total proteome abundance.**

| **Dataset** | **Total n. of sites** | **% corrected using proteome** | **Sites not corrected (reference protein not detected)** |
|---|---|---|---|
| Ubiquityl. mouse brain | 6683 | 95.5% | 307 |
| Ubiquityl. mouse liver | 3779 | 89.0% | 415 |
| Ubiquityl. killifish brain | 3133 | 94.7% | 166 |
