# Supplementary material for: Aging and diet alter the protein ubiquitylation landscape in the mouse brain
Source: Nat Commun. 2025 Jun 6;16:5266. doi: 10.1038/s41467-025-60542-6 (PMC12144301; doi:10.1038/s41467-025-60542-6)
Supplement: Supplementary file 2 — Description of Additional Supplementary Files [file 41467_2025_60542_MOESM2_ESM.pdf]

Description of Additional Supplementary Files

File Name: Supplementary Data 1

Description: Proteomics (global, ubiquitylation, phosphorylation, and acetylation) and transcriptomics changes in mouse brain aging. Related to Figure 1 and Supplementary Figs. 1, 2, and 3.

File Name: Supplementary Data 2

Description: Proteomics (global and ubiquitylation) and transcriptomics changes in mouse liver aging. Related to Supplementary Fig. 4.

File Name: Supplementary Data 3

Description: Mouse and killifish ubiquitylated sites alignment and age-related ubiquitylation changes upon correction for protein abundance. Related to Figure 2 and Supplementary Fig. 5.

File Name: Supplementary Data 4

Description: Proteome and ubiquitylome changes induced in iNeurons by bafilomycin and bortezomib treatment. Related to Figure 3 and Supplementary Fig. 6, 7 and 8.

File Name: Supplementary Data 5

Description: Human (iNeurons), mouse and killifish ubiquitylated sites alignment and ubiquitylation changes upon correction for protein abundance. Neurodegeneration-associated genes are annotated. Related to Figure 3 and Supplementary Fig. 6.

File Name: Supplementary Data 6

Description: Absolute quantification of ubiquitin-chain linkages using AQUA-PRM in mouse brain aging and iNeurons treatments. Related to Figure 4.

File Name: Supplementary Data 7

Description: Proteome and ubiquitylome changes induced by dietary intervention in old mice. Related to Figure 5 and Supplementary Fig. 9

File Name: Supplementary Data 8

Description: List of specific p-values. Related to all Figures.
