# Supplementary material for: Aging and diet alter the protein ubiquitylation landscape in the mouse brain
Source: Nat Commun. 2025 Jun 6;16:5266. doi: 10.1038/s41467-025-60542-6 (PMC12144301; doi:10.1038/s41467-025-60542-6)
Supplement: Supplementary file 11 — Reporting Summary [file 41467_2025_60542_MOESM11_ESM.pdf]

nature portfolio

Corresponding author(s): Alessandro Ori

Last updated by author(s): Apr 25, 2025

Reporting Summary

Nature Portfolio wishes to improve the reproducibility of the work that we publish. This form provides structure for consistency and transparency in reporting. For further information on Nature Portfolio policies, see our Editorial Policies and the Editorial Policy Checklist.

Statistics

For all statistical analyses, confirm that the following items are present in the figure legend, table legend, main text, or Methods section.

n/a	Confirmed	
☐	☒	The exact sample size (*n*) for each experimental group/condition, given as a discrete number and unit of measurement
☐	☒	A statement on whether measurements were taken from distinct samples or whether the same sample was measured repeatedly
☐	☒	The statistical test(s) used AND whether they are one- or two-sided<br>*Only common tests should be described solely by name; describe more complex techniques in the Methods section.*
☒	☐	A description of all covariates tested
☐	☒	A description of any assumptions or corrections, such as tests of normality and adjustment for multiple comparisons
☐	☒	A full description of the statistical parameters including central tendency (e.g. means) or other basic estimates (e.g. regression coefficient) AND variation (e.g. standard deviation) or associated estimates of uncertainty (e.g. confidence intervals)
☐	☒	For null hypothesis testing, the test statistic (e.g. *F*, *t*, *r*) with confidence intervals, effect sizes, degrees of freedom and *P* value noted<br>*Give P values as exact values whenever suitable.*
☒	☐	For Bayesian analysis, information on the choice of priors and Markov chain Monte Carlo settings
☒	☐	For hierarchical and complex designs, identification of the appropriate level for tests and full reporting of outcomes
☐	☒	Estimates of effect sizes (e.g. Cohen's *d*, Pearson's *r*), indicating how they were calculated

Our web collection on statistics for biologists contains articles on many of the points above.

Software and code

Policy information about availability of computer code

Data collection	ImageLab 6.1.0, ZEN 3.2, The Xcalibur v4.0
Data analysis	Spectronaut Pulsar 18.2.33, Spectrodive 11.12.23, R studio 4.1, ImageLab 6.1.0, Graphpad Prism 9.0.2, Tune v2.1, ProteomeDiscoverer v2.0

For manuscripts utilizing custom algorithms or software that are central to the research but not yet described in published literature, software must be made available to editors and reviewers. We strongly encourage code deposition in a community repository (e.g. GitHub). See the Nature Portfolio guidelines for submitting code & software for further information.

Data

Policy information about availability of data

All manuscripts must include a data availability statement. This statement should provide the following information, where applicable:

- Accession codes, unique identifiers, or web links for publicly available datasets
- A description of any restrictions on data availability
- For clinical datasets or third party data, please ensure that the statement adheres to our policy

The proteomic data have been deposited and released on https://massive.ucsd.edu with these identifiers:

Dataset and Accession ID:
Ubiquitylation mouse brain aging:

MSV000093686

Acetylation mouse brain aging:
MSV000093689

Phosphorylation mouse brain aging:
MSV000093687

Whole proteome mouse brain aging:
MSV000093690

AQUA-PRM ub-chains brain aging mouse:
MSV000093996

Ubiquitylation mouse liver aging:
MSV000096232

Whole proteome mouse liver aging:
MSV000096231

Ubiquitylation iNeurons:
MSV000093691

Whole proteome iNeurons:
MSV000093693

AQUA-PRM ub-chains iNeurons:
MSV000096233

Ubiquitylation mouse brain dietary intervention:
MSV000096229

Whole proteome mouse brain dietary intervention:
MSV000096226

The RNA-seq mouse brain aging data have been deposited on https://www.ncbi.nlm.nih.gov/geo/ with the following identifier:
GSE253375

Research involving human participants, their data, or biological material

Policy information about studies with human participants or human data. See also policy information about sex, gender (identity/presentation), and sexual orientation and race, ethnicity and racism.

Reporting on sex and gender	N/A
Reporting on race, ethnicity, or other socially relevant groupings	N/A
Population characteristics	N/A
Recruitment	N/A
Ethics oversight	N/A

Note that full information on the approval of the study protocol must also be provided in the manuscript.

Field-specific reporting

Please select the one below that is the best fit for your research. If you are not sure, read the appropriate sections before making your selection.

☒ Life sciences ☐ Behavioural & social sciences ☐ Ecological, evolutionary & environmental sciences

For a reference copy of the document with all sections, see nature.com/documents/nr-reporting-summary-flat.pdf

Life sciences study design

All studies must disclose on these points even when the disclosure is negative.

Sample size	No statistical methods were used to pre-determine sample sizes.
Data exclusions	No data were excluded from the analyses except for technical dropouts when stated.
Replication	All available data were used to maximize statistical power of the analysis therefore we did not repeat the analysis.
Randomization	Samples were randomized for proteomic data acquisition.
Blinding	Blinding is not applicable to this study as this study is observational.

Reporting for specific materials, systems and methods

We require information from authors about some types of materials, experimental systems and methods used in many studies. Here, indicate whether each material, system or method listed is relevant to your study. If you are not sure if a list item applies to your research, read the appropriate section before selecting a response.

Materials & experimental systems

n/a	Involved in the study	
☐	☒	Antibodies
☐	☒	Eukaryotic cell lines
☒	☐	Palaeontology and archaeology
☐	☒	Animals and other organisms
☒	☐	Clinical data
☒	☐	Dual use research of concern
☒	☐	Plants

Methods

n/a	Involved in the study	
☒	☐	ChIP-seq
☒	☐	Flow cytometry
☒	☐	MRI-based neuroimaging

Antibodies

Antibodies used	monoclonal mouse antibody, Ubiquitin FK2, Enzo Life Sciences, BML-PW8810, 1:1000<br>Rabbit Polyclonal SQSTM1 / p62 antibody, Abcam, ab91526, 1:1000<br>monoclonal rabbit antibody Anti-Ubiquitin Antibody, Lys48-Specific, clone Apu2, MilliporeSigma, 05-1307, 1:1000<br>polyclonal rabbit antibody LC3B Cell Signaling Technology, 2775, 1:1000<br>Monoclonal mouse Anti-α-Tubulin antibody, Merk, T9026, 1:1000
Validation	All antibodies were commercially available. Antibody clones were selected based on validation data shown on the manufacturer's website.

Eukaryotic cell lines

Policy information about cell lines and Sex and Gender in Research

Cell line source(s)	WTC11 hiPSC Cell Line was a kind gift from Ward Lab
Authentication	None of the cell lines used were authenticated
Mycoplasma contamination	All cells were regularly checked for negative Mycoplasma contamination.
Commonly misidentified lines (See ICLAC register)	We decided to use WTC11 human iPSC-derived neurons (iNeurons) (Wang et al., 2017) because they are been established as a reference in vitro model for human age-associated neurodegenerative disorders (Pantazis et al., 2022) and have previously been used for proteome-wide investigations of protein ubiquitylation (Antico et al., 2021; Ordureau et al., 2020).

Animals and other research organisms

Policy information about studies involving animals; ARRIVE guidelines recommended for reporting animal research, and Sex and Gender in Research

Laboratory animals	All wild-type mice were C57BL/6J obtained from Janvier Labs (Le Genest-Saint-Isle, France) or internal breeding at FLI. All animals were kept in a specific pathogen-free animal facility with a 12h light/dark cycle at a temperature of 20°C±2 and humidity of 55% ± 15. Young mice were aged three or four months, and old mice were aged 33 months. During the experiment, Mice had unlimited access to food (ssniff, Soest, Germany).

Wild animals	No wild animals were used for this study
Reporting on sex	Only male mice were used in this study.
Field-collected samples	This study did not involve samples collected in the field.
Ethics oversight	All experiments were carried out according to the guidelines from Directive 2010/63/EU of the European Parliament on the protection of animals used for scientific purposes. The protocols of animal maintenance and euthanasia were approved by the local authorities for animal welfare in the State of Thuringia, Germany

Note that full information on the approval of the study protocol must also be provided in the manuscript.

Plants

Seed stocks	N/A
Novel plant genotypes	N/A
Authentication	N/A
