# Supplementary material for: Aging and diet alter the protein ubiquitylation landscape in the mouse brain
Source: Nat Commun. 2025 Jun 6;16:5266. doi: 10.1038/s41467-025-60542-6 (PMC12144301; doi:10.1038/s41467-025-60542-6)
Supplement: Supplementary file 13 — Source Data [file 41467_2025_60542_MOESM13_ESM.zip › Source_data/Figure_5/G/G.pdf]

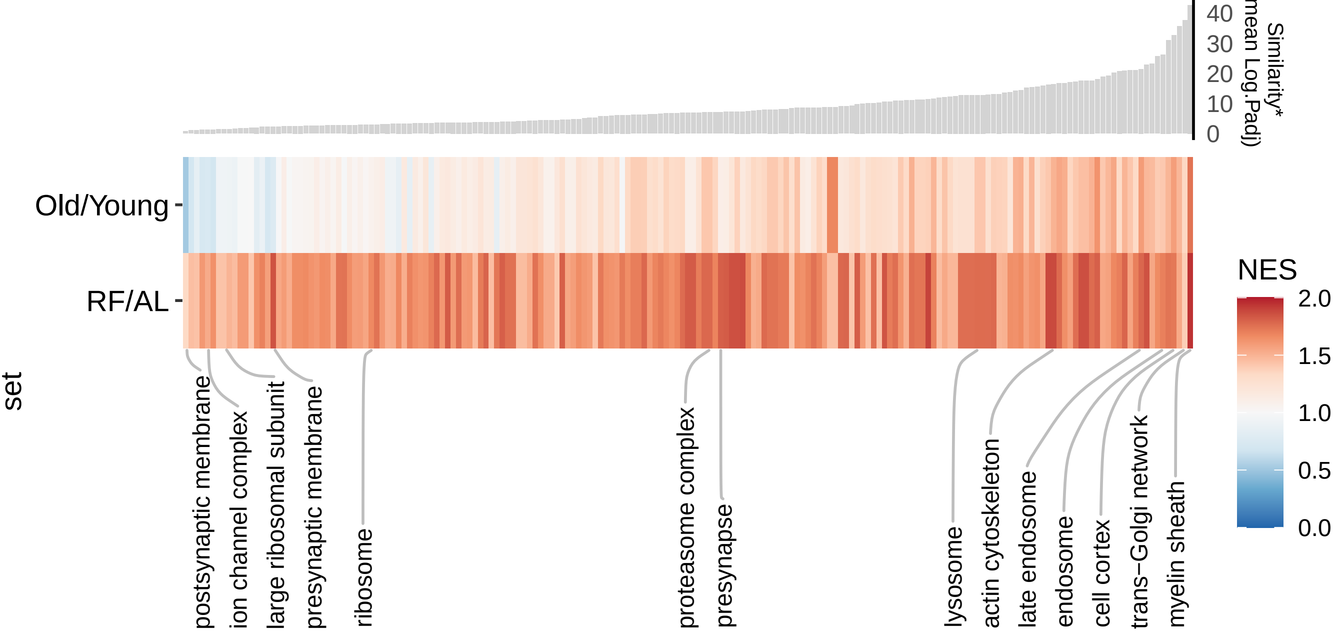

40
30
20
10
0
Similarity*
mean Log.Padj)
Old/Young
RF/AL
set
NES
2.0
1.5
1.0
0.5
0.0
postsynaptic membrane
ion channel complex
large ribosomal subunit
presynaptic membrane
ribosome
proteasome complex
presynapse
lysosome
actin cytoskeleton
late endosome
endosome
cell cortex
trans−Golgi network
myelin sheath
