# Supplementary material for: Aging and diet alter the protein ubiquitylation landscape in the mouse brain
Source: Nat Commun. 2025 Jun 6;16:5266. doi: 10.1038/s41467-025-60542-6 (PMC12144301; doi:10.1038/s41467-025-60542-6)
Supplement: Supplementary file 13 — Source Data [file 41467_2025_60542_MOESM13_ESM.zip › Source_data/Figure_4/C/C_iNeu_data.pdf]

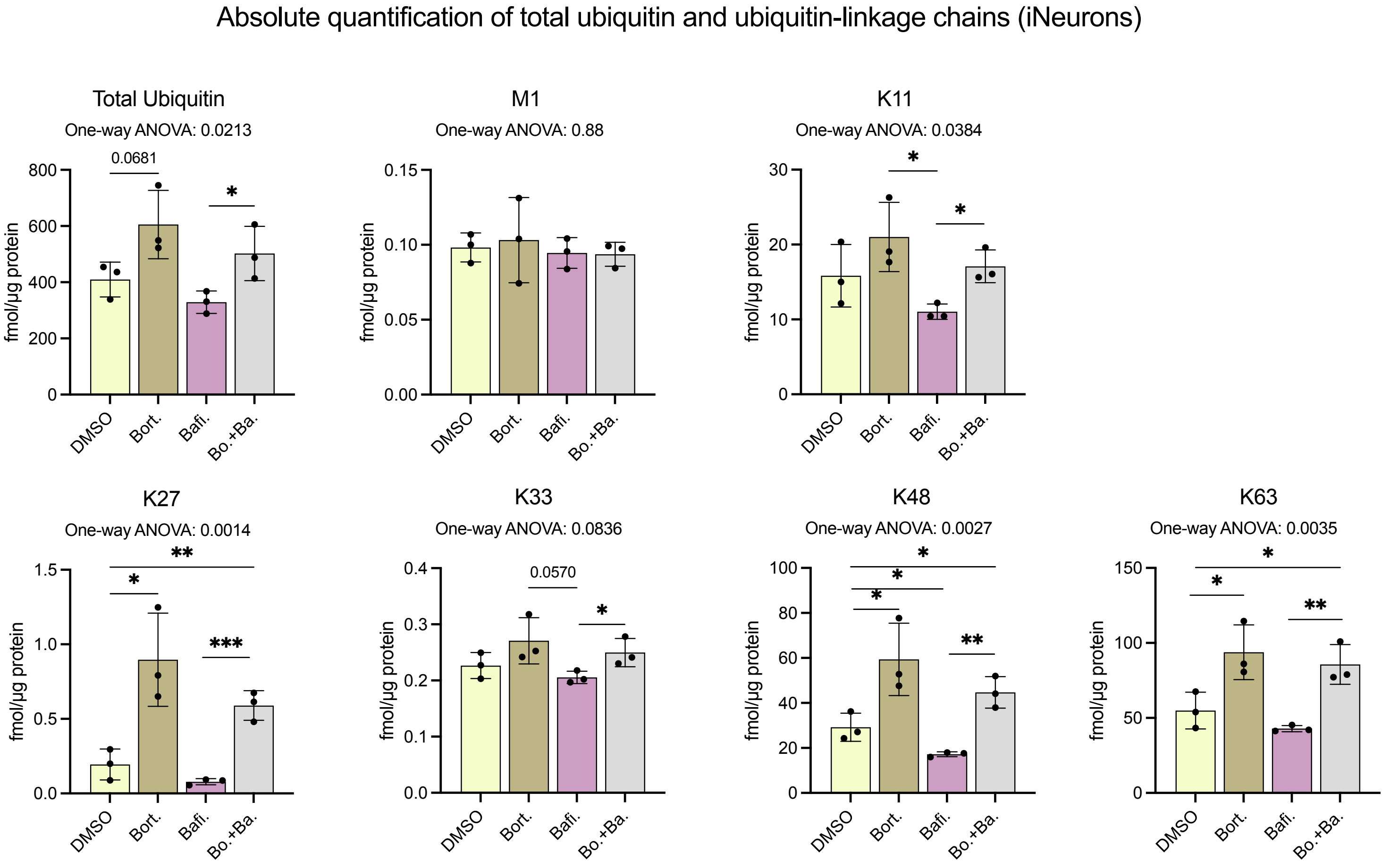

Absolute quantification of total ubiquitin and ubiquitin-linkage chains (iNeurons)
Total Ubiquitin
One-way ANOVA: 0.0213
0.0681
*
fmol/µg protein
M1
One-way ANOVA: 0.88
K11
One-way ANOVA: 0.0384
K27
One-way ANOVA: 0.0014
**
***
K33
One-way ANOVA: 0.0836
0.0570
K48
One-way ANOVA: 0.0027
K63
One-way ANOVA: 0.0035
DMSO
Bort.
Bafi.
Bo.+Ba.
