# Supplementary material for: Aging and diet alter the protein ubiquitylation landscape in the mouse brain
Source: Nat Commun. 2025 Jun 6;16:5266. doi: 10.1038/s41467-025-60542-6 (PMC12144301; doi:10.1038/s41467-025-60542-6)
Supplement: Supplementary file 13 — Source Data [file 41467_2025_60542_MOESM13_ESM.zip › Source_data/Figure_3/I/I.pdf]

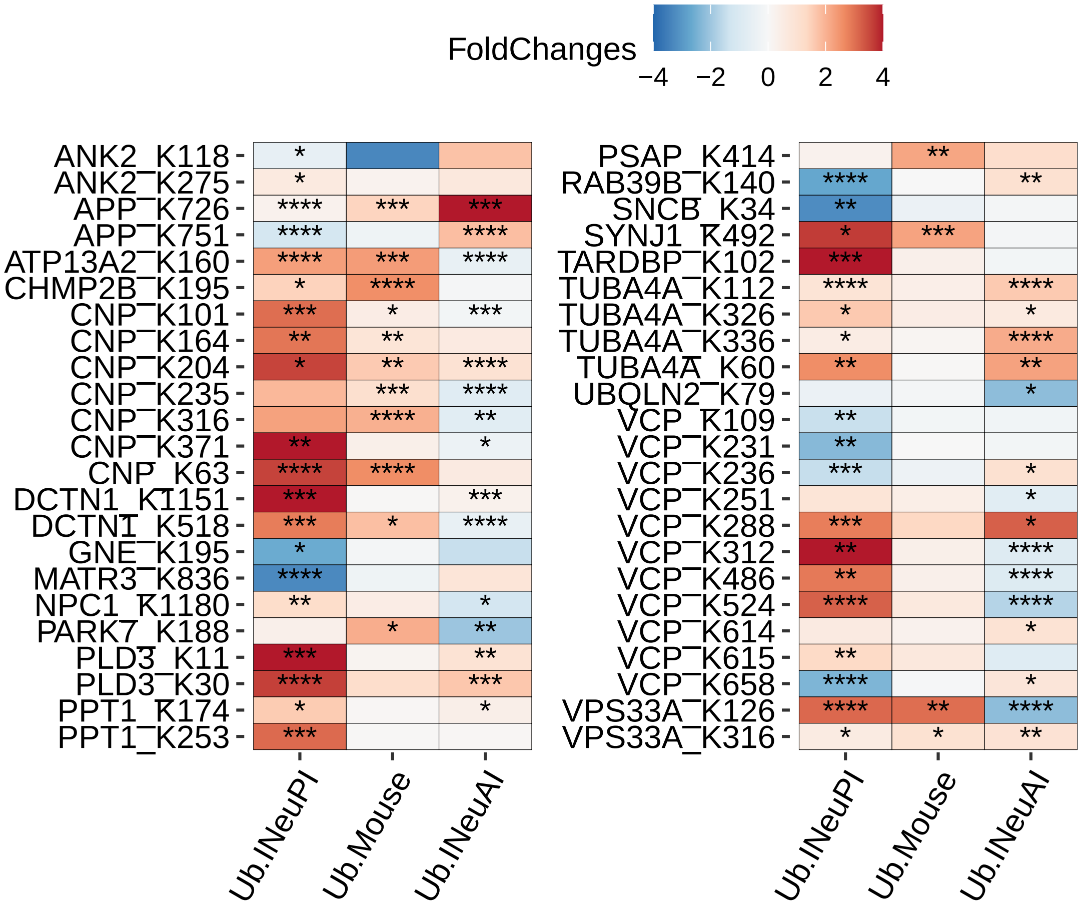
FoldChanges
−4
−2
0
2
4
ANK2_K118
ANK2_K275
APP_K726
APP_K751
ATP13A2_K160
CHMP2B_K195
CNP_K101
CNP_K164
CNP_K204
CNP_K235
CNP_K316
CNP_K371
CNP_K63
DCTN1_K1151
DCTN1_K518
GNE_K195
MATR3_K836
NPC1_K1180
PARK7_K188
PLD3_K11
PLD3_K30
PPT1_K174
PPT1_K253
PSAP_K414
RAB39B_K140
SNCB_K34
SYNJ1_K492
TARDBP_K102
TUBA4A_K112
TUBA4A_K326
TUBA4A_K336
TUBA4A_K60
UBQLN2_K79
VCP_K109
VCP_K231
VCP_K236
VCP_K251
VCP_K288
VCP_K312
VCP_K486
VCP_K524
VCP_K614
VCP_K615
VCP_K658
VPS33A_K126
VPS33A_K316
Ub.INeuPI
Ub.Mouse
Ub.INeuAI
