# Supplementary material for: Aging and diet alter the protein ubiquitylation landscape in the mouse brain
Source: Nat Commun. 2025 Jun 6;16:5266. doi: 10.1038/s41467-025-60542-6 (PMC12144301; doi:10.1038/s41467-025-60542-6)
Supplement: Supplementary file 13 — Source Data [file 41467_2025_60542_MOESM13_ESM.zip › Source_data/Figure_S6/F/F.pdf]

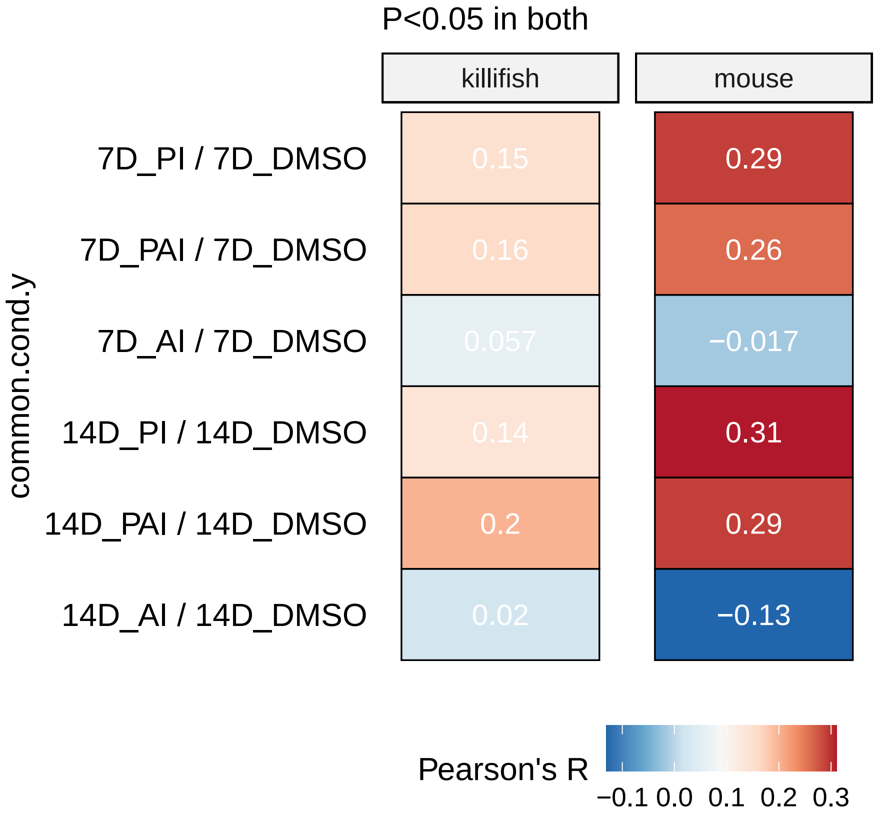
P<0.05 in both
killifish
mouse
common.cond.y
7D_PI / 7D_DMSO
7D_PAI / 7D_DMSO
7D_AI / 7D_DMSO
14D_PI / 14D_DMSO
14D_PAI / 14D_DMSO
14D_AI / 14D_DMSO
0.15
0.29
0.16
0.26
0.057
−0.017
0.14
0.31
0.2
0.29
0.02
−0.13
Pearson's R
−0.1 0.0 0.1 0.2 0.3
