# Supplementary material for: Aging and diet alter the protein ubiquitylation landscape in the mouse brain
Source: Nat Commun. 2025 Jun 6;16:5266. doi: 10.1038/s41467-025-60542-6 (PMC12144301; doi:10.1038/s41467-025-60542-6)
Supplement: Supplementary file 13 — Source Data [file 41467_2025_60542_MOESM13_ESM.zip › Source_data/Figure_S2/C/Ubiquitin_Report_research_ori 2021-03-19_16h38m04s_Exposure_29.4sec.pdf]

# Image Report: research_ori
# 2021-03-19_16h38m04s_Exposure_29.4sec

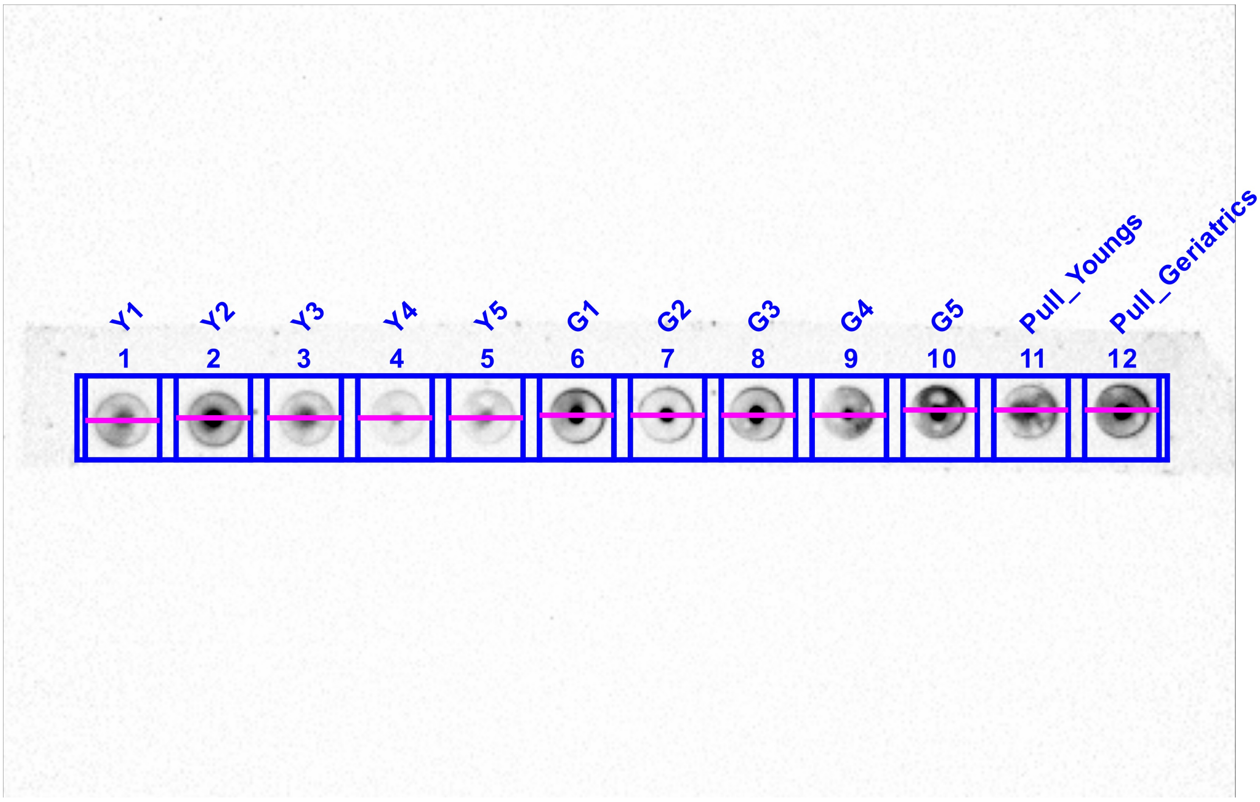


**S:\Ori\Antonio\DotBlot\180321_Brains_Mice_FK2_K63_K48_K27_ab19247\FK2\research_ori 2021-03-19_16h38m04s_Exposure_29.4sec.scn**

## Acquisition Information

| | |
|---|---|
| Imager | ChemiDoc XRS+ |
| Exposure Time (sec) | 29.444 (Signal Accumulation) |
| Flat Field | Applied (Lens) |
| Serial Number | 721BR12704 |
| Software Version | 6.0.1.34 |
| Application | Chemi |
| Excitation Source | No Illumination |
| Emission Filter | No Filter |
| Binning | 3x3 |

## Image Information

| | |
|---|---|
| Acquisition Date | 3/19/2021 4:38:57 PM |
| User Name | research_ori |
| Image Area (mm) | X: 124.0 Y: 92.5 |
| Pixel Size (µm) | X: 267.2 Y: 267.2 |
| Data Range (Int) | 0 - 46272 |

## Analysis Settings

| | |
|---|---|
| Detection | Lane detection:<br>Manually created lanes<br><br>Band detection:<br><br>Manually adjusted bands<br><br>Lane Background Subtraction:<br>Lane background subtracted with disk size: 9.4<br><br>Lane width: 7.48 mm |

## Lane Statistics

| Lane No. | Adj. Total Band Vol. (Int) | Total Band Vol. (Int) | Adj. Total Lane Vol. (Int) | Total Lane Vol. (Int) | Bkgd. Vol. (Int) | Norm. Factor |
|---|---|---|---|---|---|---|
| 1 | 1,715,028 | 2,071,496 | 1,716,008 | 2,138,276 | 422,268 | N/A |
| 2 | 2,756,936 | 3,097,864 | 2,763,292 | 3,170,300 | 407,008 | N/A |
| 3 | 1,510,152 | 1,767,080 | 1,513,680 | 1,819,216 | 305,536 | N/A |
| 4 | 552,608 | 794,948 | 556,780 | 867,916 | 311,136 | N/A |
| 5 | 814,436 | 1,067,612 | 825,944 | 1,151,304 | 325,360 | N/A |
| 6 | 2,689,596 | 2,909,648 | 2,702,700 | 2,972,004 | 269,304 | N/A |
| 7 | 1,437,772 | 1,697,052 | 1,446,676 | 1,776,852 | 330,176 | N/A |
| 8 | 2,466,604 | 2,727,844 | 2,477,468 | 2,824,220 | 346,752 | N/A |
| 9 | 1,937,936 | 2,190,608 | 1,949,108 | 2,283,736 | 334,628 | N/A |
| 10 | 3,221,456 | 3,528,420 | 3,231,060 | 3,618,132 | 387,072 | N/A |
| 11 | 2,353,428 | 2,808,624 | 2,356,648 | 2,919,616 | 562,968 | N/A |
| 12 | 3,291,820 | 3,907,008 | 3,295,992 | 4,050,592 | 754,600 | N/A |

## Lane And Band Analysis

### Lane 1 - Y1

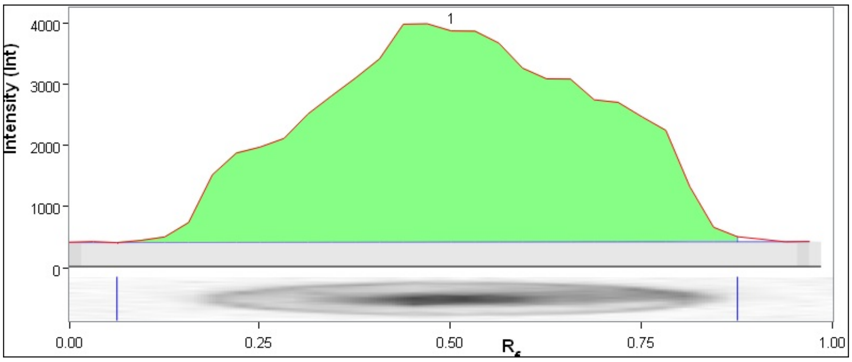


| Band No. | Band Label | Mol. Wt. (KDa) | Relative Front | Adj. Volume (Int) | Volume (Int) | Abs. Quant. | Rel. Quant. | Band % | Lane % |
|---|---|---|---|---|---|---|---|---|---|
| 1 | | N/A | 0.531 | 1,715,028 | 2,071,496 | N/A | N/A | 100.0 | 99.9 |

| | |
|---|---|
| Lane Background | Lane background subtracted with disk size: 9.4 |
| Lane Width | 7.48 mm |

### Lane 2 - Y2

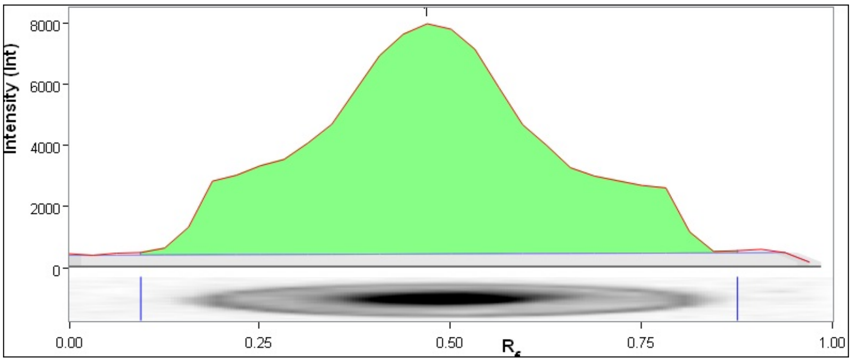


| Band No. | Band Label | Mol. Wt. (KDa) | Relative Front | Adj. Volume (Int) | Volume (Int) | Abs. Quant. | Rel. Quant. | Band % | Lane % |
|---|---|---|---|---|---|---|---|---|---|
| 1 | | N/A | 0.500 | 2,756,936 | 3,097,864 | N/A | N/A | 100.0 | 99.8 |

| | |
|---|---|
| Lane Background | Lane background subtracted with disk size: 9.4 |
| Lane Width | 7.48 mm |

## Lane 3 - Y3

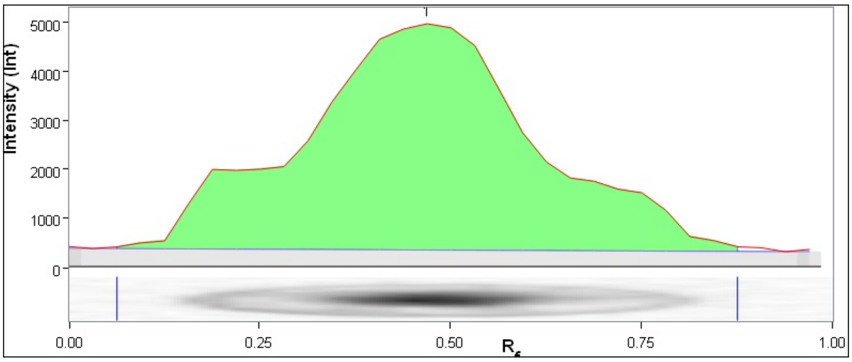


| Band No. | Band Label | Mol. Wt. (KDa) | Relative Front | Adj. Volume (Int) | Volume (Int) | Abs. Quant. | Rel. Quant. | Band % | Lane % |
|---|---|---|---|---|---|---|---|---|---|
| 1 | | N/A | 0.500 | 1,510,152 | 1,767,080 | N/A | N/A | 100.0 | 99.8 |

| | |
|---|---|
| Lane Background | Lane background subtracted with disk size: 9.4 |
| Lane Width | 7.48 mm |

## Lane 4 - Y4

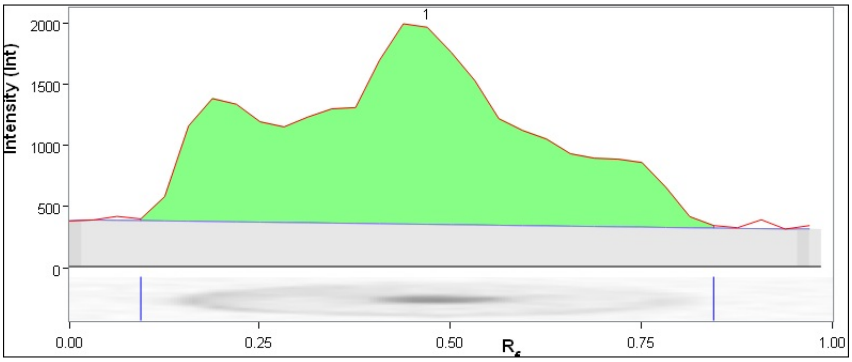

| Band No. | Band Label | Mol. Wt. (KDa) | Relative Front | Adj. Volume (Int) | Volume (Int) | Abs. Quant. | Rel. Quant. | Band % | Lane % |
|---|---|---|---|---|---|---|---|---|---|
| 1 | | N/A | 0.500 | 552,608 | 794,948 | N/A | N/A | 100.0 | 99.3 |

| | |
|---|---|
| Lane Background | Lane background subtracted with disk size: 9.4 |
| Lane Width | 7.48 mm |

### Lane 5 - Y5

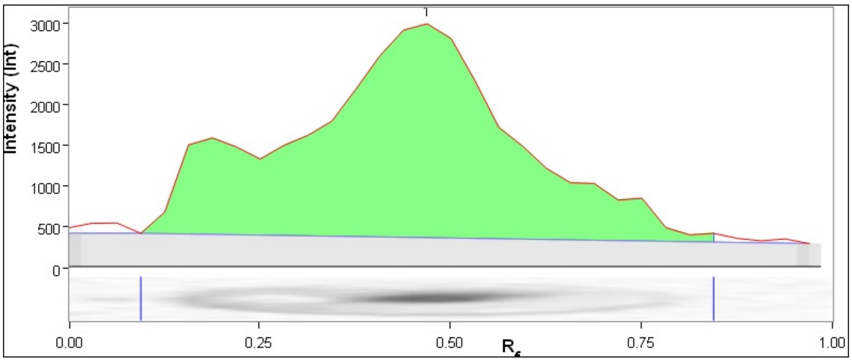


| Band No. | Band Label | Mol. Wt. (KDa) | Relative Front | Adj. Volume (Int) | Volume (Int) | Abs. Quant. | Rel. Quant. | Band % | Lane % |
|---|---|---|---|---|---|---|---|---|---|
| 1 | | N/A | 0.500 | 814,436 | 1,067,612 | N/A | N/A | 100.0 | 98.6 |

| | |
|---|---|
| Lane Background | Lane background subtracted with disk size: 9.4 |
| Lane Width | 7.48 mm |

### Lane 6 - G1

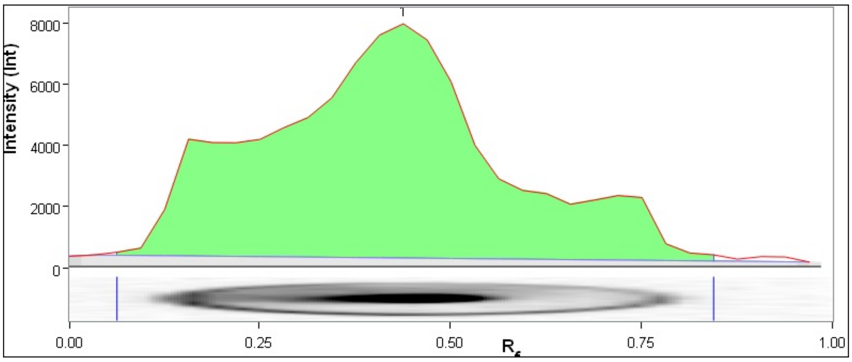


| Band No. | Band Label | Mol. Wt. (KDa) | Relative Front | Adj. Volume (Int) | Volume (Int) | Abs. Quant. | Rel. Quant. | Band % | Lane % |
|---|---|---|---|---|---|---|---|---|---|
| 1 | | N/A | 0.469 | 2,689,596 | 2,909,648 | N/A | N/A | 100.0 | 99.5 |

| | |
|---|---|
| Lane Background | Lane background subtracted with disk size: 9.4 |
| Lane Width | 7.48 mm |

### Lane 7 - G2

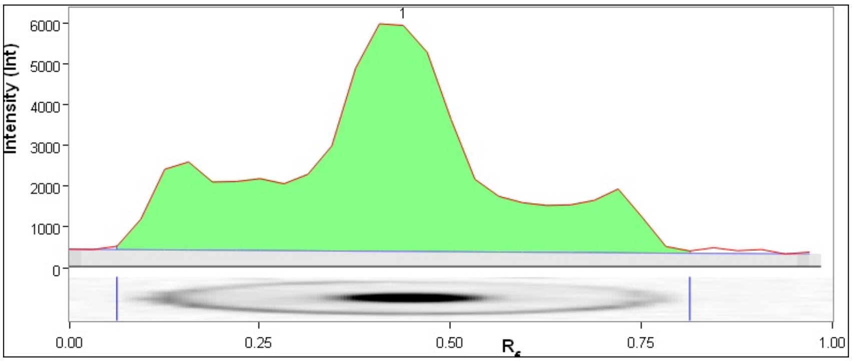


| Band No. | Band Label | Mol. Wt. (KDa) | Relative Front | Adj. Volume (Int) | Volume (Int) | Abs. Quant. | Rel. Quant. | Band % | Lane % |
|---|---|---|---|---|---|---|---|---|---|
| 1 | | N/A | 0.469 | 1,437,772 | 1,697,052 | N/A | N/A | 100.0 | 99.4 |

| | |
|---|---|
| Lane Background | Lane background subtracted with disk size: 9.4 |
| Lane Width | 7.48 mm |

## Lane 8 - G3

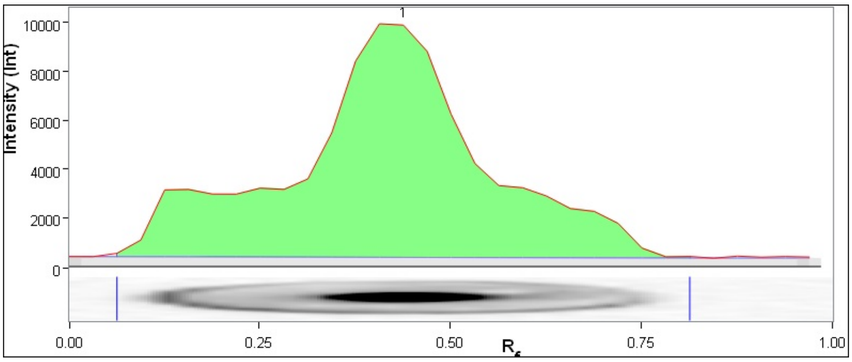


| Band No. | Band Label | Mol. Wt. (KDa) | Relative Front | Adj. Volume (Int) | Volume (Int) | Abs. Quant. | Rel. Quant. | Band % | Lane % |
|---|---|---|---|---|---|---|---|---|---|
| 1 | | N/A | 0.469 | 2,466,604 | 2,727,844 | N/A | N/A | 100.0 | 99.6 |

| | |
|---|---|
| Lane Background | Lane background subtracted with disk size: 9.4 |
| Lane Width | 7.48 mm |

## Lane 9 - G4

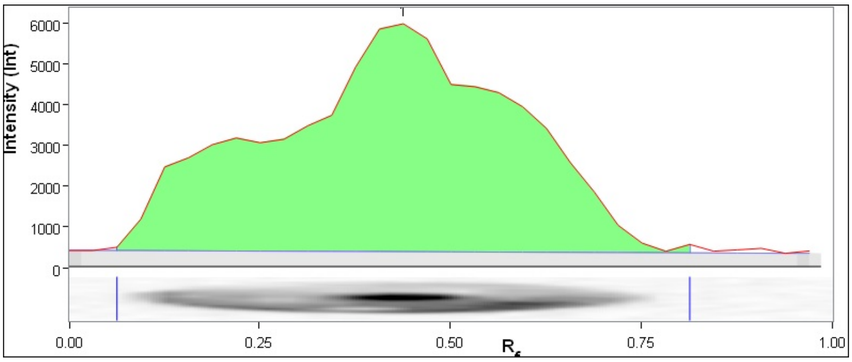

| Band No. | Band Label | Mol. Wt. (KDa) | Relative Front | Adj. Volume (Int) | Volume (Int) | Abs. Quant. | Rel. Quant. | Band % | Lane % |
|---|---|---|---|---|---|---|---|---|---|
| 1 | | N/A | 0.469 | 1,937,936 | 2,190,608 | N/A | N/A | 100.0 | 99.4 |

| | |
|---|---|
| Lane Background | Lane background subtracted with disk size: 9.4 |
| Lane Width | 7.48 mm |

### Lane 10 - G5

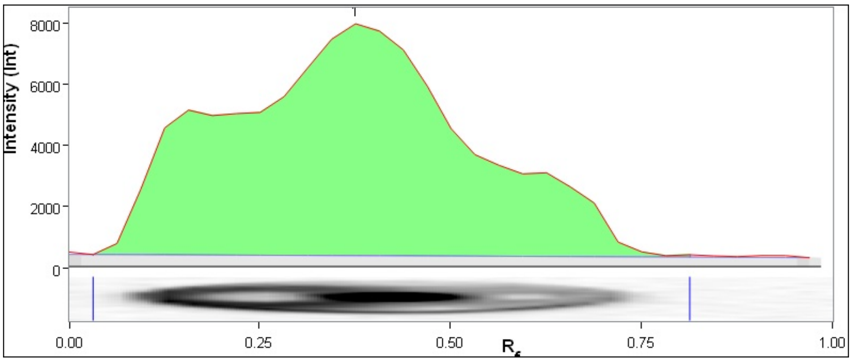


| Band No. | Band Label | Mol. Wt. (KDa) | Relative Front | Adj. Volume (Int) | Volume (Int) | Abs. Quant. | Rel. Quant. | Band % | Lane % |
|---|---|---|---|---|---|---|---|---|---|
| 1 | | N/A | 0.406 | 3,221,456 | 3,528,420 | N/A | N/A | 100.0 | 99.7 |

| | |
|---|---|
| Lane Background | Lane background subtracted with disk size: 9.4 |
| Lane Width | 7.48 mm |

### Lane 11 - Pull_Youngs

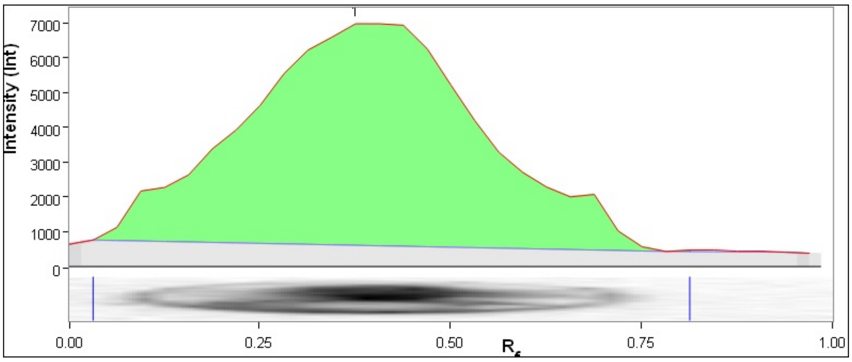


| Band No. | Band Label | Mol. Wt. (KDa) | Relative Front | Adj. Volume (Int) | Volume (Int) | Abs. Quant. | Rel. Quant. | Band % | Lane % |
|---|---|---|---|---|---|---|---|---|---|
| 1 | | N/A | 0.406 | 2,353,428 | 2,808,624 | N/A | N/A | 100.0 | 99.9 |

| | |
|---|---|
| Lane Background | Lane background subtracted with disk size: 9.4 |
| Lane Width | 7.48 mm |

### Lane 12 - Pull_Geriatrics

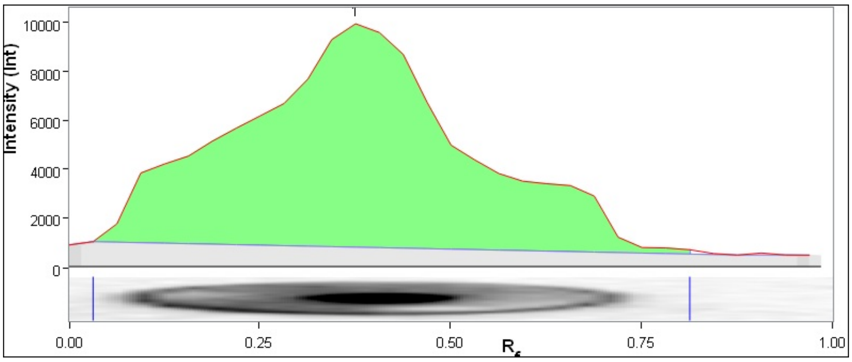


| Band No. | Band Label | Mol. Wt. (KDa) | Relative Front | Adj. Volume (Int) | Volume (Int) | Abs. Quant. | Rel. Quant. | Band % | Lane % |
|---|---|---|---|---|---|---|---|---|---|
| 1 | | N/A | 0.406 | 3,291,820 | 3,907,008 | N/A | N/A | 100.0 | 99.9 |

| | |
|---|---|
| Lane Background | Lane background subtracted with disk size: 9.4 |
| Lane Width | 7.48 mm |
