# Supplementary material for: Aging and diet alter the protein ubiquitylation landscape in the mouse brain
Source: Nat Commun. 2025 Jun 6;16:5266. doi: 10.1038/s41467-025-60542-6 (PMC12144301; doi:10.1038/s41467-025-60542-6)
Supplement: Supplementary file 13 — Source Data [file 41467_2025_60542_MOESM13_ESM.zip › Source_data/Figure_S2/C/Ponceau_Report_Ponceau_research_ori 2021-03-18_15h30m37s.pdf]

Image Report: Ponceau_research_ori 2021-03-18_15h30m37s

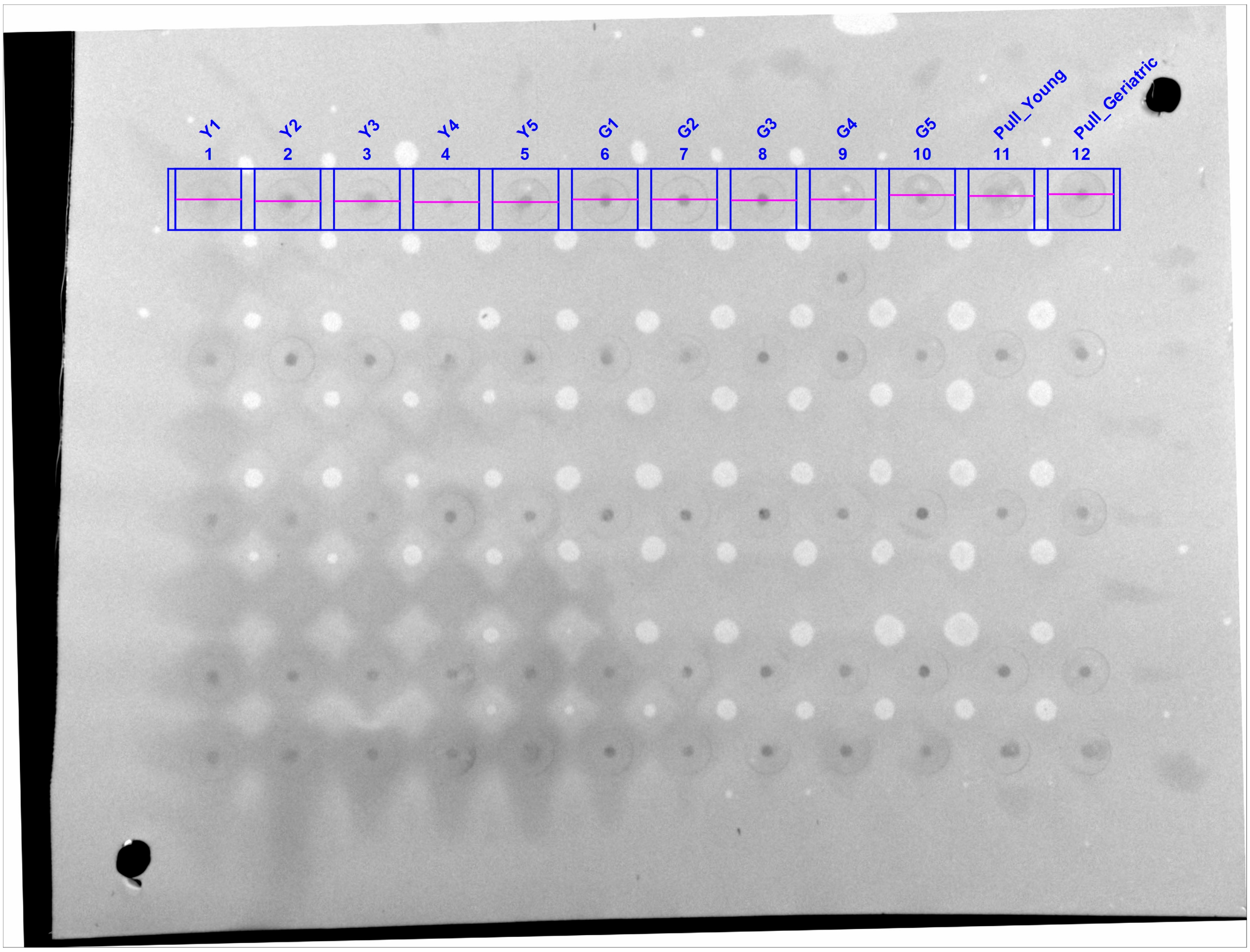

S:\Ori\Antonio\DotBlot\180321_Brains_Mice_FK2_K63_K48_K27_ab19247\Ponceau_research_ori 2021-03-18_15h30m37s.scn

Acquisition Information

Imager	ChemiDoc XRS+
Exposure Time (sec)	0.100 (Auto - Intense Bands)
Flat Field	Applied (Lens)
Serial Number	721BR12704
Software Version	6.0.1.34
Application	Colorimetric
Excitation Source	White Epi Illumination
Emission Filter	No Filter

Image Information

Acquisition Date	3/18/2021 3:31:07 PM
User Name	research_ori
Image Area (mm)	X: 142.4 Y: 107.8
Pixel Size (µm)	X: 100.6 Y: 100.6

Data Range (Int)	0 - 64325

Analysis Settings

Detection	Lane detection:<br>Manually created lanes<br><br>Band detection:<br><br>Manually adjusted bands<br><br>Lane Background Subtraction:<br>Lane background subtracted with disk size: 24.4<br><br>Lane width: 7.54 mm

Lane Statistics

Lane No.	Adj. Total Band Vol. (Int)	Total Band Vol. (Int)	Adj. Total Lane Vol. (Int)	Total Lane Vol. (Int)	Bkgd. Vol. (Int)	Norm. Factor
1	5,569,650	75,142,950	5,898,450	84,448,950	78,550,500	N/A
2	4,403,850	73,028,850	4,508,400	84,570,900	80,062,500	N/A
3	4,141,425	70,441,200	4,304,250	82,965,000	78,660,750	N/A
4	2,175,900	70,524,450	2,362,875	83,454,375	81,091,500	N/A
5	5,002,650	78,342,450	5,094,225	87,897,225	82,803,000	N/A
6	4,034,100	75,243,600	4,158,000	90,100,500	85,942,500	N/A
7	3,181,350	76,553,850	3,308,325	88,909,575	85,601,250	N/A
8	5,067,375	75,929,325	5,372,400	89,445,900	84,073,500	N/A
9	3,334,650	72,569,250	3,611,100	87,170,100	83,559,000	N/A
10	3,686,325	72,246,675	3,830,475	86,575,725	82,745,250	N/A
11	6,372,825	74,883,450	6,539,250	85,158,000	78,618,750	N/A
12	4,716,825	73,090,200	4,840,725	83,301,975	78,461,250	N/A

Lane And Band Analysis

Lane 1 - Y1

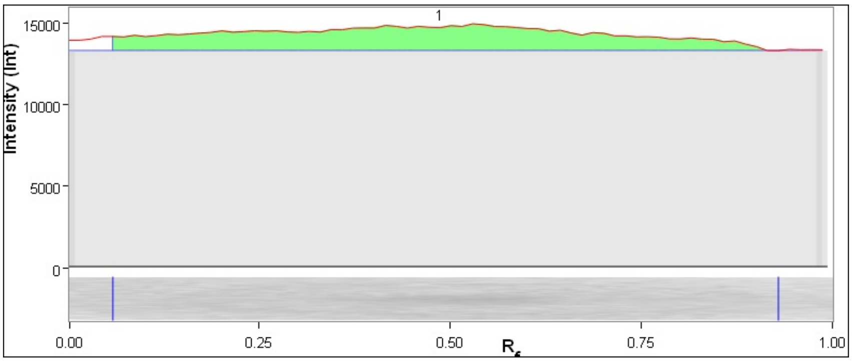

Band No.	Band Label	Mol. Wt. (KDa)	Relative Front	Adj. Volume (Int)	Volume (Int)	Abs. Quant.	Rel. Quant.	Band %	Lane %
1		N/A	0.500	5,569,650	75,142,950	N/A	N/A	100.0	94.4

Lane Background	Lane background subtracted with disk size: 24.4
Lane Width	7.54 mm

Lane 2 - Y2

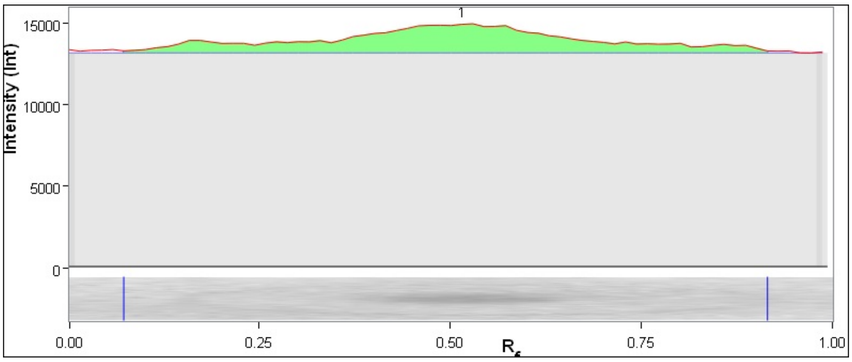

Band No.	Band Label	Mol. Wt. (KDa)	Relative Front	Adj. Volume (Int)	Volume (Int)	Abs. Quant.	Rel. Quant.	Band %	Lane %
1		N/A	0.529	4,403,850	73,028,850	N/A	N/A	100.0	97.7

Lane Background	Lane background subtracted with disk size: 24.4
Lane Width	7.54 mm

Lane 3 - Y3

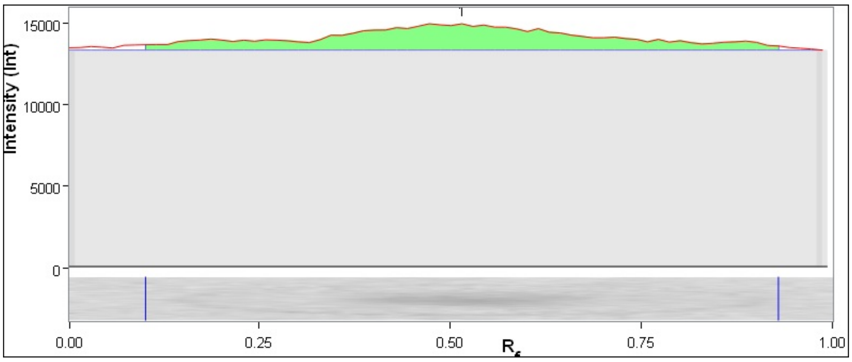

Band No.	Band Label	Mol. Wt. (KDa)	Relative Front	Adj. Volume (Int)	Volume (Int)	Abs. Quant.	Rel. Quant.	Band %	Lane %
1		N/A	0.529	4,141,425	70,441,200	N/A	N/A	100.0	96.2

Lane Background	Lane background subtracted with disk size: 24.4
Lane Width	7.54 mm

Lane 4 - Y4

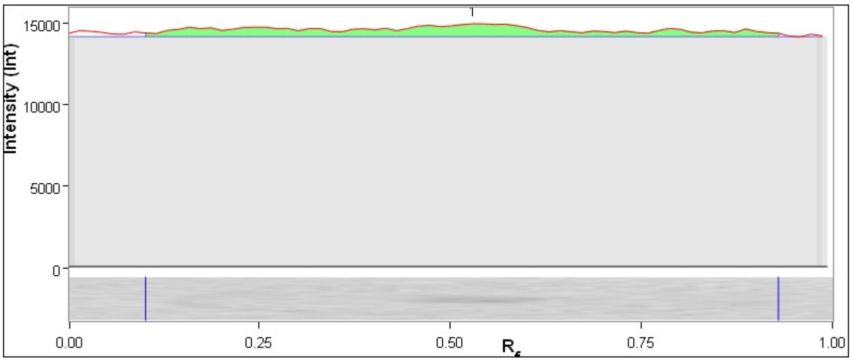

Band No.	Band Label	Mol. Wt. (KDa)	Relative Front	Adj. Volume (Int)	Volume (Int)	Abs. Quant.	Rel. Quant.	Band %	Lane %
1		N/A	0.543	2,175,900	70,524,450	N/A	N/A	100.0	92.1

Lane Background	Lane background subtracted with disk size: 24.4
Lane Width	7.54 mm

Lane 5 - Y5

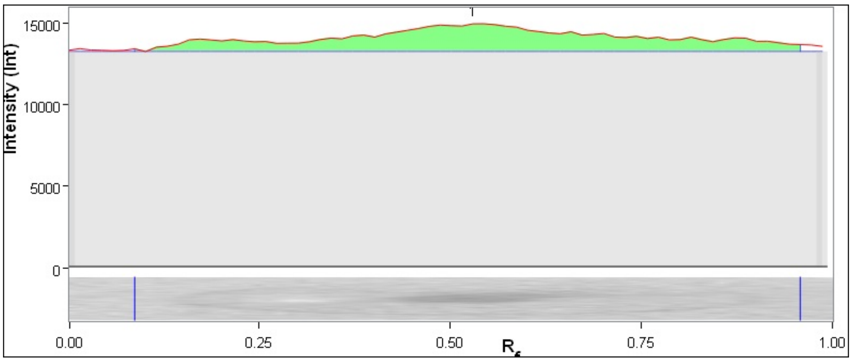

Band No.	Band Label	Mol. Wt. (KDa)	Relative Front	Adj. Volume (Int)	Volume (Int)	Abs. Quant.	Rel. Quant.	Band %	Lane %
1		N/A	0.543	5,002,650	78,342,450	N/A	N/A	100.0	98.2

Lane Background	Lane background subtracted with disk size: 24.4
Lane Width	7.54 mm

Lane 6 - G1

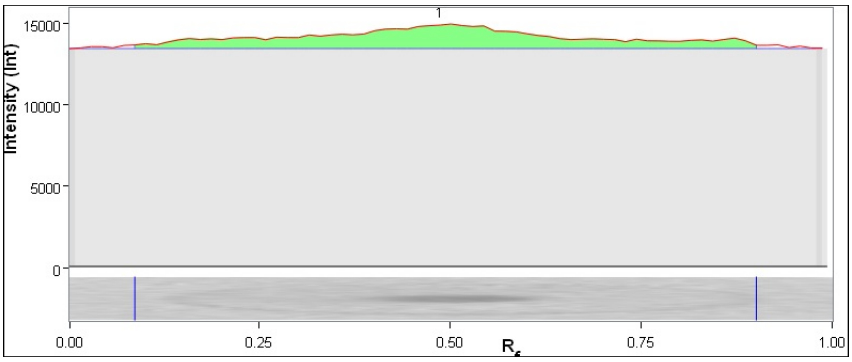

Band No.	Band Label	Mol. Wt. (KDa)	Relative Front	Adj. Volume (Int)	Volume (Int)	Abs. Quant.	Rel. Quant.	Band %	Lane %
1		N/A	0.500	4,034,100	75,243,600	N/A	N/A	100.0	97.0

Lane Background	Lane background subtracted with disk size: 24.4
Lane Width	7.54 mm

Lane 7 - G2

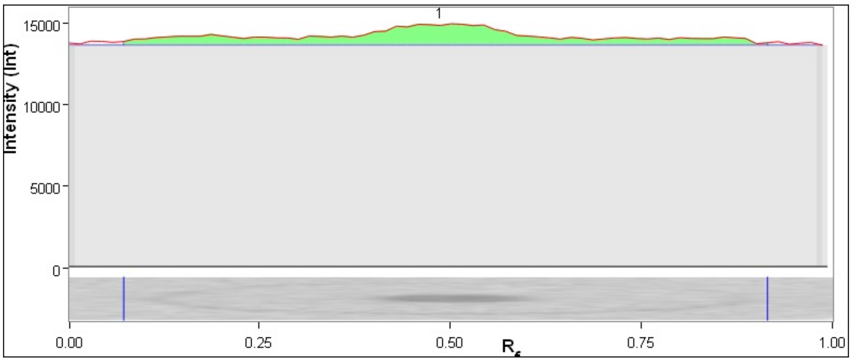

Band No.	Band Label	Mol. Wt. (KDa)	Relative Front	Adj. Volume (Int)	Volume (Int)	Abs. Quant.	Rel. Quant.	Band %	Lane %
1		N/A	0.500	3,181,350	76,553,850	N/A	N/A	100.0	96.2

Lane Background	Lane background subtracted with disk size: 24.4
Lane Width	7.54 mm

Lane 8 - G3

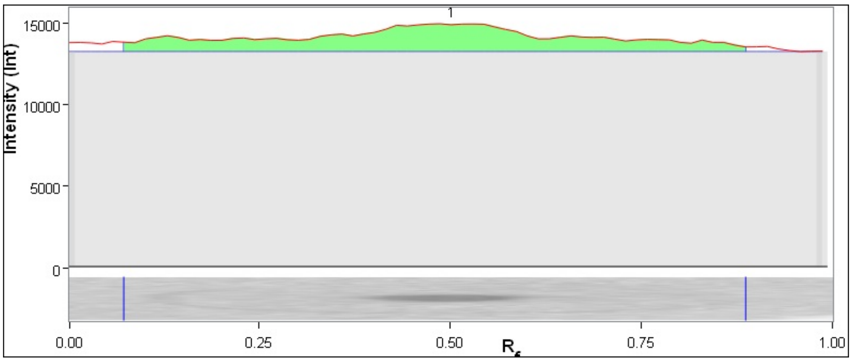

Band No.	Band Label	Mol. Wt. (KDa)	Relative Front	Adj. Volume (Int)	Volume (Int)	Abs. Quant.	Rel. Quant.	Band %	Lane %
1		N/A	0.514	5,067,375	75,929,325	N/A	N/A	100.0	94.3

Lane Background	Lane background subtracted with disk size: 24.4
Lane Width	7.54 mm

Lane 9 - G4

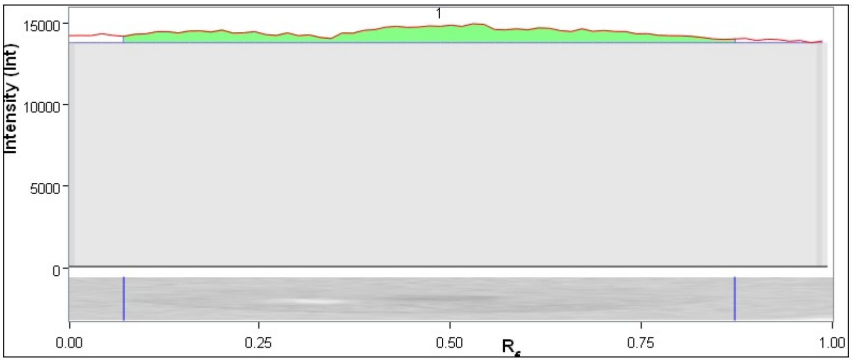

Band No.	Band Label	Mol. Wt. (KDa)	Relative Front	Adj. Volume (Int)	Volume (Int)	Abs. Quant.	Rel. Quant.	Band %	Lane %
1		N/A	0.500	3,334,650	72,569,250	N/A	N/A	100.0	92.3

Lane Background	Lane background subtracted with disk size: 24.4
Lane Width	7.54 mm

Lane 10 - G5

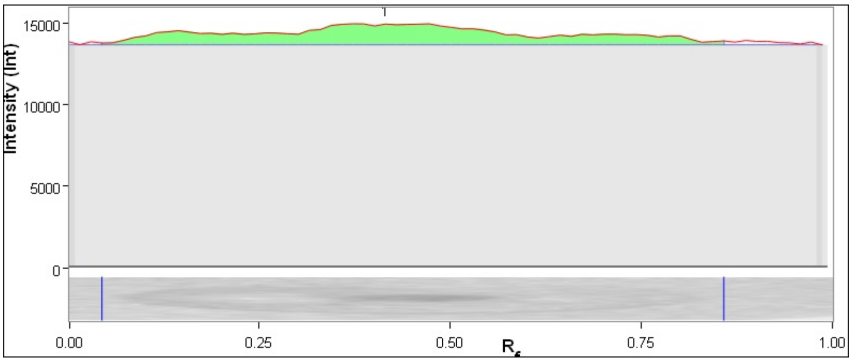

Band No.	Band Label	Mol. Wt. (KDa)	Relative Front	Adj. Volume (Int)	Volume (Int)	Abs. Quant.	Rel. Quant.	Band %	Lane %
1		N/A	0.429	3,686,325	72,246,675	N/A	N/A	100.0	96.2

Lane Background	Lane background subtracted with disk size: 24.4
Lane Width	7.54 mm

Lane 11 - Pull_Young

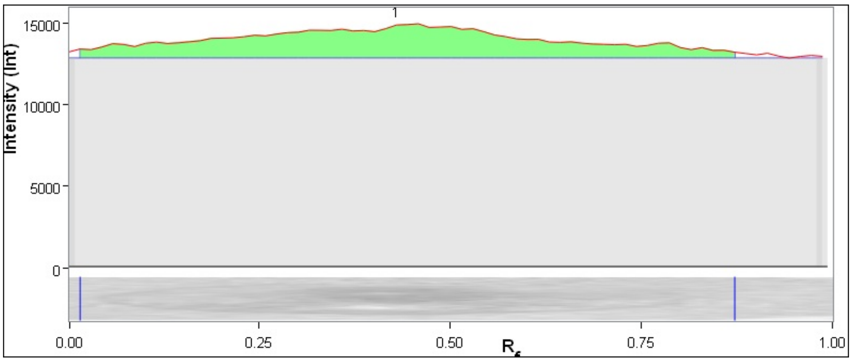

Band No.	Band Label	Mol. Wt. (KDa)	Relative Front	Adj. Volume (Int)	Volume (Int)	Abs. Quant.	Rel. Quant.	Band %	Lane %
1		N/A	0.443	6,372,825	74,883,450	N/A	N/A	100.0	97.5

Lane Background	Lane background subtracted with disk size: 24.4
Lane Width	7.54 mm

Lane 12 - Pull_Geriatric

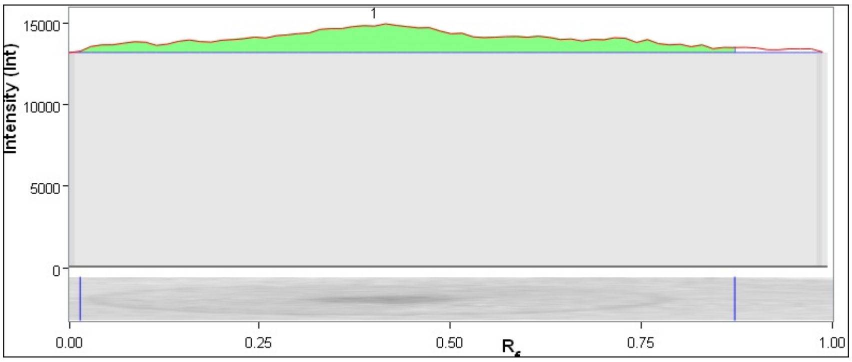

Band No.	Band Label	Mol. Wt. (KDa)	Relative Front	Adj. Volume (Int)	Volume (Int)	Abs. Quant.	Rel. Quant.	Band %	Lane %
1		N/A	0.414	4,716,825	73,090,200	N/A	N/A	100.0	97.4

Lane Background	Lane background subtracted with disk size: 24.4
Lane Width	7.54 mm
