# Supplementary figures and images for: Aging and diet alter the protein ubiquitylation landscape in the mouse brain
Source: Nat Commun. 2025 Jun 6;16:5266. doi: 10.1038/s41467-025-60542-6 (PMC12144301; doi:10.1038/s41467-025-60542-6)

# Correlation Ubiquit. Brain / Liver Mouse

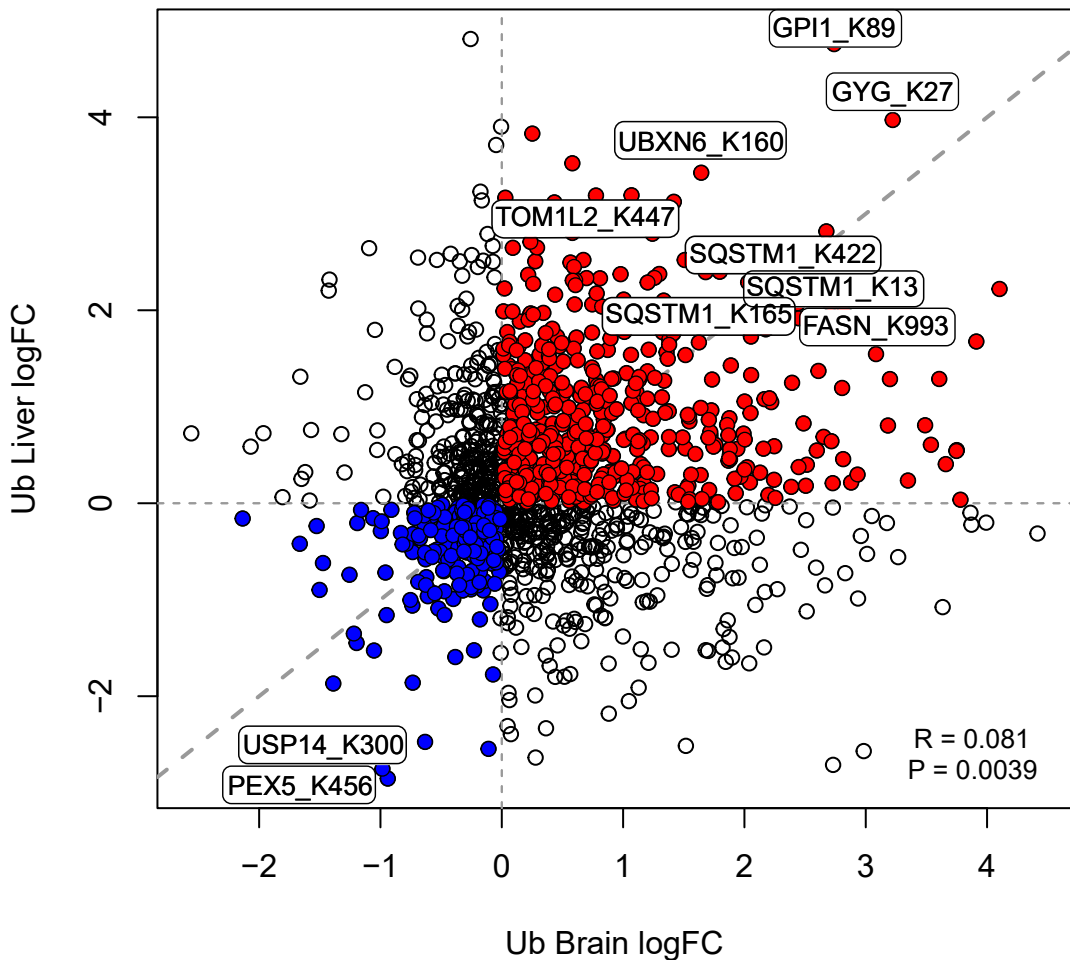

Supplement: Supplementary file 13 — Source Data [file 41467_2025_60542_MOESM13_ESM.zip › Source_data/Figure_S4/G/G.pdf]

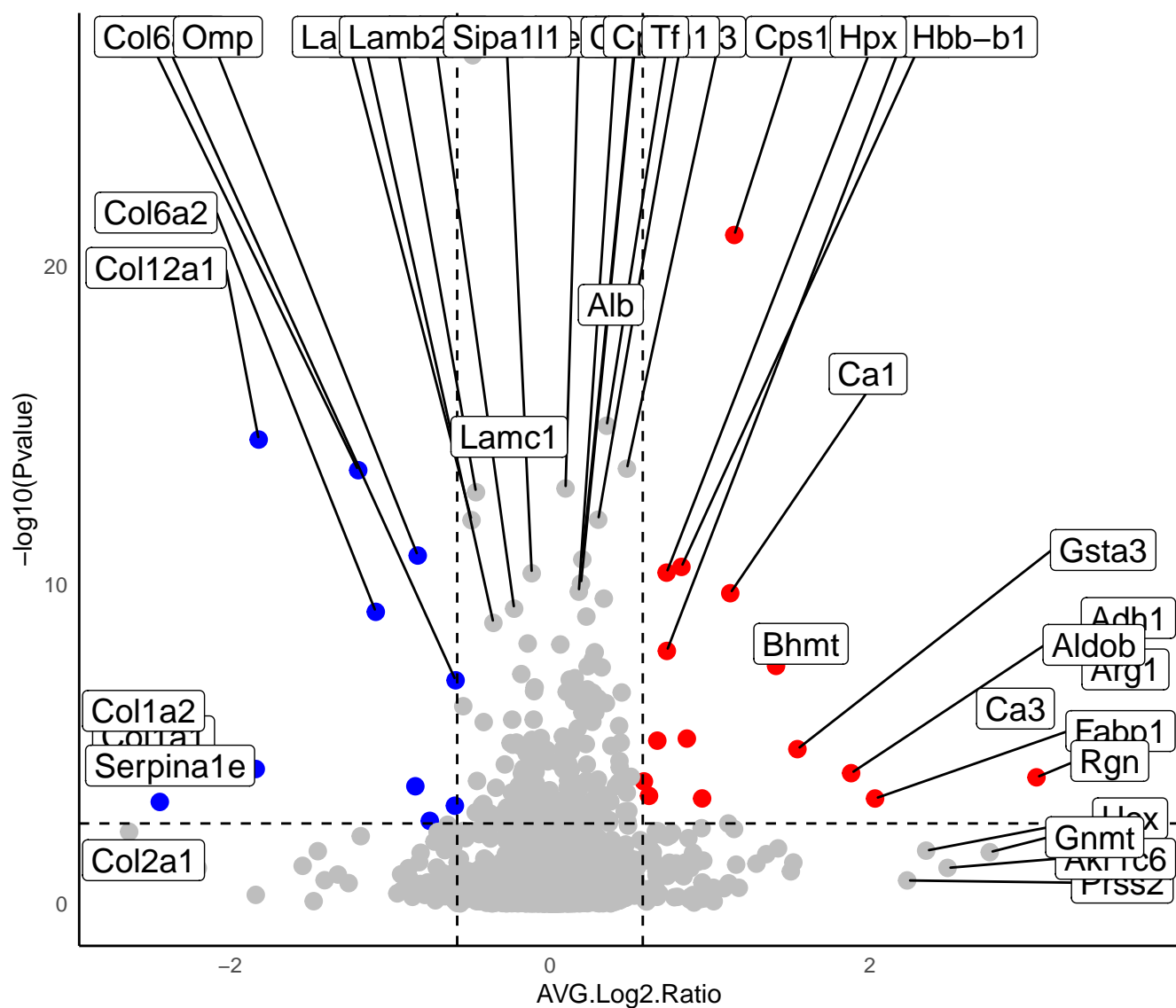

Supplement: Supplementary file 13 — Source Data [file 41467_2025_60542_MOESM13_ESM.zip › Source_data/Figure_5/D/D_Left.pdf]

# Reverted-Up

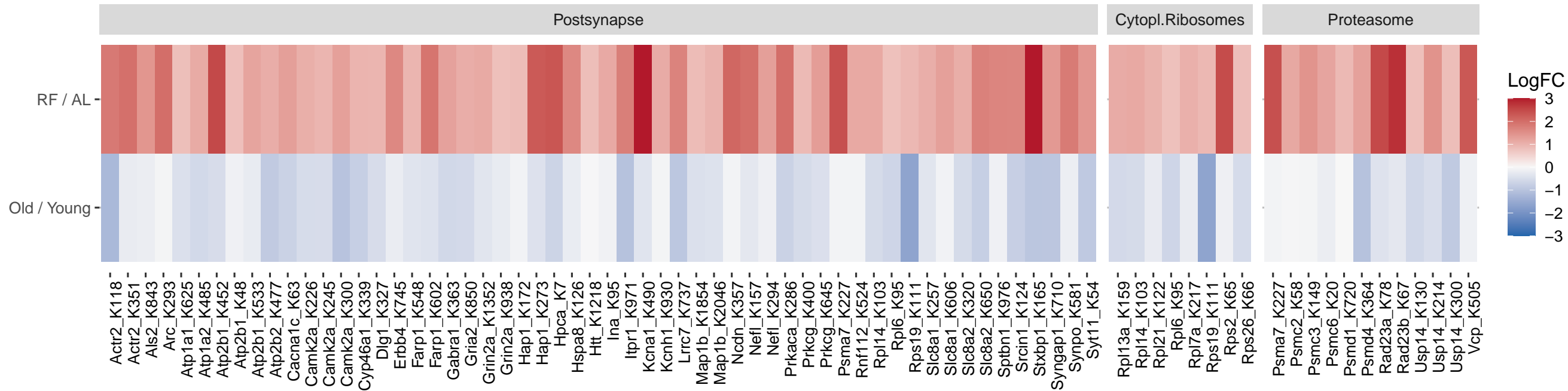

Supplement: Supplementary file 13 — Source Data [file 41467_2025_60542_MOESM13_ESM.zip › Source_data/Figure_5/H/H.pdf]

## % affected - Re-feeding vs Old

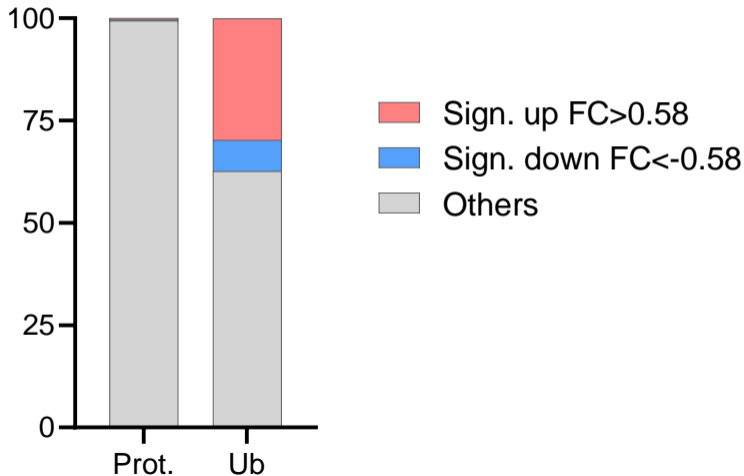

Supplement: Supplementary file 13 — Source Data [file 41467_2025_60542_MOESM13_ESM.zip › Source_data/Figure_5/C/C.pdf]

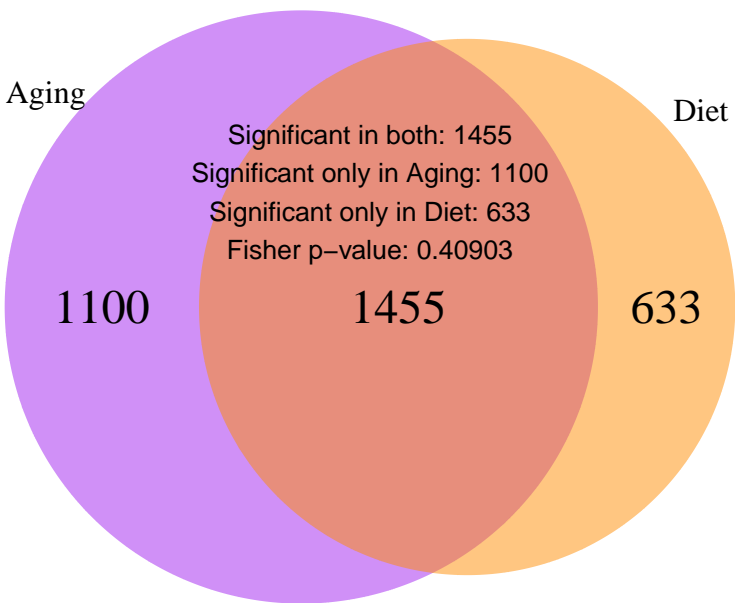

Supplement: Supplementary file 13 — Source Data [file 41467_2025_60542_MOESM13_ESM.zip › Source_data/Figure_5/E/E.pdf]

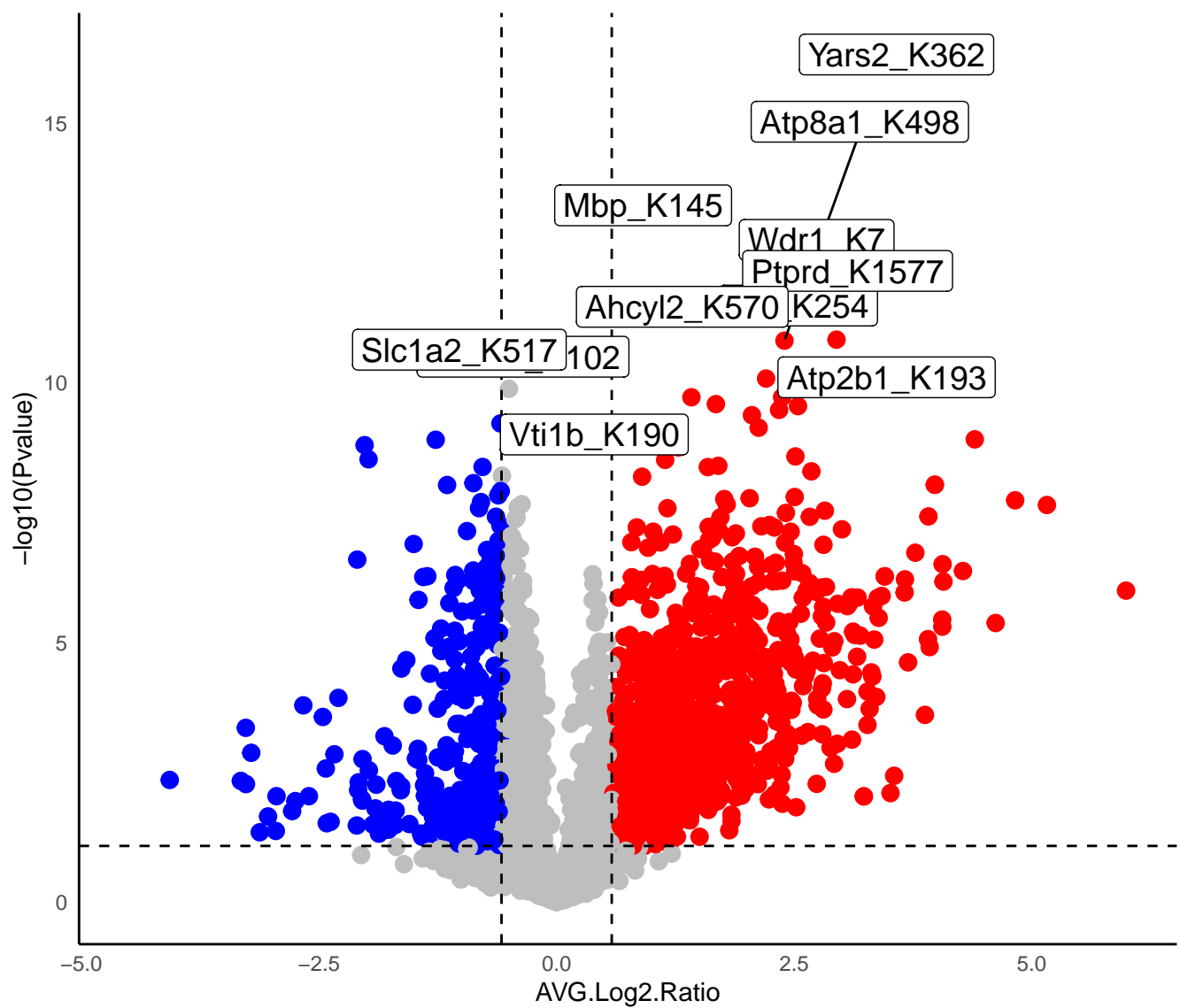

Supplement: Supplementary file 13 — Source Data [file 41467_2025_60542_MOESM13_ESM.zip › Source_data/Figure_5/D/D_Right.pdf]

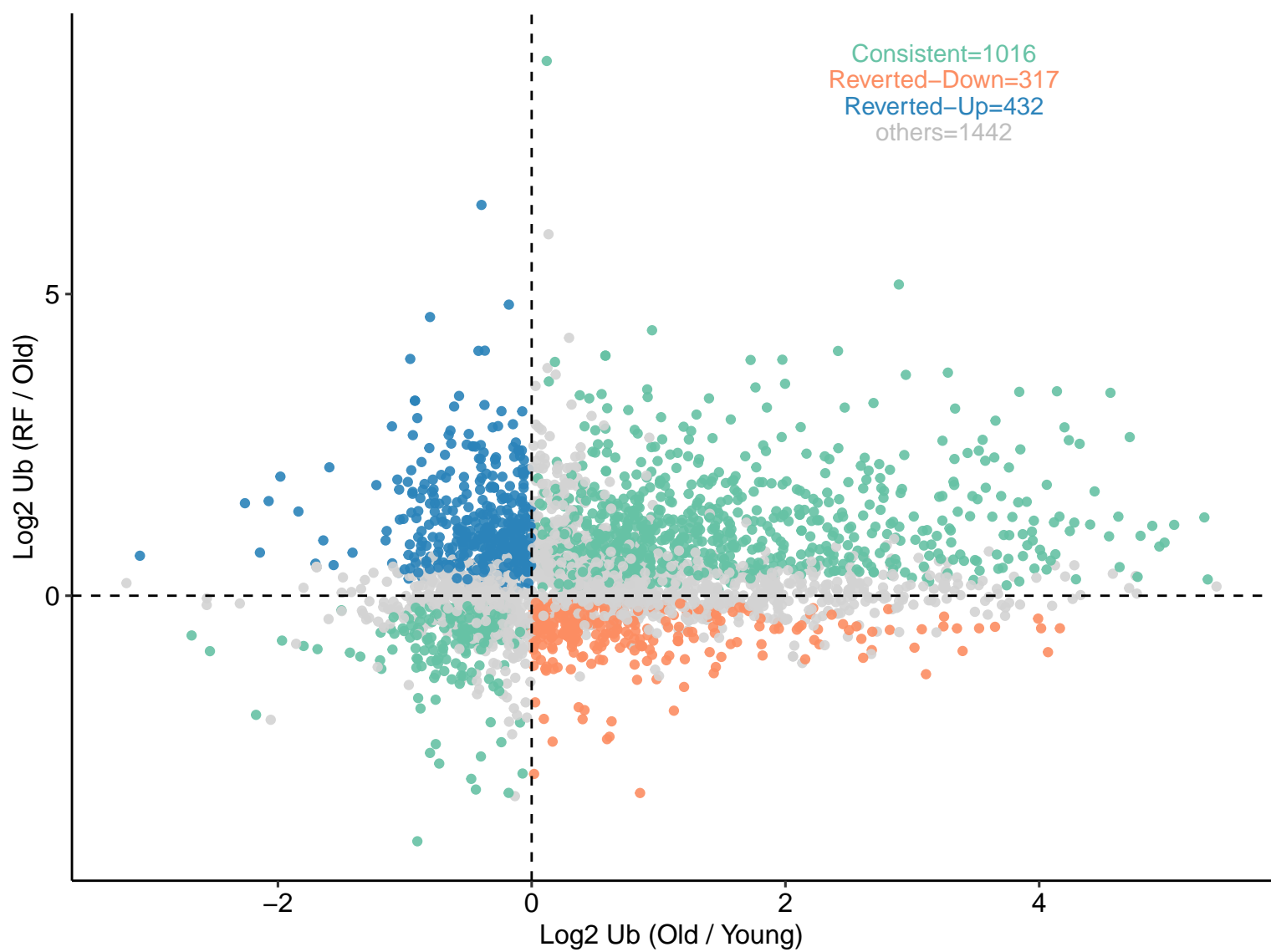

Supplement: Supplementary file 13 — Source Data [file 41467_2025_60542_MOESM13_ESM.zip › Source_data/Figure_5/F/F.pdf]

# Gene Ontology Enrichment – Cellular Comp

Ub enrichment – OvsY – P.adj.  $\leq 0.05$

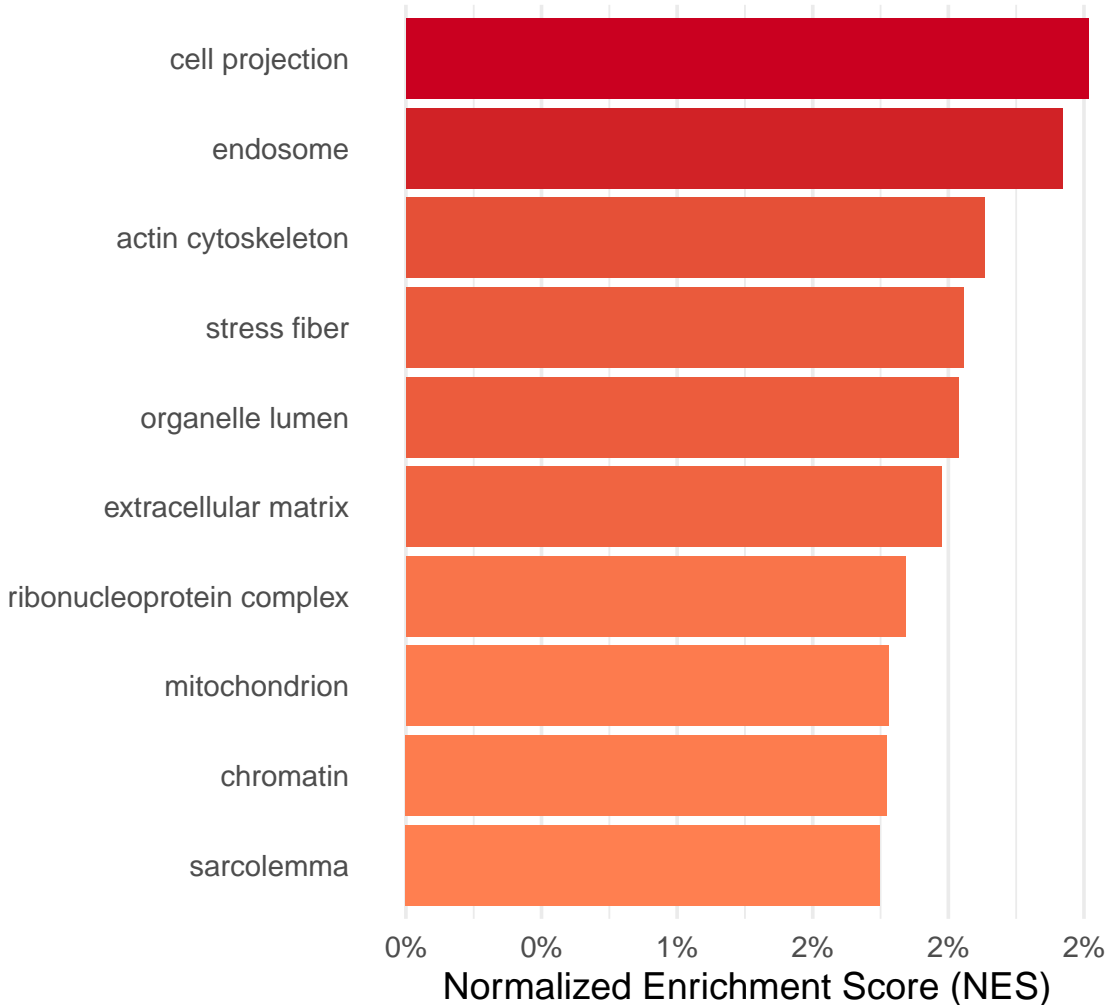

Supplement: Supplementary file 13 — Source Data [file 41467_2025_60542_MOESM13_ESM.zip › Source_data/Figure_S4/E/E.pdf]

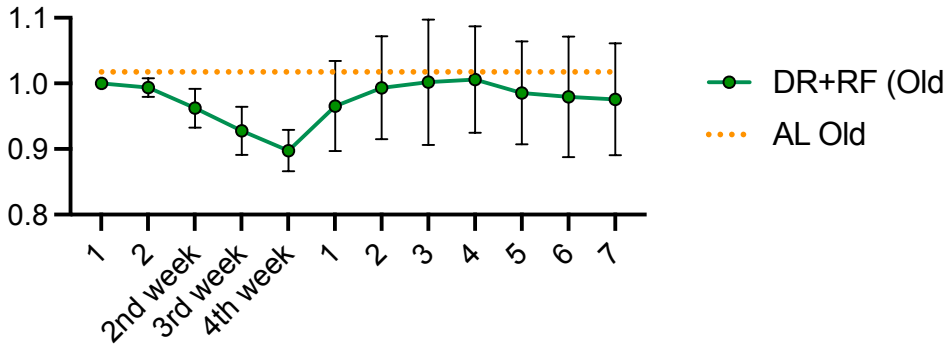

Supplement: Supplementary file 13 — Source Data [file 41467_2025_60542_MOESM13_ESM.zip › Source_data/Figure_5/A/A.pdf]

# Individuals – PCA

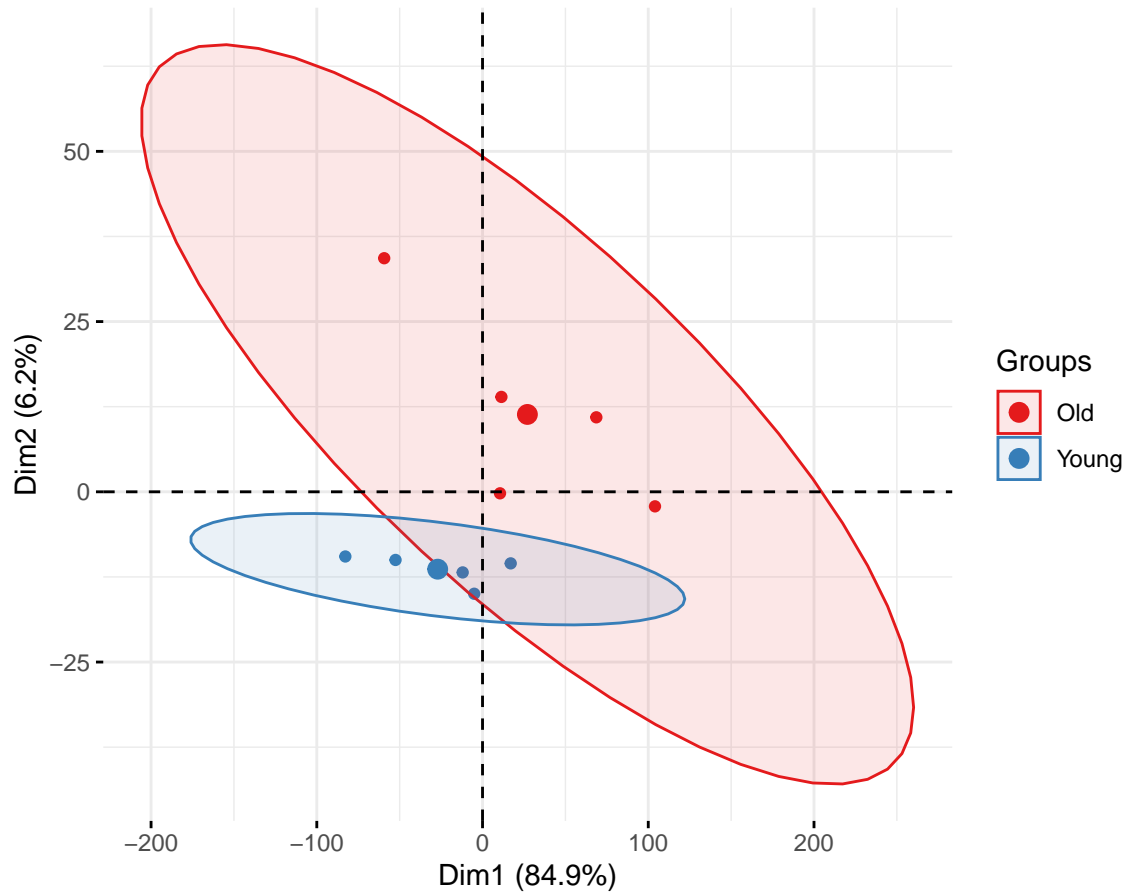

Supplement: Supplementary file 13 — Source Data [file 41467_2025_60542_MOESM13_ESM.zip › Source_data/Figure_S4/B/B.pdf]

# Ubiquitylated sites - Reverted - down - Q <0.05

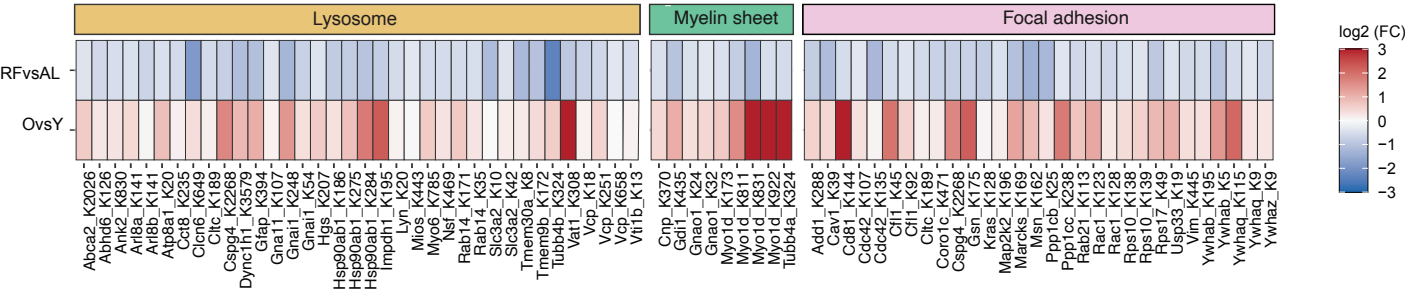

Supplement: Supplementary file 13 — Source Data [file 41467_2025_60542_MOESM13_ESM.zip › Source_data/Figure_S9/E/E.pdf]

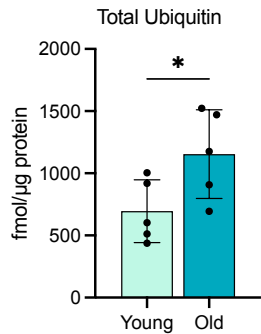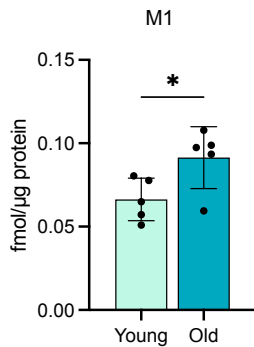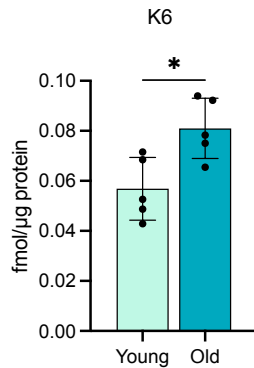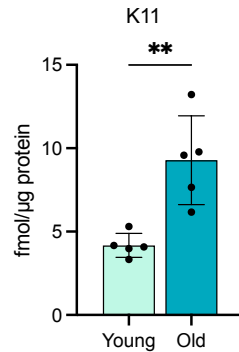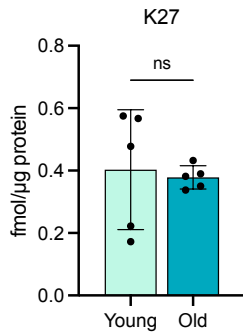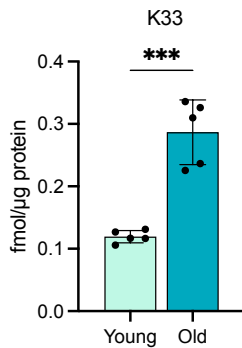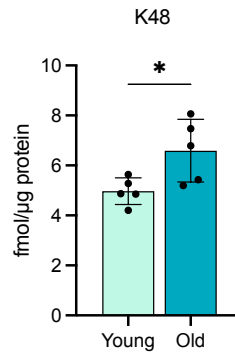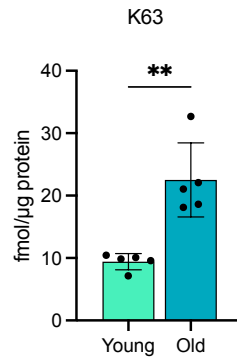

Supplement: Supplementary file 13 — Source Data [file 41467_2025_60542_MOESM13_ESM.zip › Source_data/Figure_4/C/C_mouse_data.pdf]

## Correlation PTM vs Whole Proteome Fold Changes

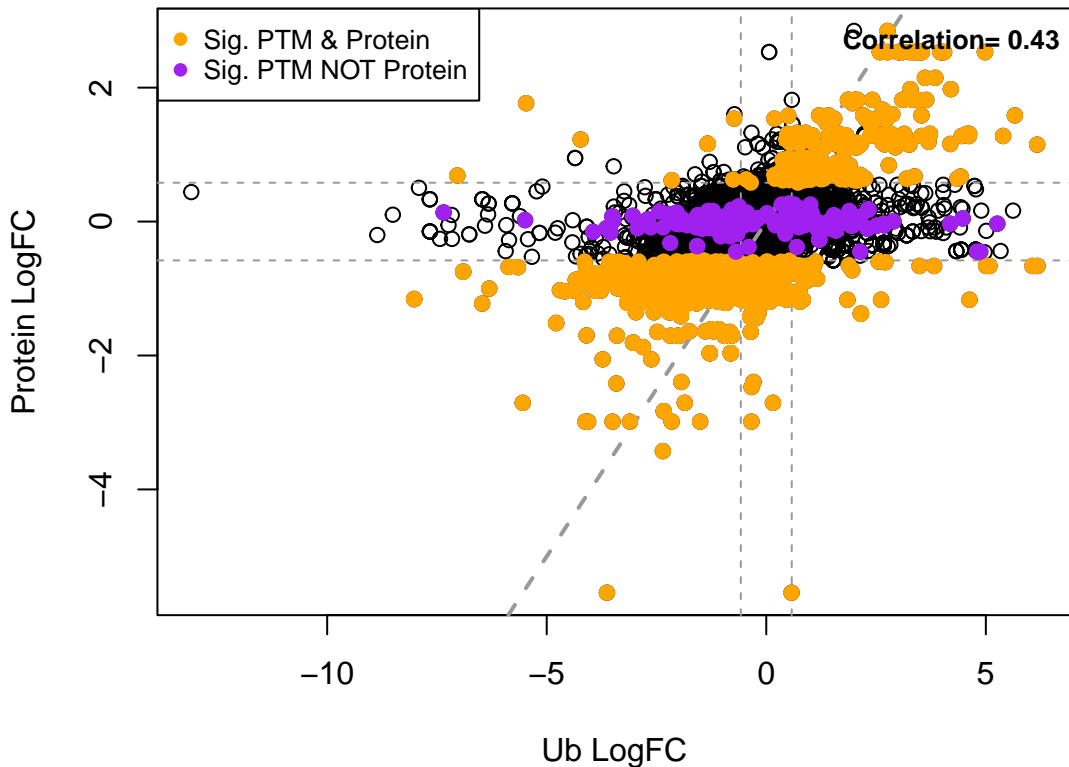

Supplement: Supplementary file 13 — Source Data [file 41467_2025_60542_MOESM13_ESM.zip › Source_data/Figure_S5/A/A.pdf]

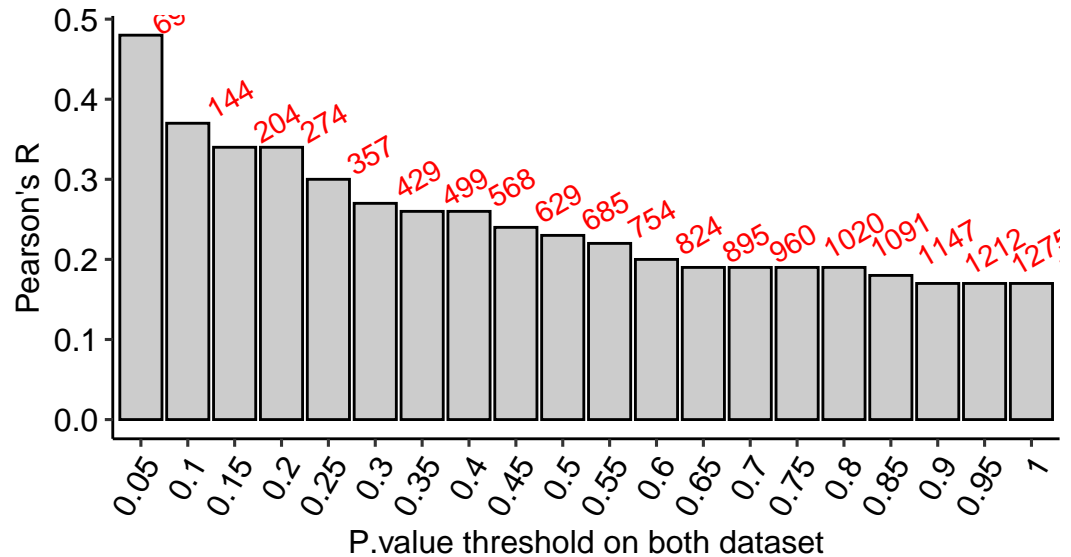

Supplement: Supplementary file 13 — Source Data [file 41467_2025_60542_MOESM13_ESM.zip › Source_data/Figure_S5/B/B.pdf]

# Gene Ontology Enrichment – Cellular Comp

Ub enrichment – RFvsAL – P.adj.  $\leq 0.05$

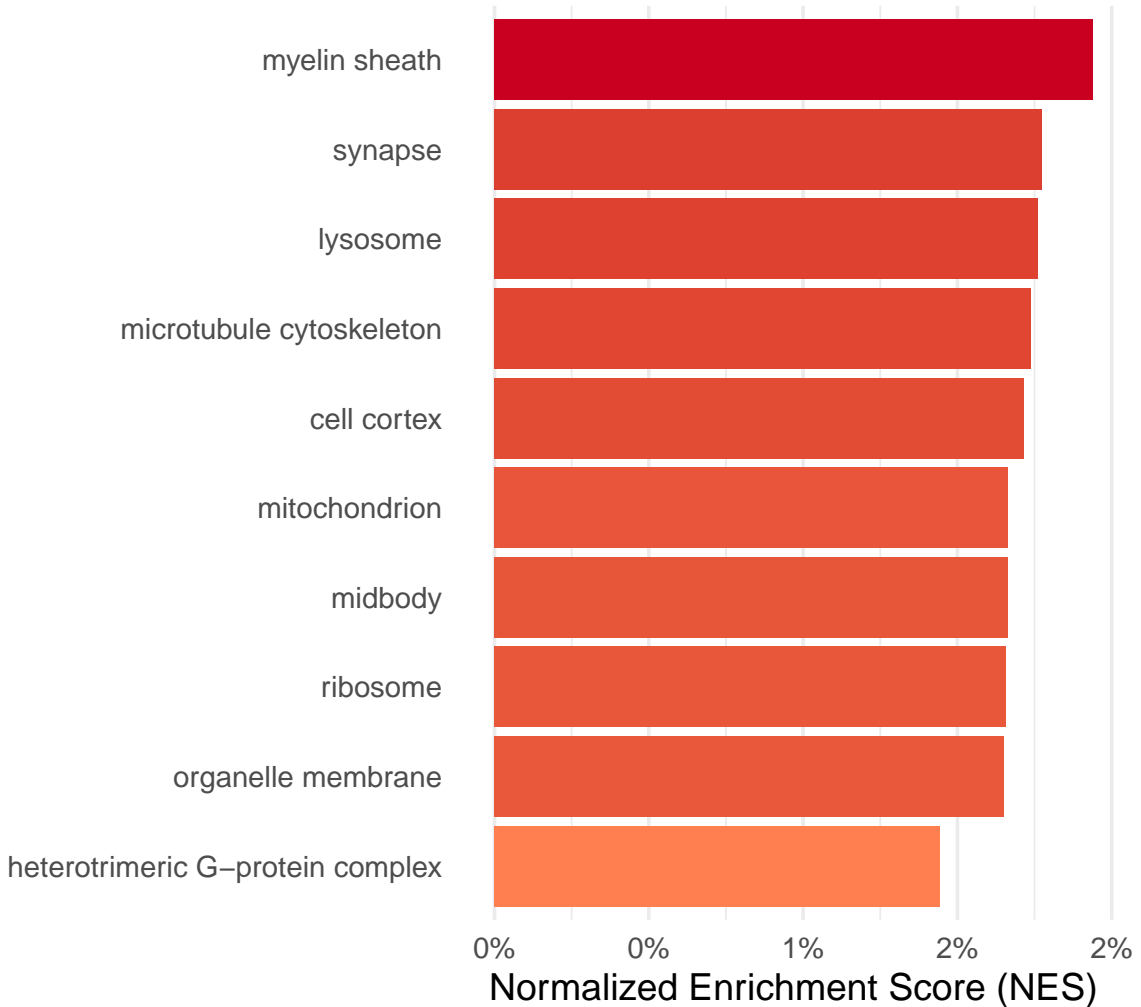

Supplement: Supplementary file 13 — Source Data [file 41467_2025_60542_MOESM13_ESM.zip › Source_data/Figure_S9/B/B.pdf]

## AQUA-PRM - Brain Mouse Aging

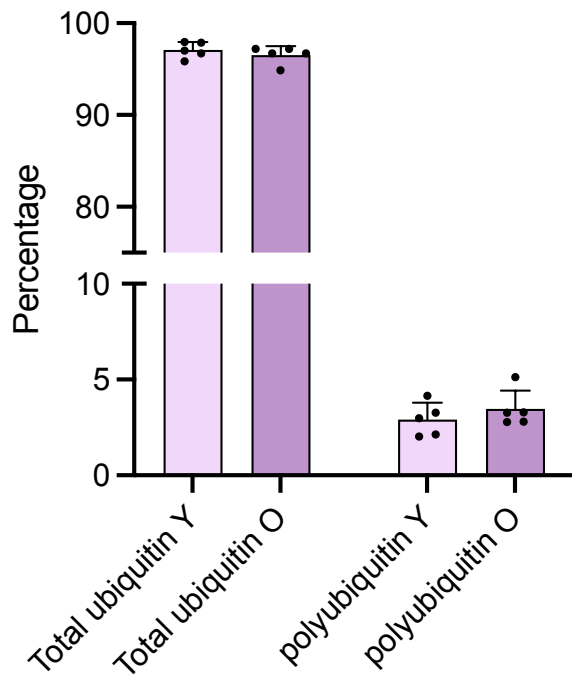

## AQUA-PRM iNeurons treatments

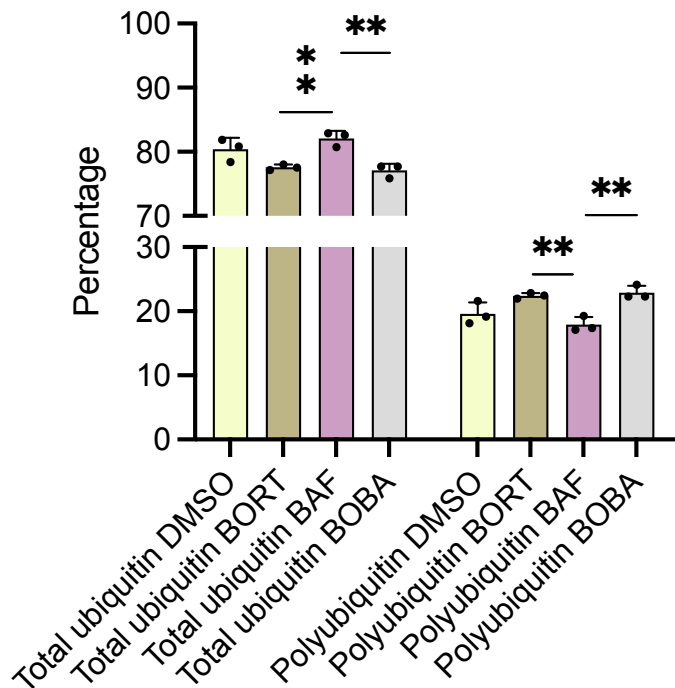

Supplement: Supplementary file 13 — Source Data [file 41467_2025_60542_MOESM13_ESM.zip › Source_data/Figure_4/B/B.pdf]

# Individuals – PCA

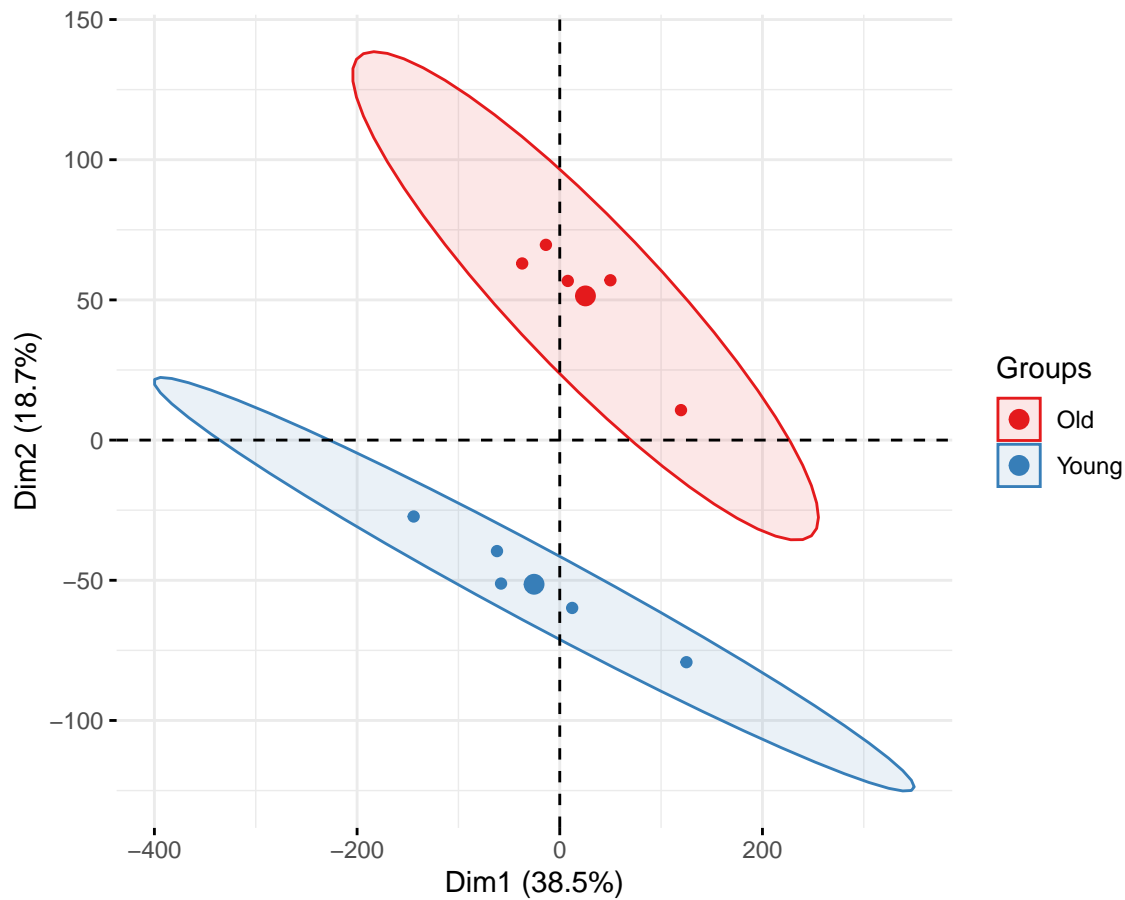

Supplement: Supplementary file 13 — Source Data [file 41467_2025_60542_MOESM13_ESM.zip › Source_data/Figure_S3/D/D.pdf]

## Consistent-Up

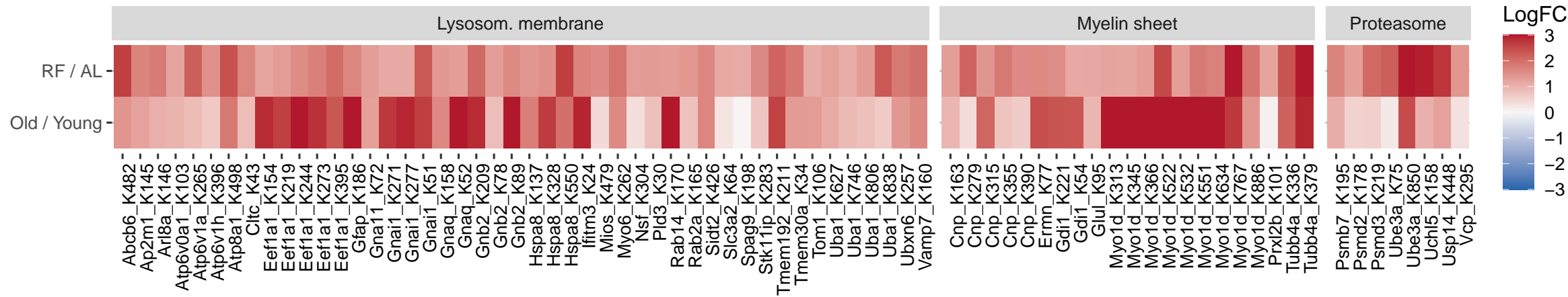

## Consistent-Up

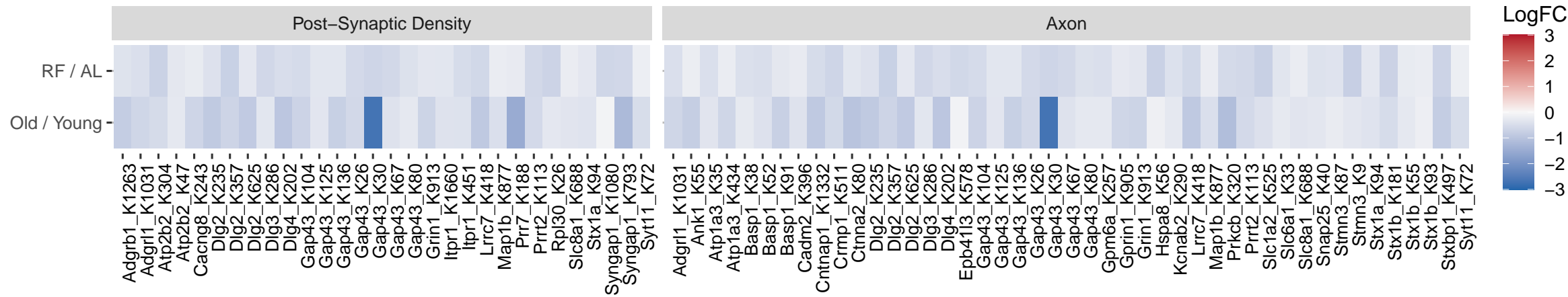

Supplement: Supplementary file 13 — Source Data [file 41467_2025_60542_MOESM13_ESM.zip › Source_data/Figure_S9/D/D.pdf]

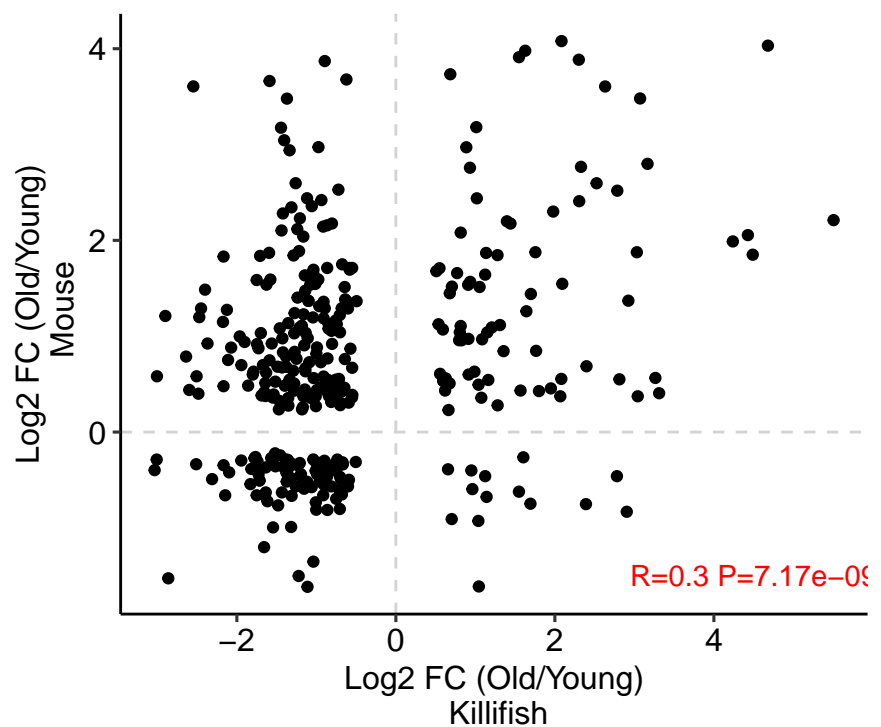

Supplement: Supplementary file 13 — Source Data [file 41467_2025_60542_MOESM13_ESM.zip › Source_data/Figure_S5/C/C.pdf]

# Correlation PTM vs Whole Proteome Fold Changes – Liver

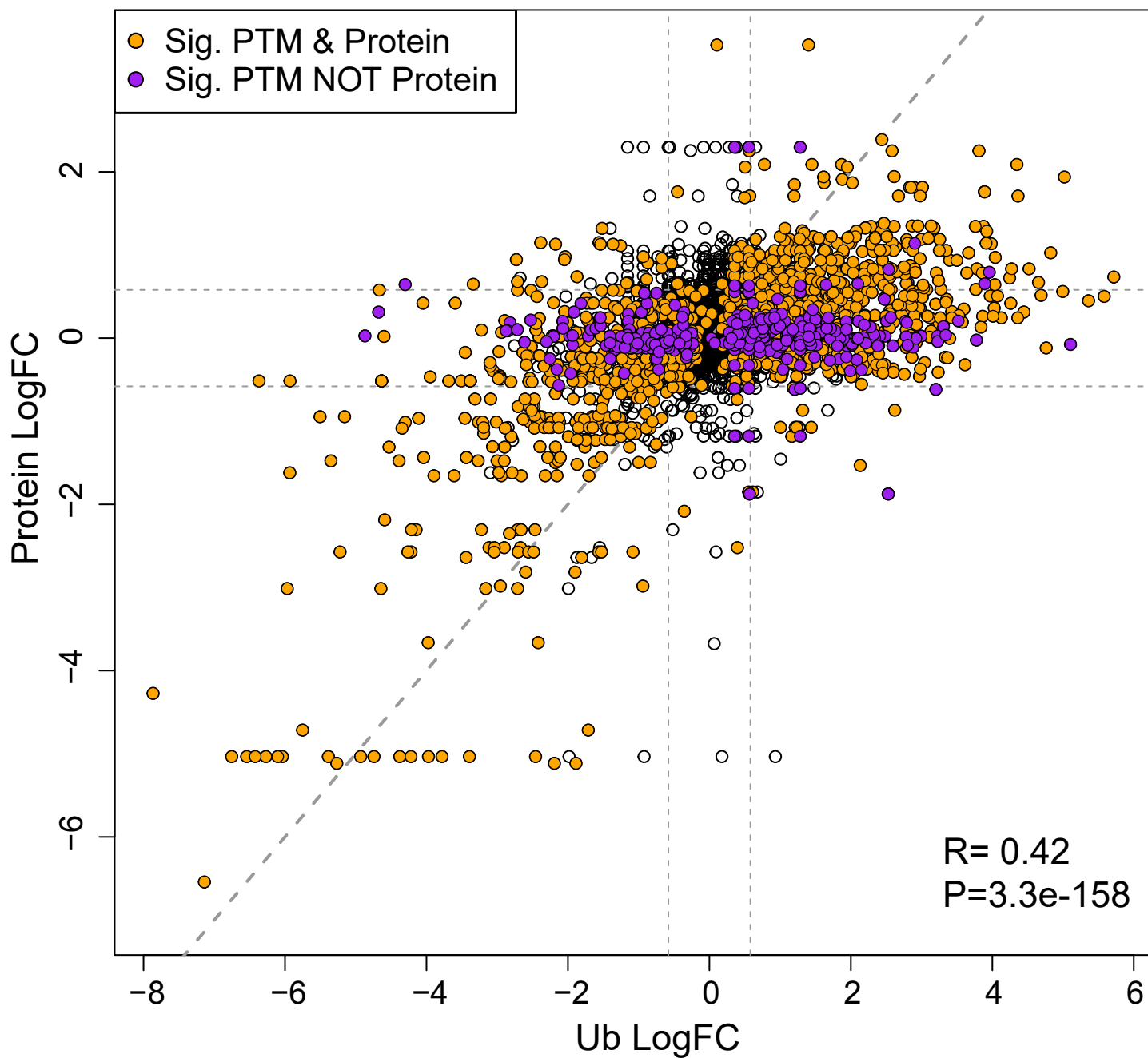

Supplement: Supplementary file 13 — Source Data [file 41467_2025_60542_MOESM13_ESM.zip › Source_data/Figure_S4/F/F.pdf]

# Liver ubiquitylation enrichment - OvsY

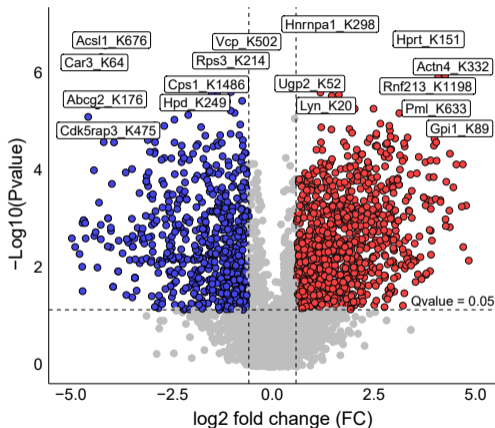

Supplement: Supplementary file 13 — Source Data [file 41467_2025_60542_MOESM13_ESM.zip › Source_data/Figure_S4/D/D.pdf]

Individuals – PCA

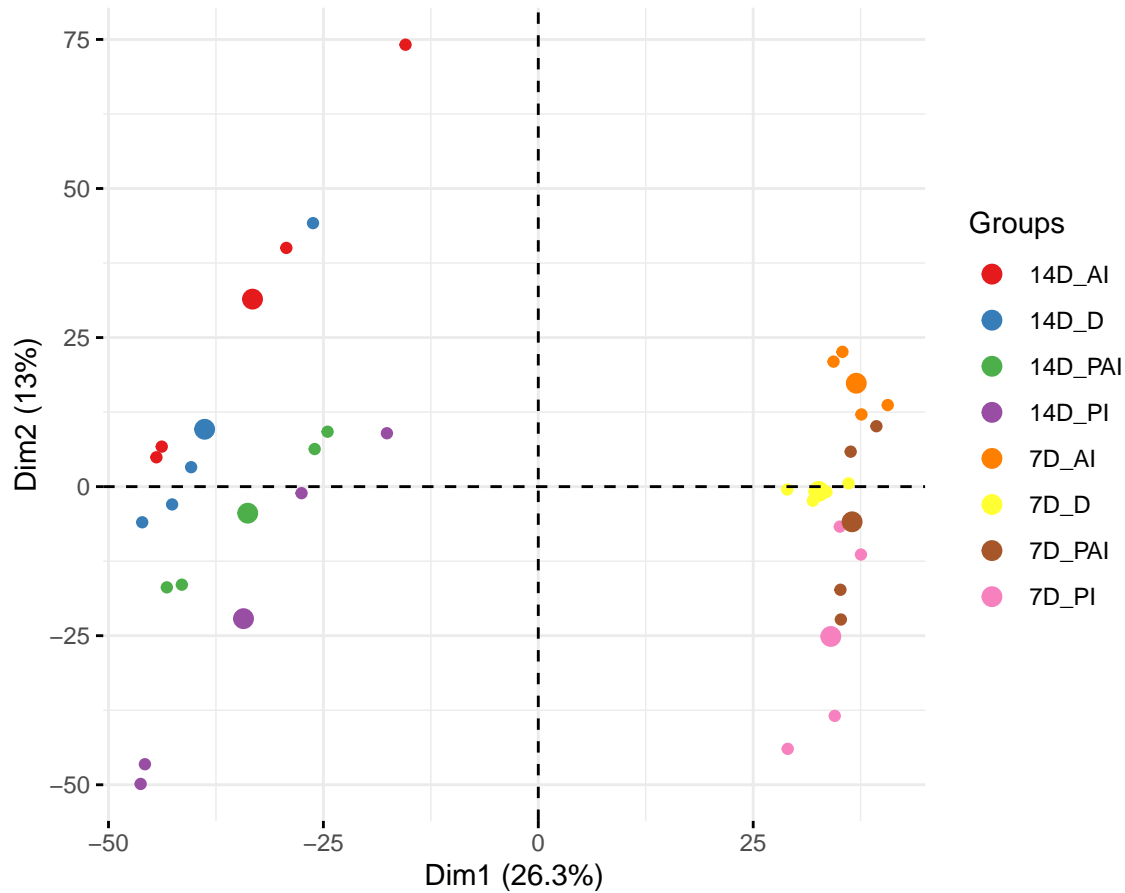

Supplement: Supplementary file 13 — Source Data [file 41467_2025_60542_MOESM13_ESM.zip › Source_data/Figure_3/B/B.pdf]

# Individuals – PCA

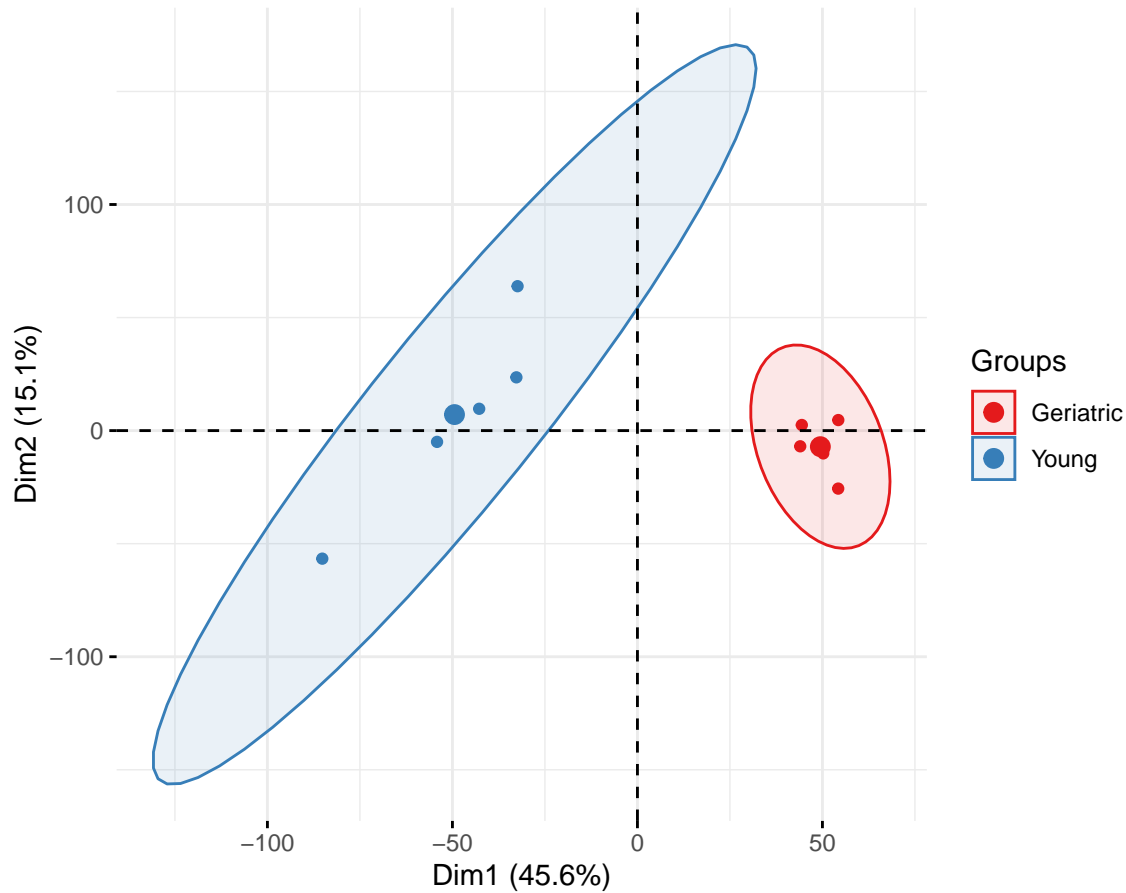

Supplement: Supplementary file 13 — Source Data [file 41467_2025_60542_MOESM13_ESM.zip › Source_data/Figure_S3/A/A.pdf]

# Gene Ontology Enrichment – Biological

14DaysAI / 14DaysDMSO

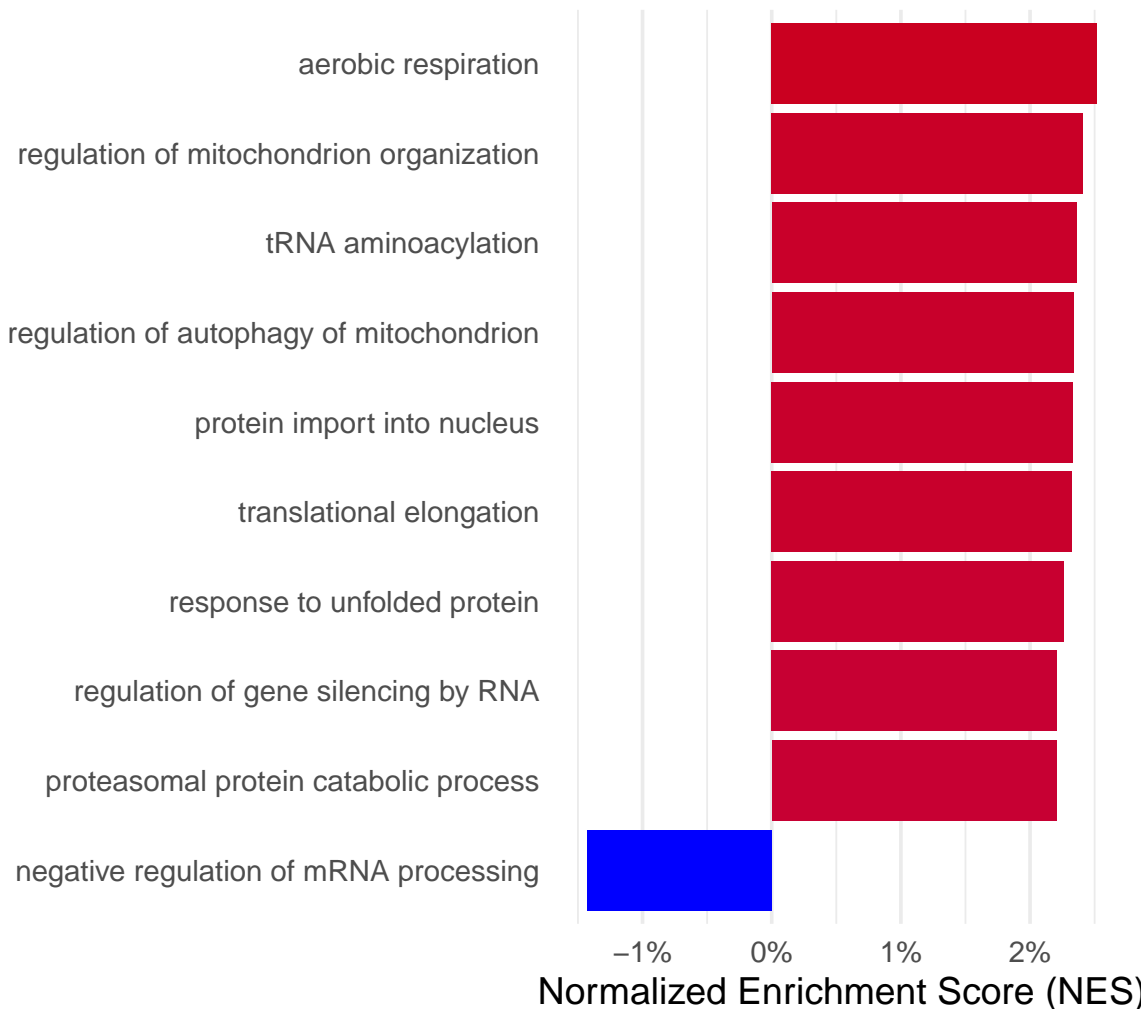

Supplement: Supplementary file 13 — Source Data [file 41467_2025_60542_MOESM13_ESM.zip › Source_data/Figure_3/E/E.pdf]

Cps1\_protein

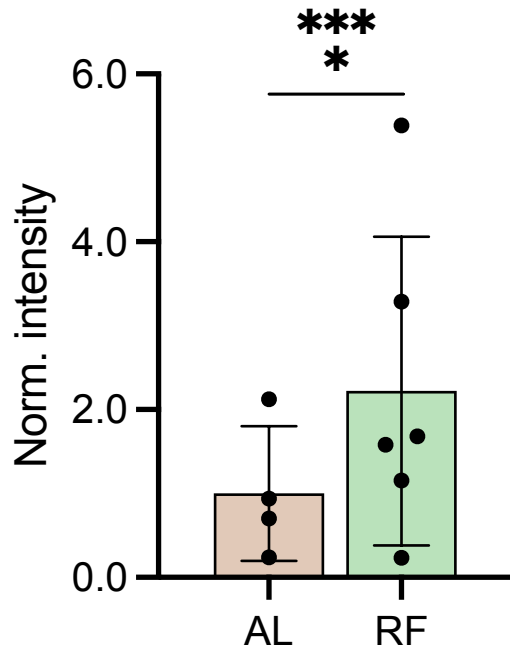

Arg1\_Protein

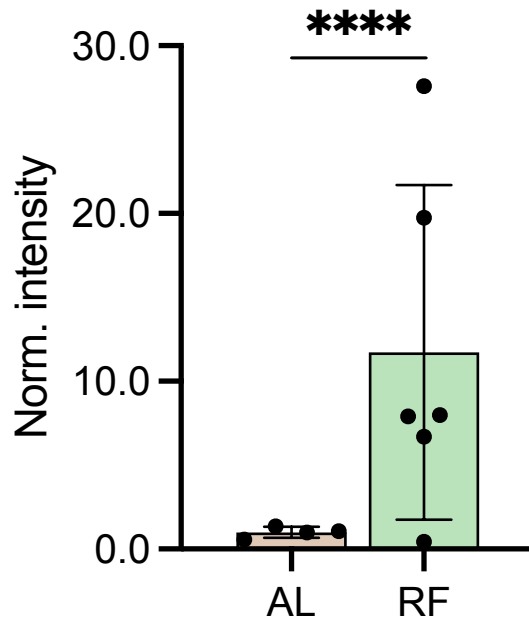

Supplement: Supplementary file 13 — Source Data [file 41467_2025_60542_MOESM13_ESM.zip › Source_data/Figure_S9/A/A.pdf]

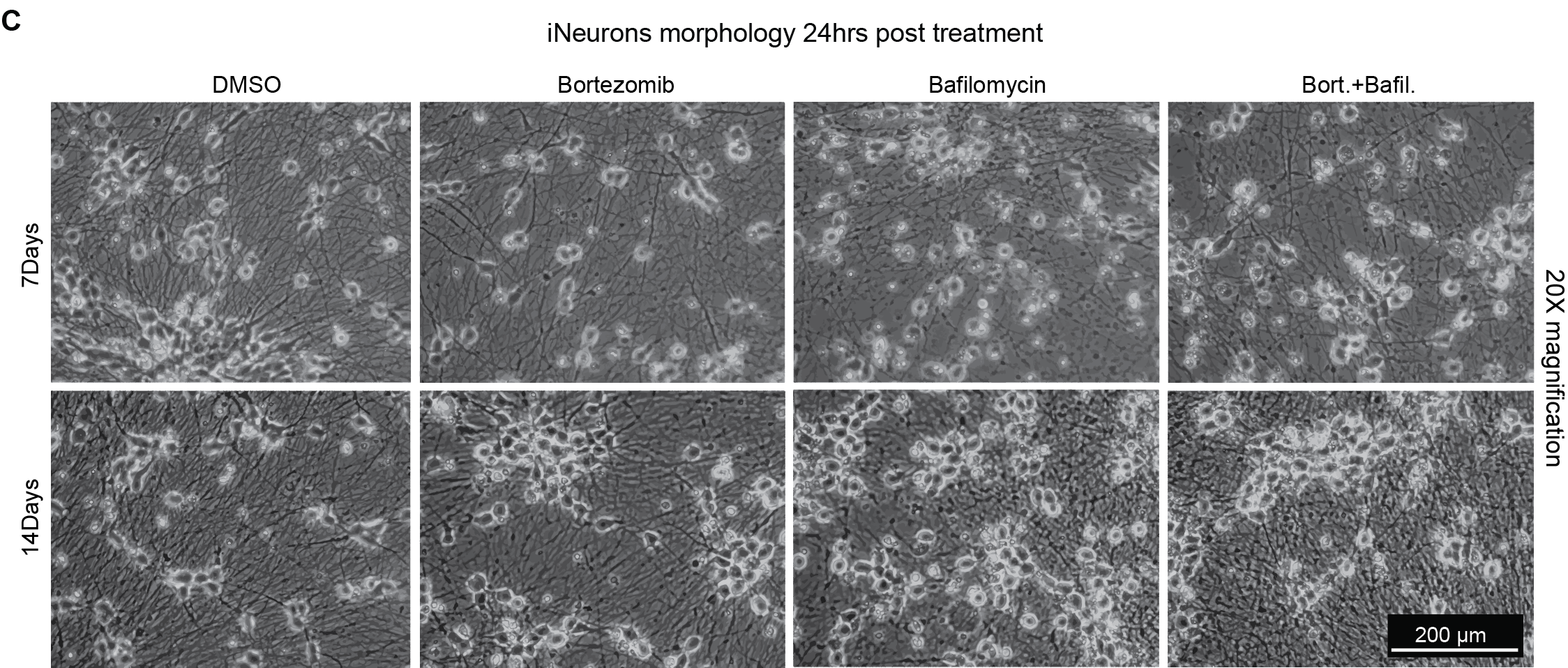

Supplement: Supplementary file 13 — Source Data [file 41467_2025_60542_MOESM13_ESM.zip › Source_data/Figure_S6/B/B.png]

14 Day

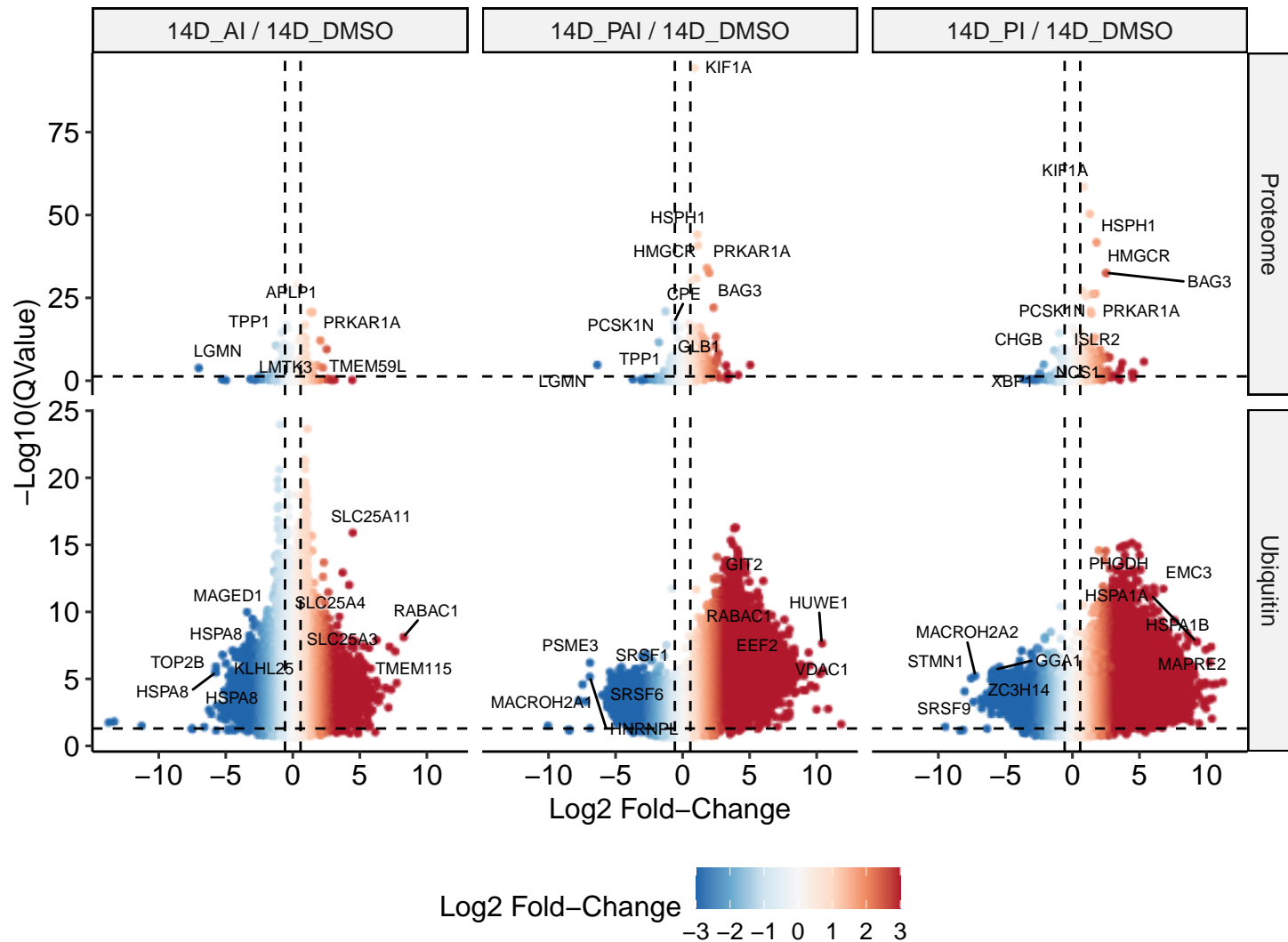

Supplement: Supplementary file 13 — Source Data [file 41467_2025_60542_MOESM13_ESM.zip › Source_data/Figure_S8/Left_panel/Left_panel.pdf]

# Individuals – PCA

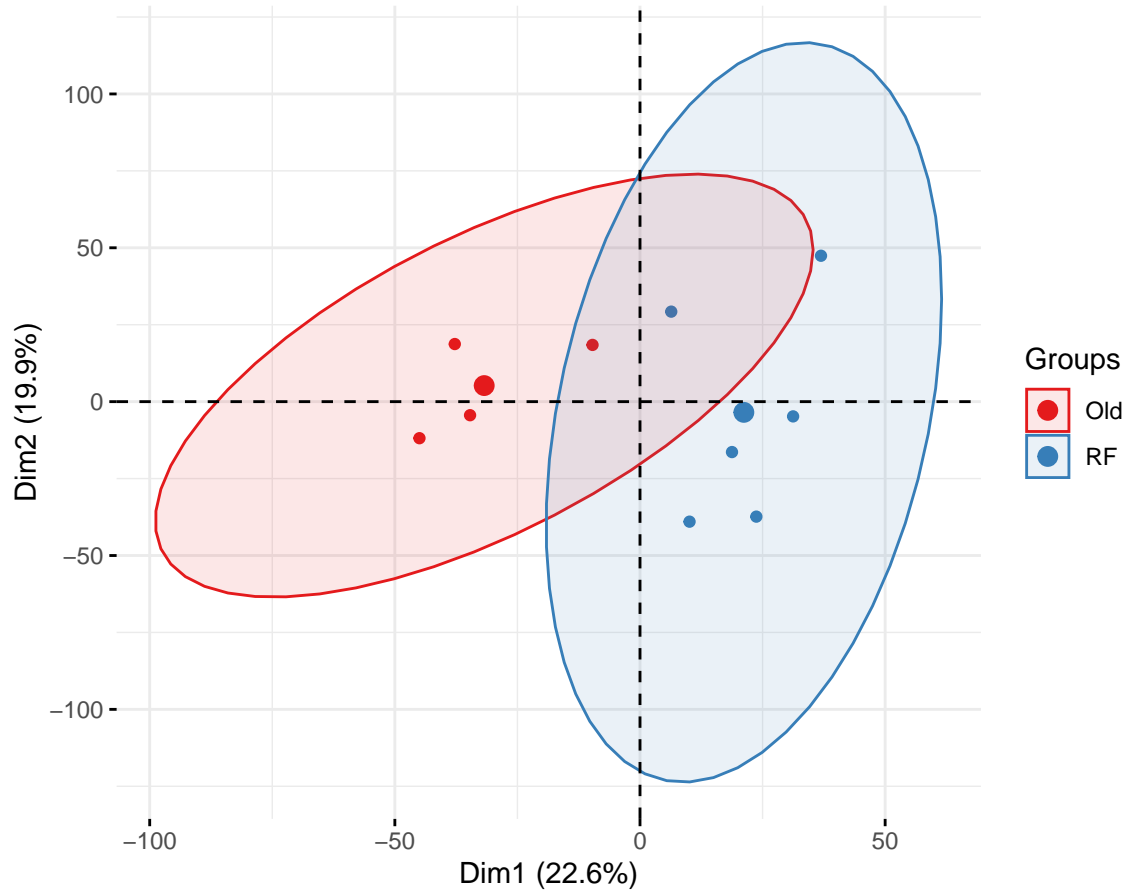

Supplement: Supplementary file 13 — Source Data [file 41467_2025_60542_MOESM13_ESM.zip › Source_data/Figure_5/B/B_right.pdf]

# Individuals – PCA

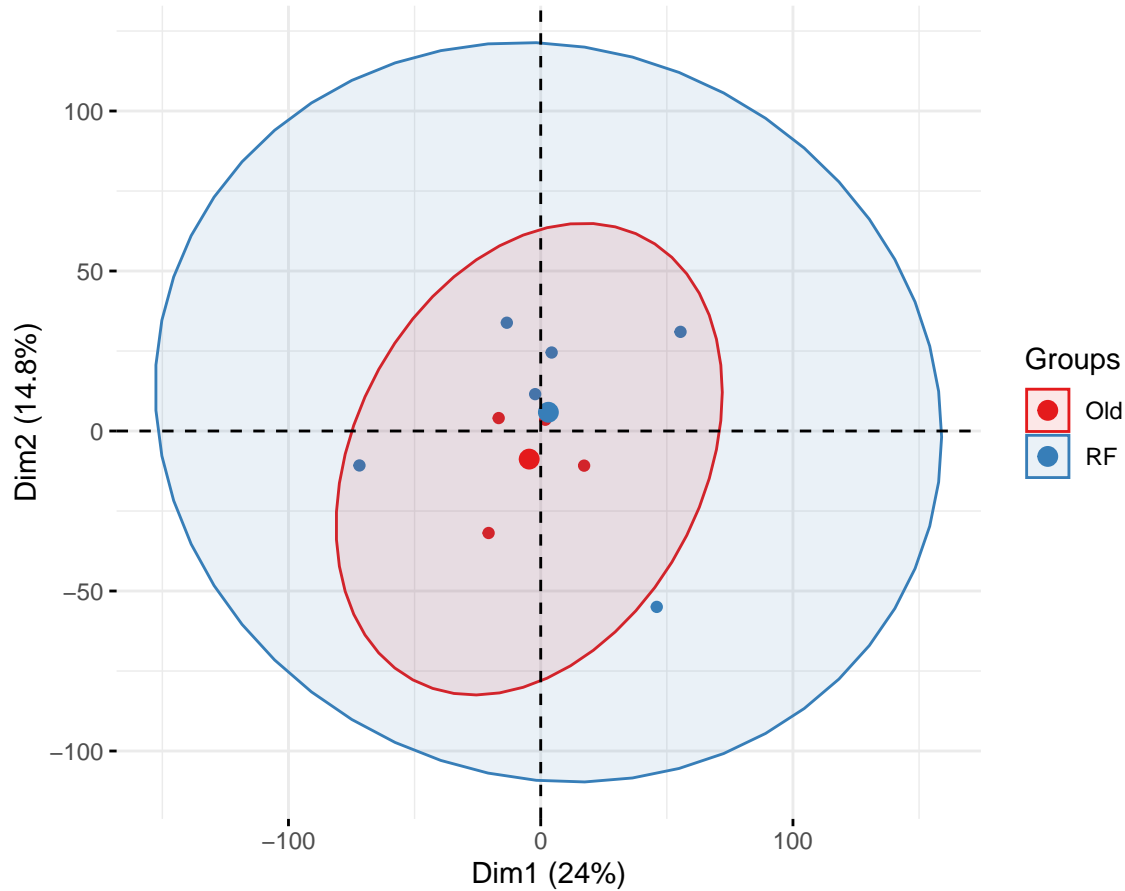

Supplement: Supplementary file 13 — Source Data [file 41467_2025_60542_MOESM13_ESM.zip › Source_data/Figure_5/B/B_left.pdf]

# Gene Ontology Enrichment – Biol

14DaysAI / 14DaysDMS

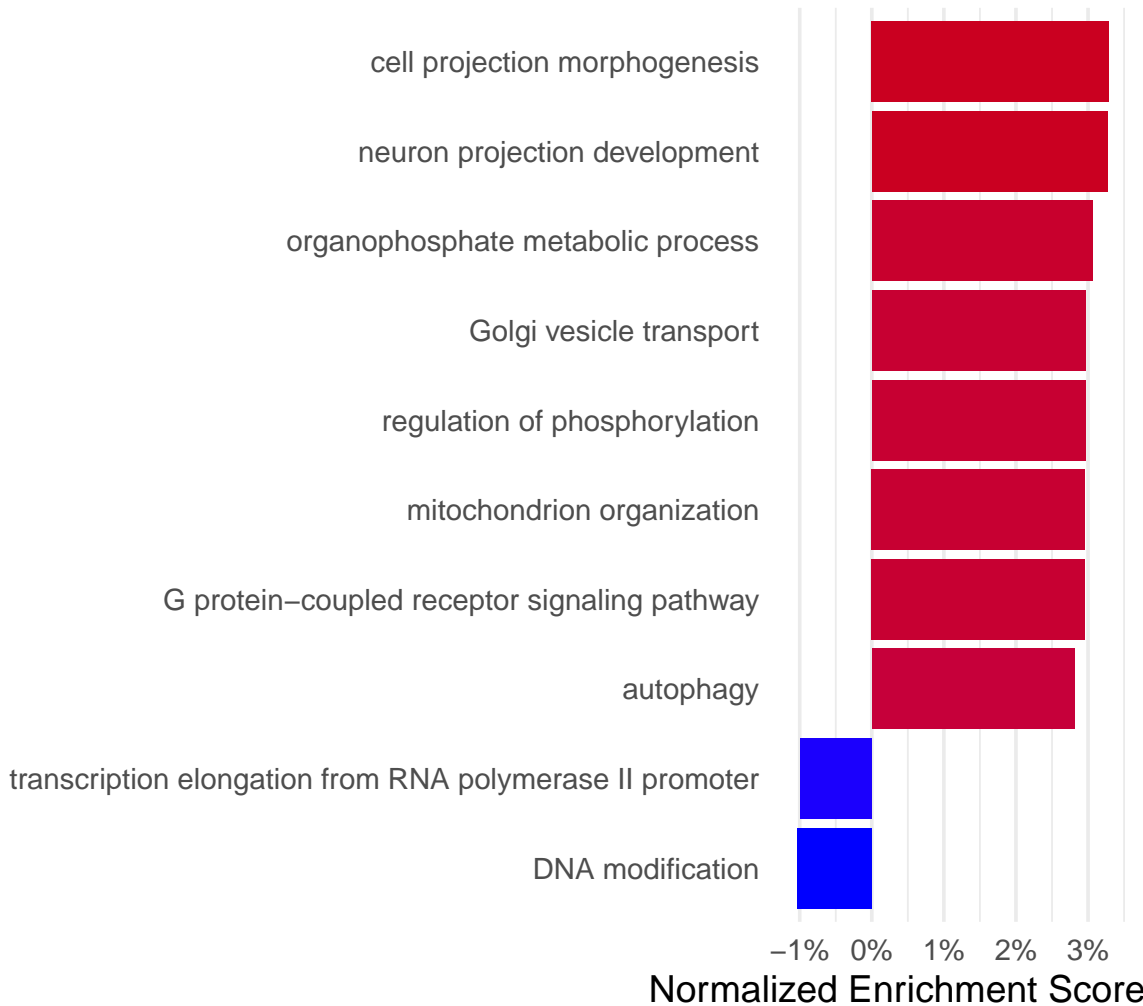

Supplement: Supplementary file 13 — Source Data [file 41467_2025_60542_MOESM13_ESM.zip › Source_data/Figure_3/F/F.pdf]

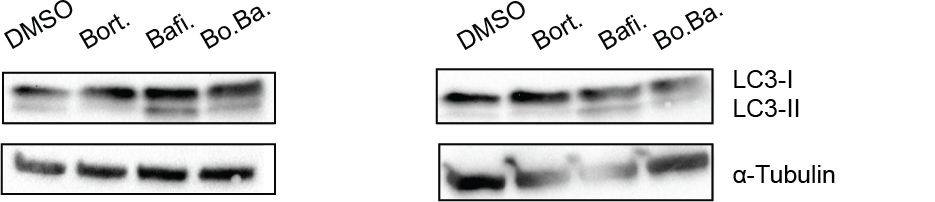

Supplement: Supplementary file 13 — Source Data [file 41467_2025_60542_MOESM13_ESM.zip › Source_data/Figure_S6/C_Bottom/C_bottom_panel.png]

quad    q1    q2    q3    q4

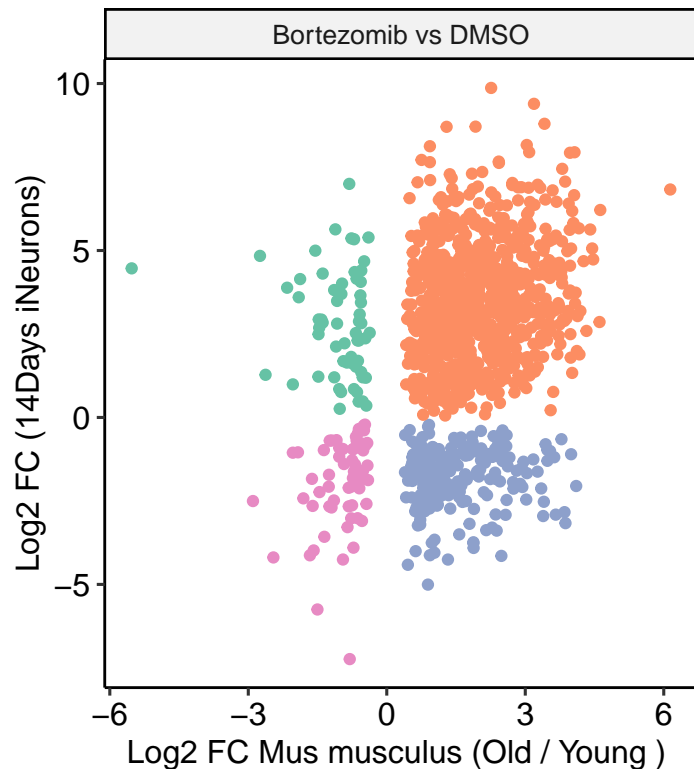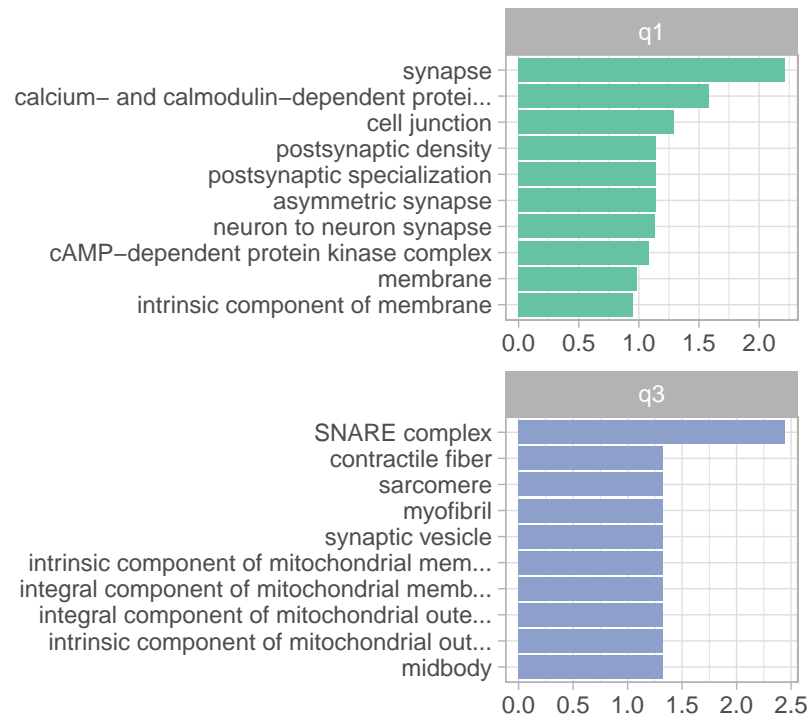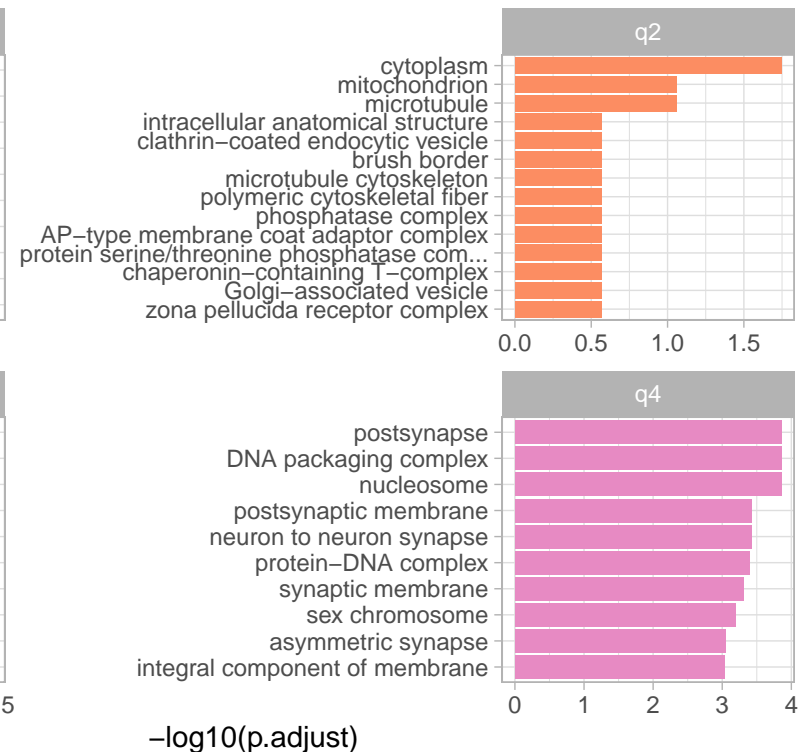

Supplement: Supplementary file 13 — Source Data [file 41467_2025_60542_MOESM13_ESM.zip › Source_data/Figure_S6/H/H.pdf]

# Individuals – PCA

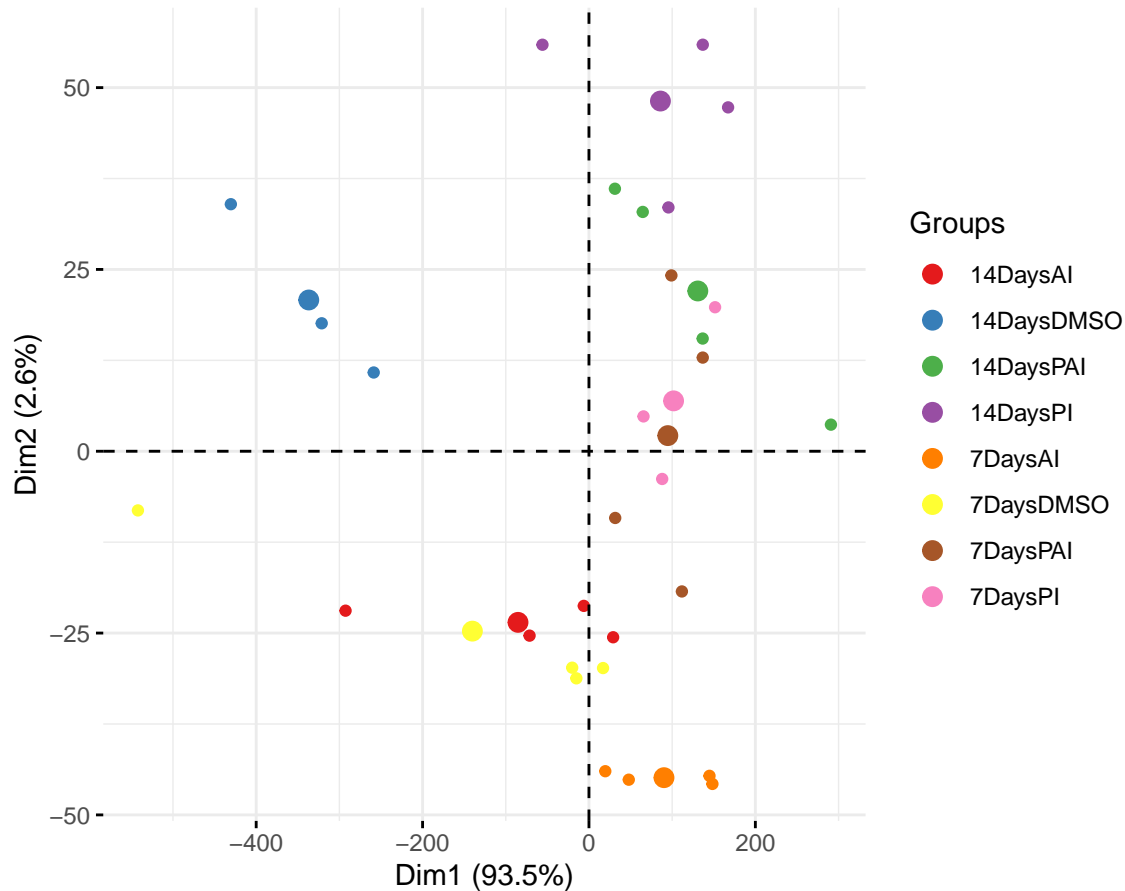

Supplement: Supplementary file 13 — Source Data [file 41467_2025_60542_MOESM13_ESM.zip › Source_data/Figure_3/C/C.pdf]

# Number of identified Ub sites

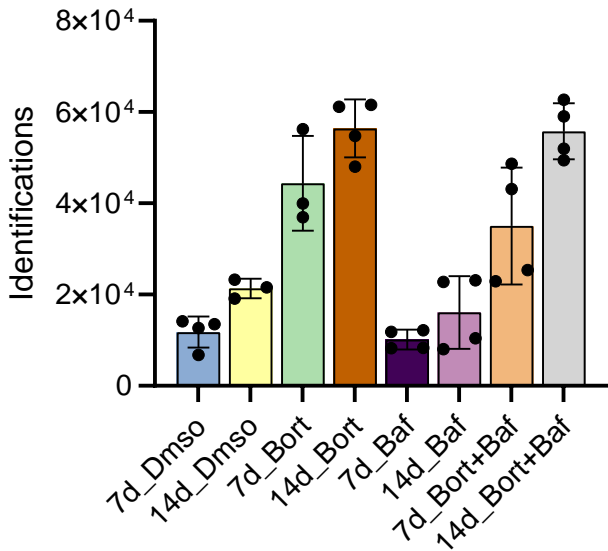

Supplement: Supplementary file 13 — Source Data [file 41467_2025_60542_MOESM13_ESM.zip › Source_data/Figure_3/D/D.pdf]

# % affected PTM sites during aging (OvsY)

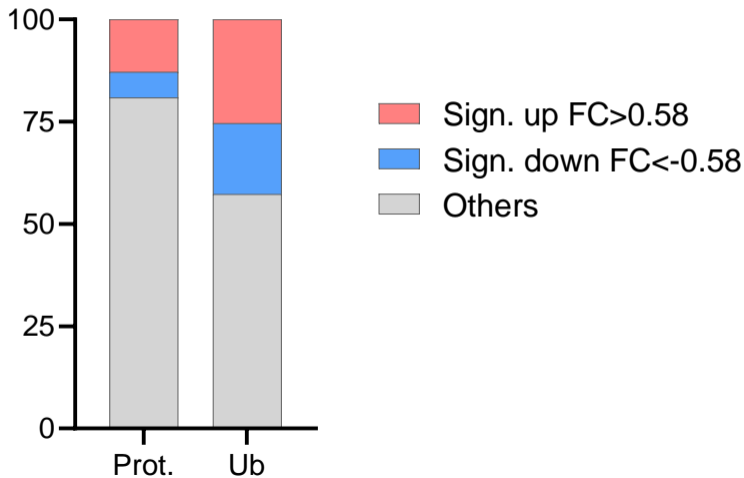

Supplement: Supplementary file 13 — Source Data [file 41467_2025_60542_MOESM13_ESM.zip › Source_data/Figure_S4/C/C.pdf]

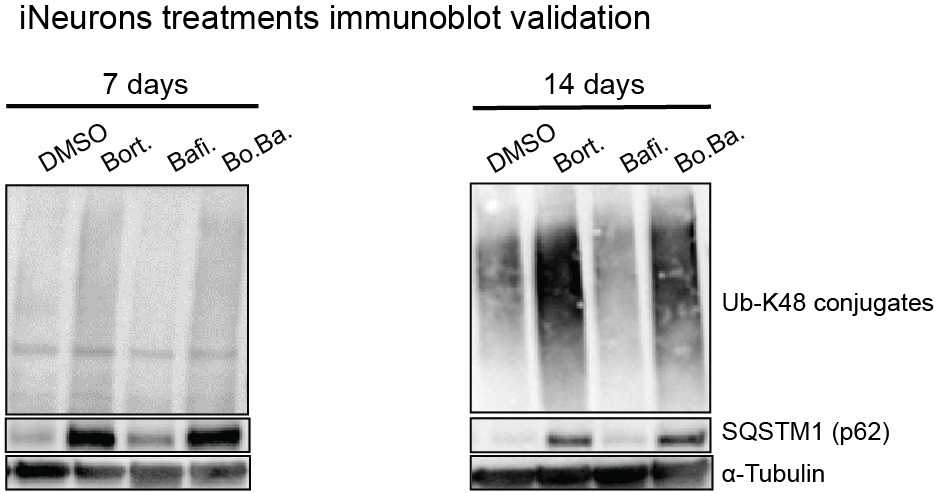

Supplement: Supplementary file 13 — Source Data [file 41467_2025_60542_MOESM13_ESM.zip › Source_data/Figure_S6/C_Top/C_top_panel.png]

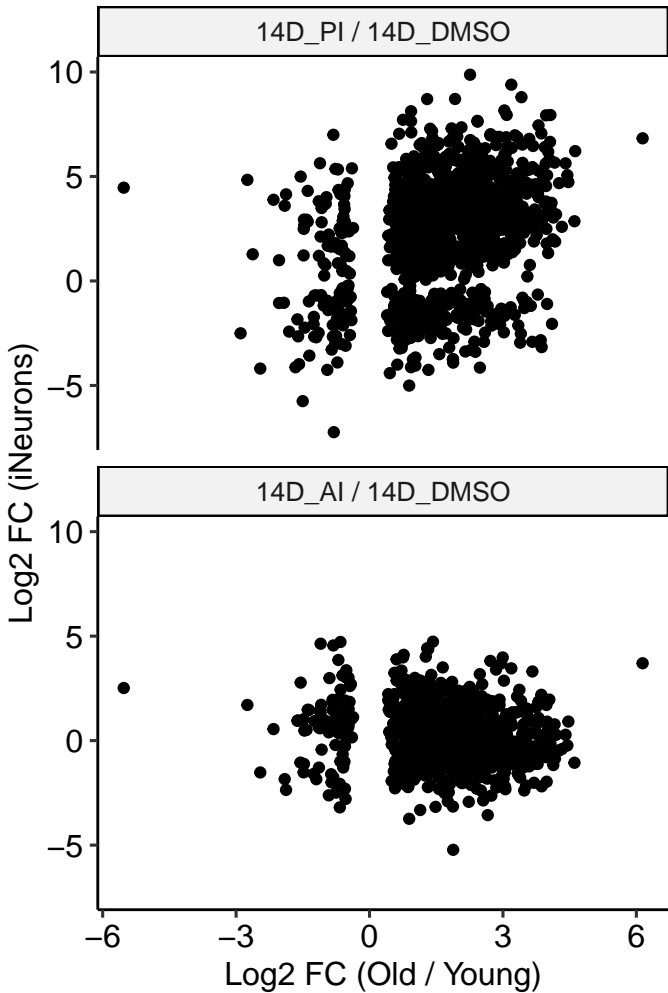

Supplement: Supplementary file 13 — Source Data [file 41467_2025_60542_MOESM13_ESM.zip › Source_data/Figure_3/H/H.pdf]

14 Day

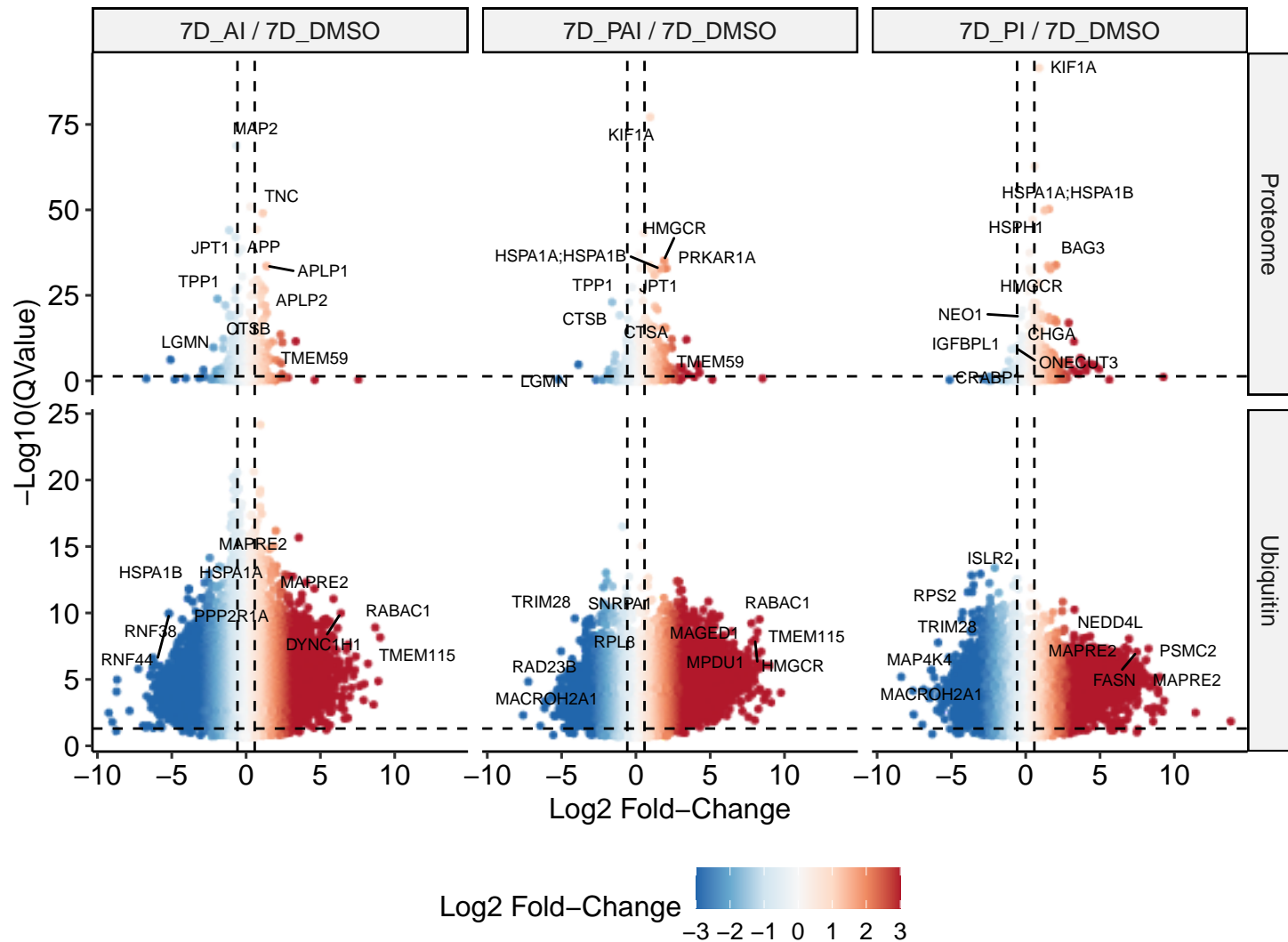

Supplement: Supplementary file 13 — Source Data [file 41467_2025_60542_MOESM13_ESM.zip › Source_data/Figure_S8/Right_panel/Right_panel.pdf]

# Gene Ontology Enrichment – Biologic

Proteome enrichment – OvsY – P.

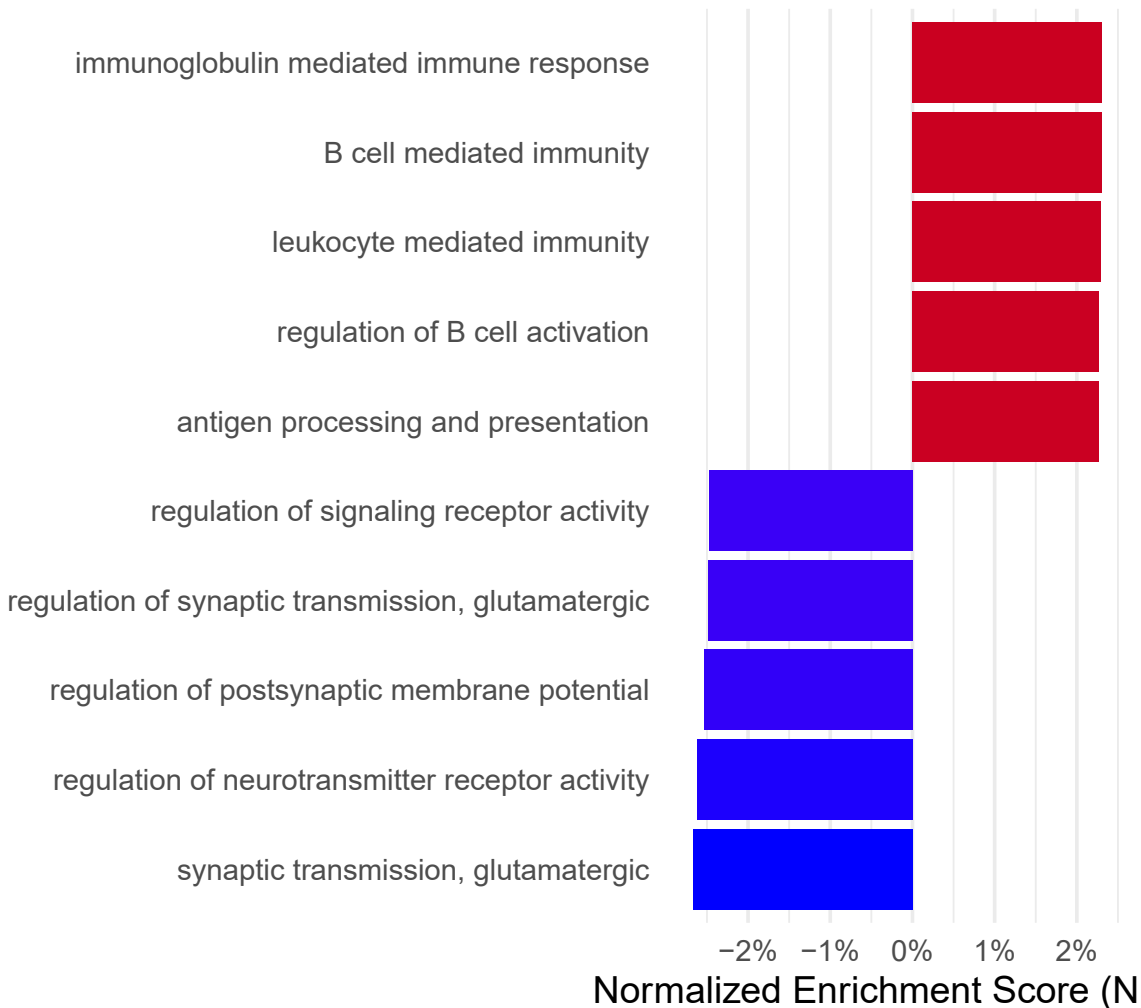

Supplement: Supplementary file 13 — Source Data [file 41467_2025_60542_MOESM13_ESM.zip › Source_data/Figure_S3/B/B.pdf]

# Number of identified proteins

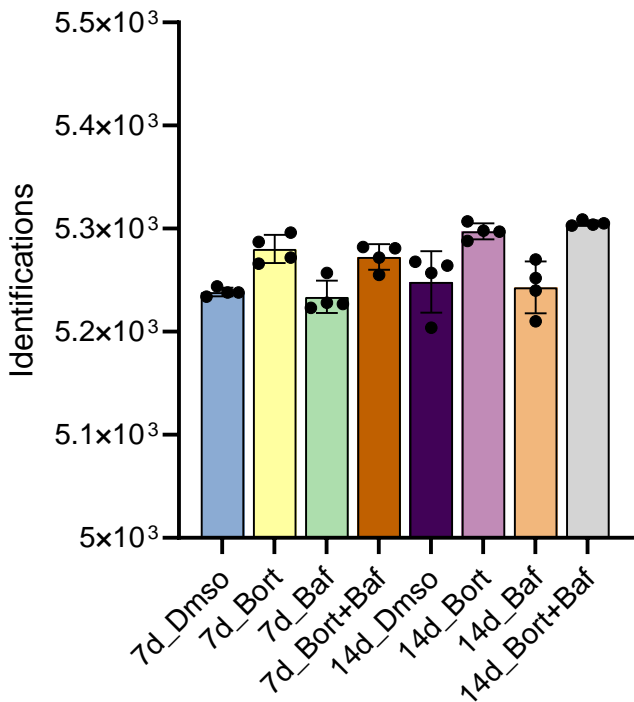

Supplement: Supplementary file 13 — Source Data [file 41467_2025_60542_MOESM13_ESM.zip › Source_data/Figure_S6/D/C.pdf]

SQSTM1\_K13\_Mouse (Ub)

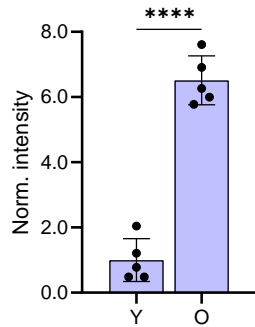

SQSTM1\_Protein\_Mouse

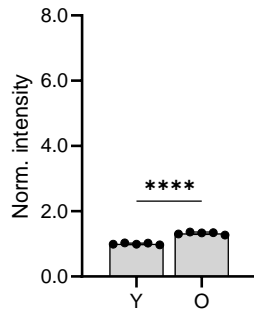

SQSTM1\_K13\_Human (Ub)

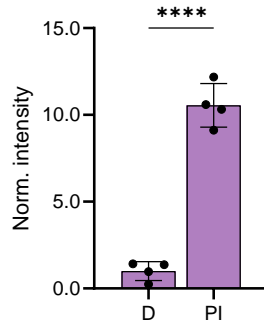

SQSTM1 Protein\_Human

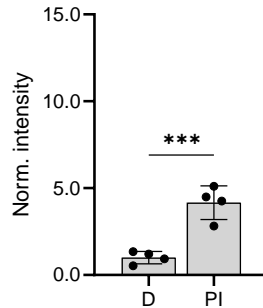

Supplement: Supplementary file 13 — Source Data [file 41467_2025_60542_MOESM13_ESM.zip › Source_data/Figure_S6/E/D.pdf]

# Gene Ontology Enrichment – Biological

Ph enrichment – OvsY – P.adj.  $\leq 0.0$

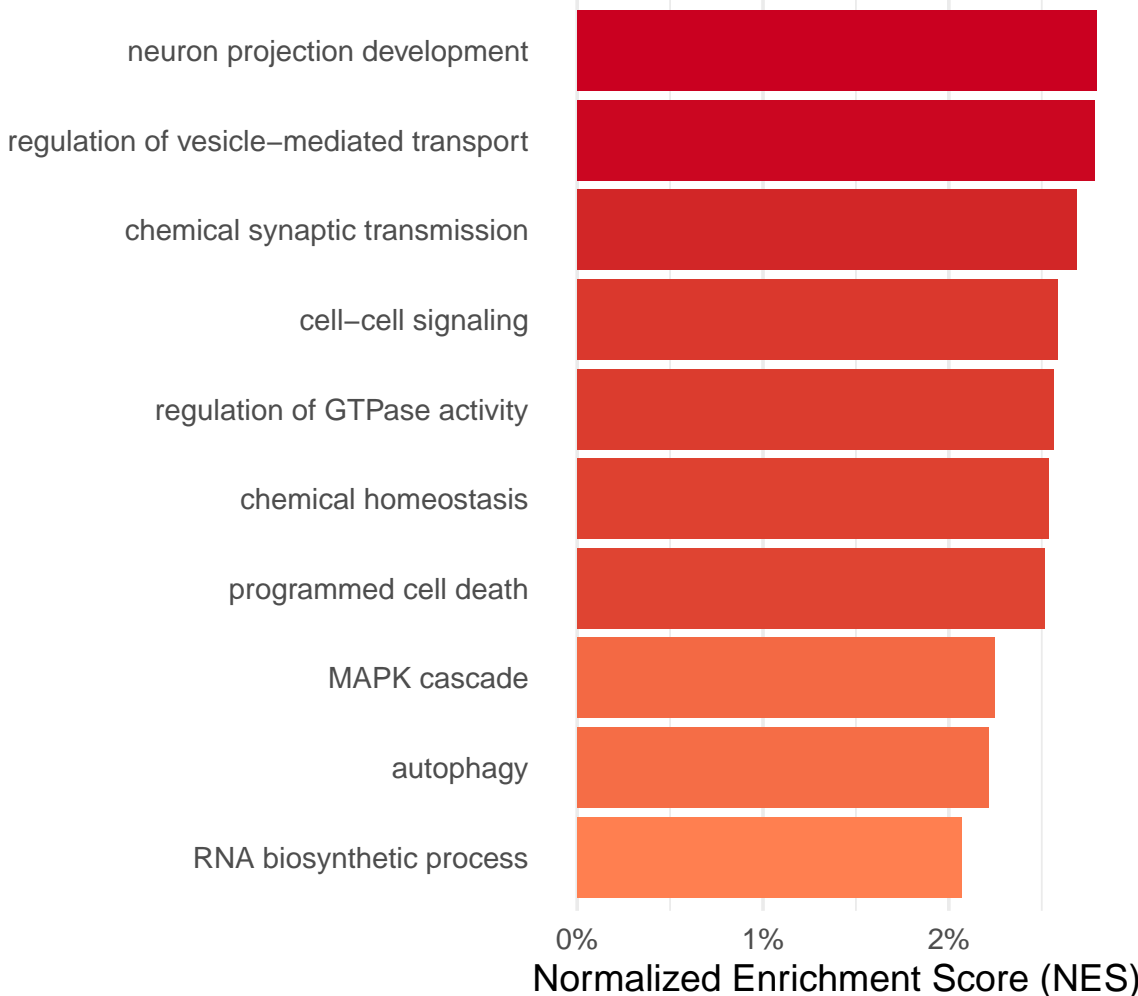

Supplement: Supplementary file 13 — Source Data [file 41467_2025_60542_MOESM13_ESM.zip › Source_data/Figure_S1/B/B.pdf]

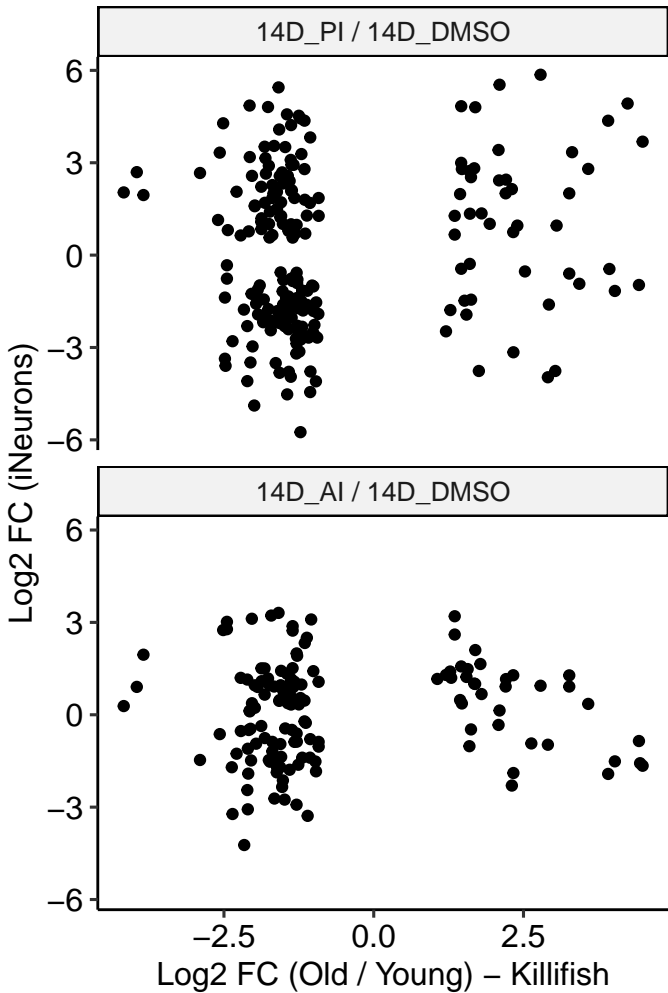

Supplement: Supplementary file 13 — Source Data [file 41467_2025_60542_MOESM13_ESM.zip › Source_data/Figure_S6/G/G.pdf]

# Gene Ontology Enrichment – Bi

Transcriptome enrichment –

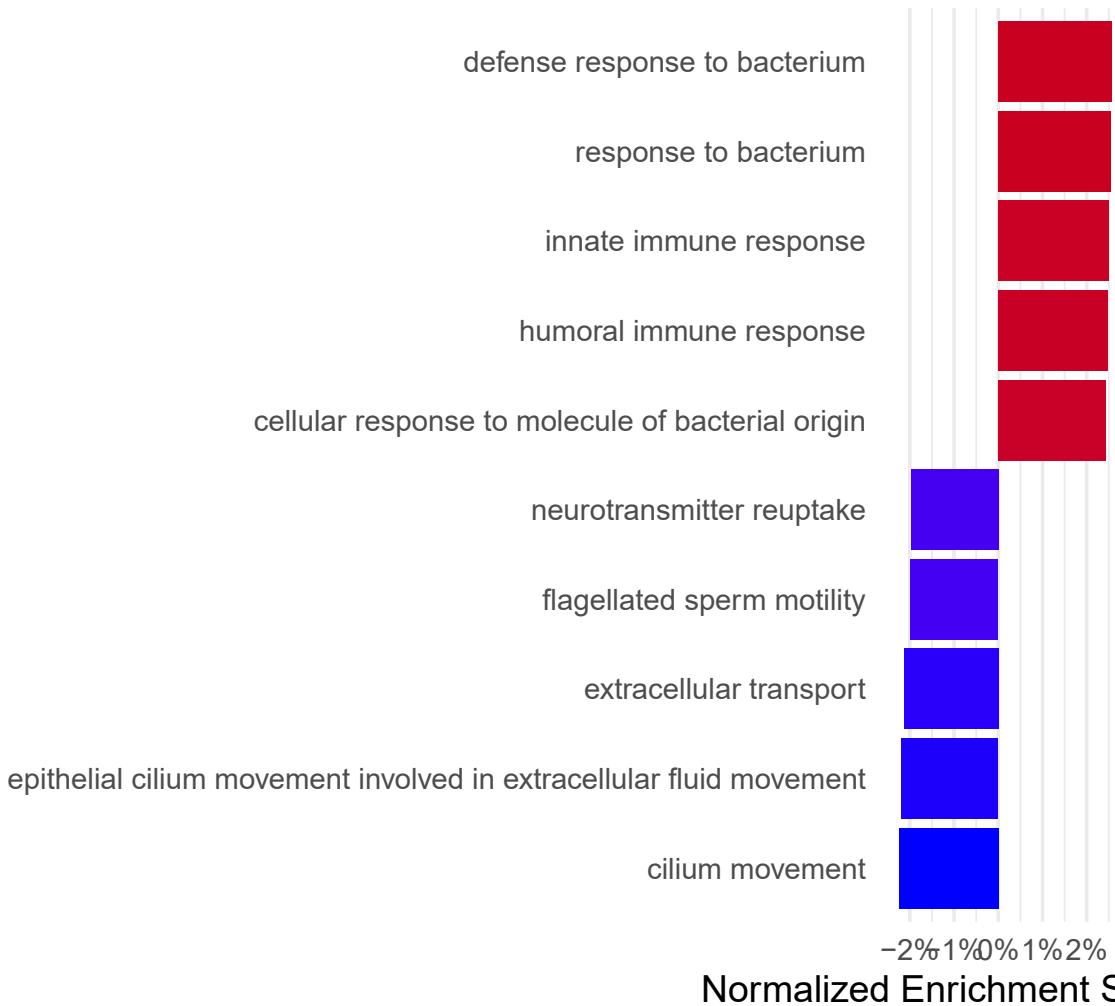

Supplement: Supplementary file 13 — Source Data [file 41467_2025_60542_MOESM13_ESM.zip › Source_data/Figure_S3/E/E.pdf]

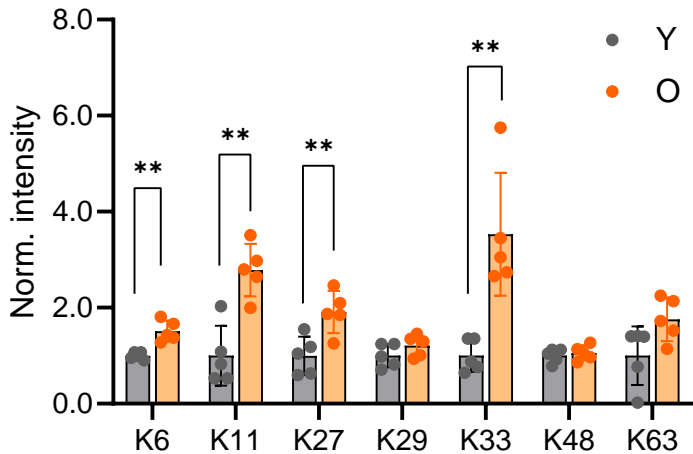

Supplement: Supplementary file 13 — Source Data [file 41467_2025_60542_MOESM13_ESM.zip › Source_data/Figure_S2/H/H.pdf]

common.cond.y

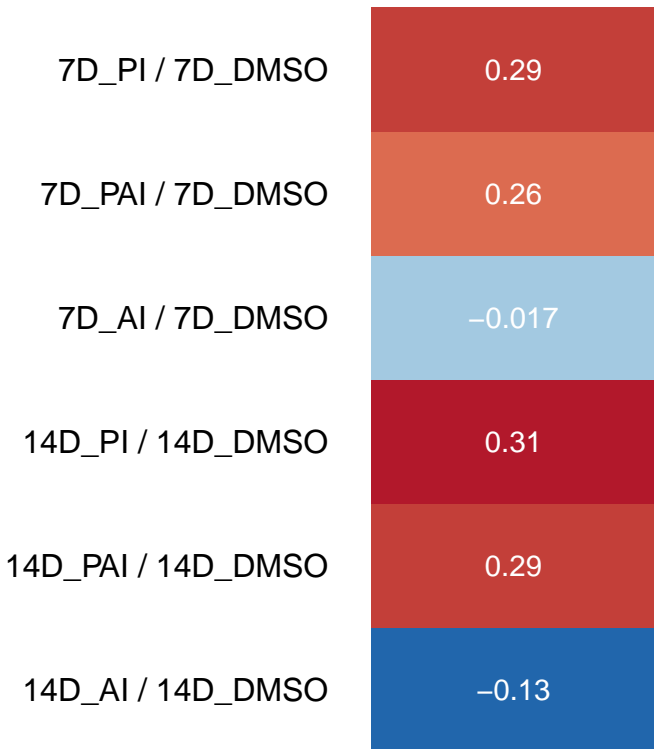

Pearson's R: 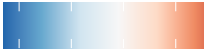  
-0.1 0.0 0.1 0.2

Supplement: Supplementary file 13 — Source Data [file 41467_2025_60542_MOESM13_ESM.zip › Source_data/Figure_3/G/G.pdf]

Rap1a\_K104 (Ub)

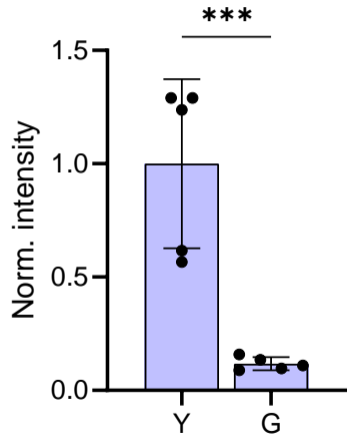

Rap1a\_K151 (Ub)

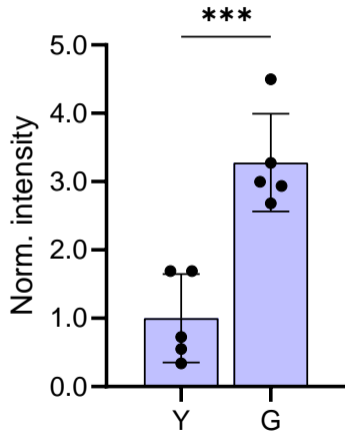

Rap1a\_Protein

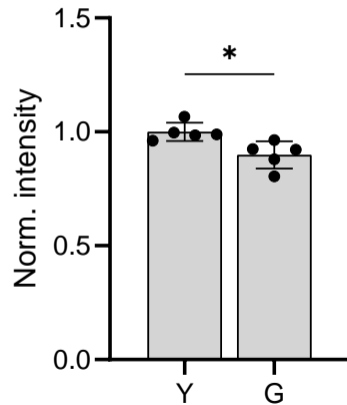

Supplement: Supplementary file 13 — Source Data [file 41467_2025_60542_MOESM13_ESM.zip › Source_data/Figure_S2/E/E.pdf]

# Correlation PTM vs Whole Proteome Fold Changes RF/AL

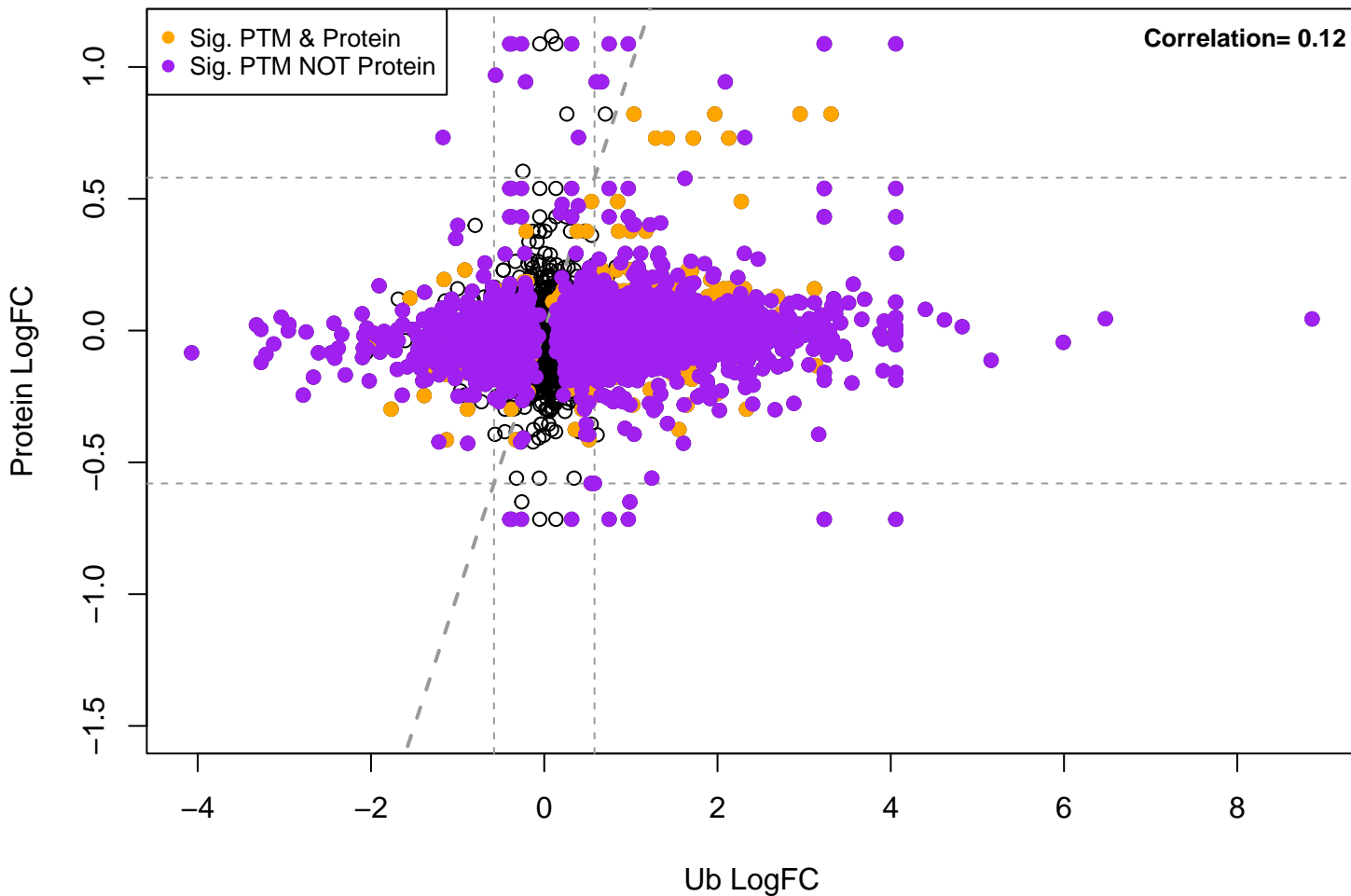

Supplement: Supplementary file 13 — Source Data [file 41467_2025_60542_MOESM13_ESM.zip › Source_data/Figure_S9/C/F.pdf]

Individuals – PCA

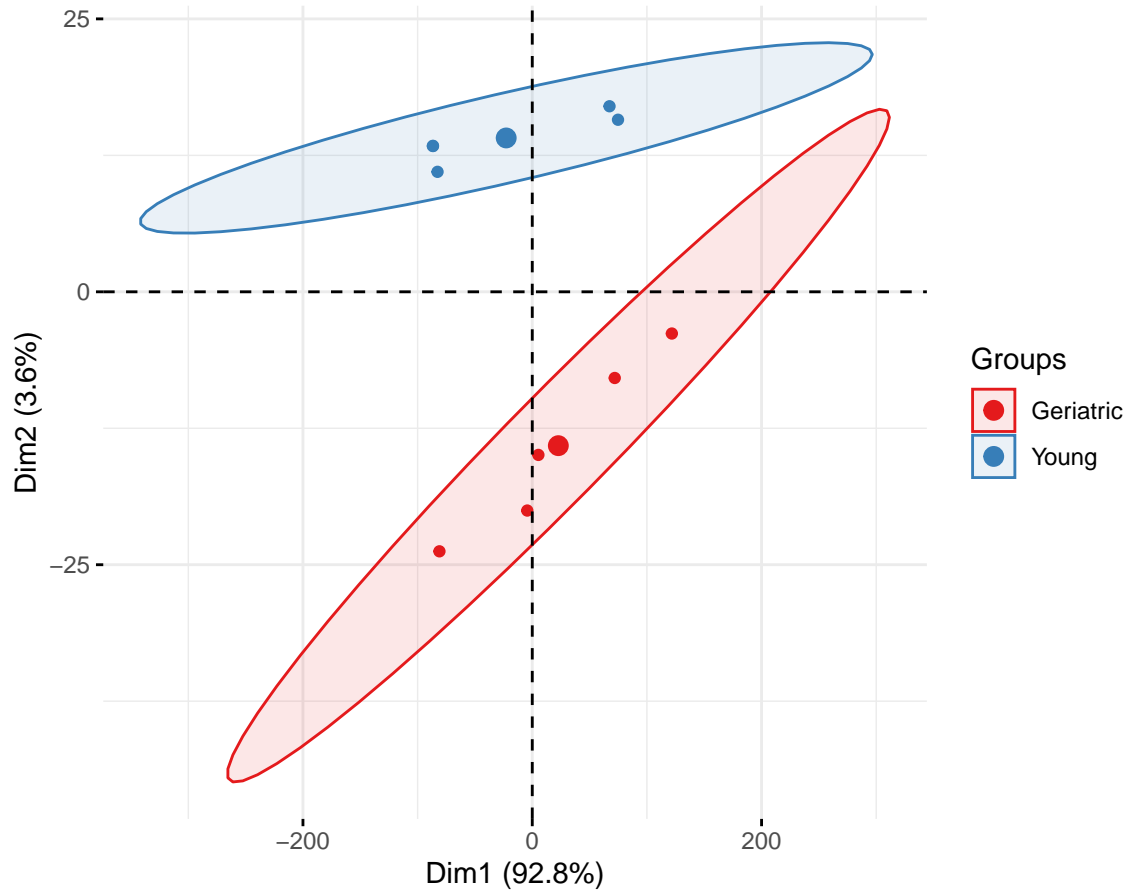

Supplement: Supplementary file 13 — Source Data [file 41467_2025_60542_MOESM13_ESM.zip › Source_data/Figure_1/C/C.pdf]

# Individuals – PCA

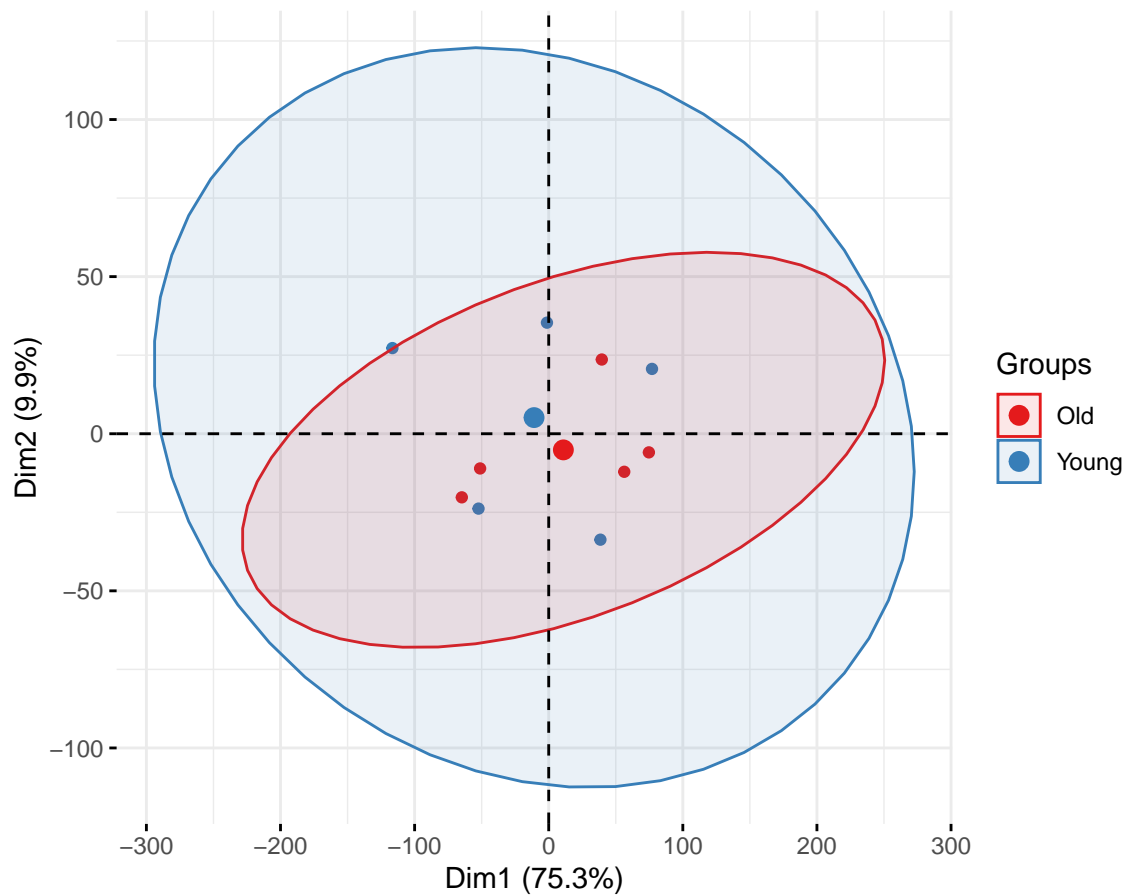

Supplement: Supplementary file 13 — Source Data [file 41467_2025_60542_MOESM13_ESM.zip › Source_data/Figure_S1/D/D.pdf]

# Proteome - Mouse brain aging OvsY

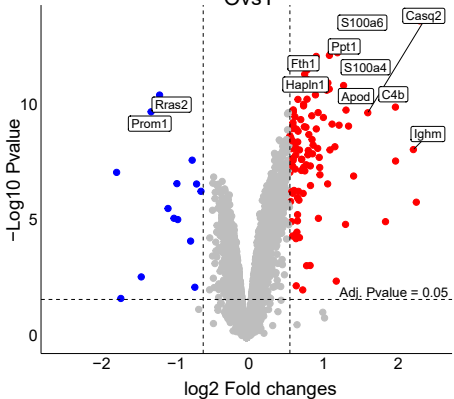

Supplement: Supplementary file 13 — Source Data [file 41467_2025_60542_MOESM13_ESM.zip › Source_data/Figure_S3/C/C.pdf]

# Individuals – PCA

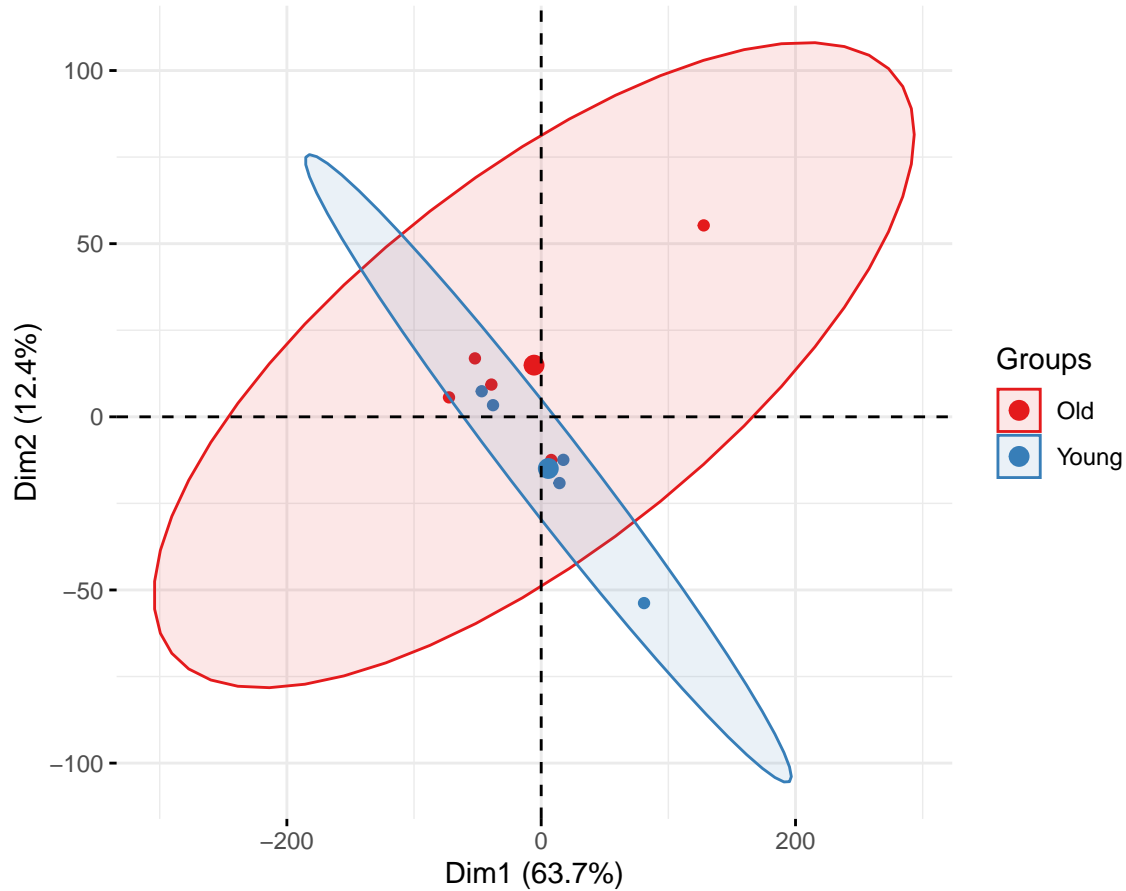

Supplement: Supplementary file 13 — Source Data [file 41467_2025_60542_MOESM13_ESM.zip › Source_data/Figure_S1/A/A.pdf]

**Proteasomal activity: Chymotrypsin-like**  
**Mouse Brains: Normalized against MG132 samples**

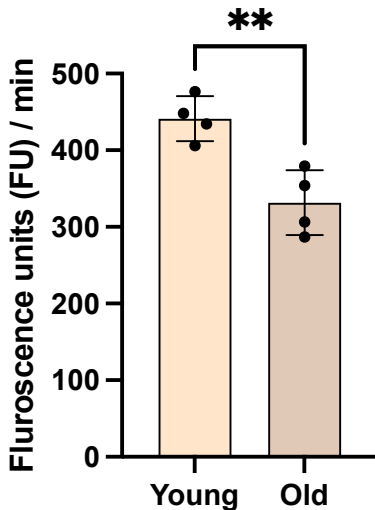

Supplement: Supplementary file 13 — Source Data [file 41467_2025_60542_MOESM13_ESM.zip › Source_data/Figure_S6/A/A.pdf]

Elfn2\_K644 (Ub)

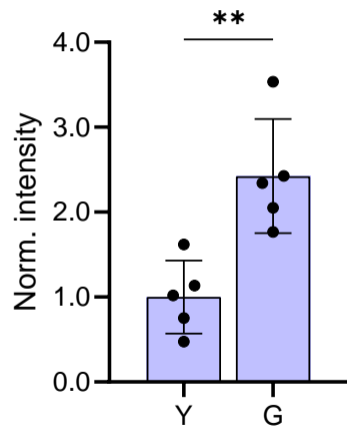

Elfn2\_K485 (Ub)

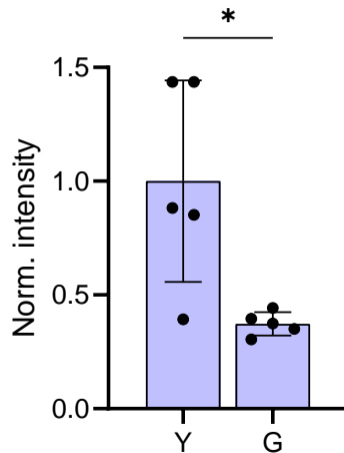

Elfn2\_Protein

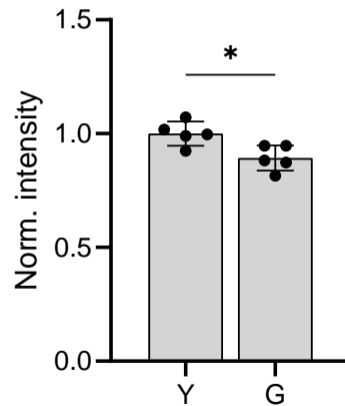

Supplement: Supplementary file 13 — Source Data [file 41467_2025_60542_MOESM13_ESM.zip › Source_data/Figure_S2/G/G.pdf]

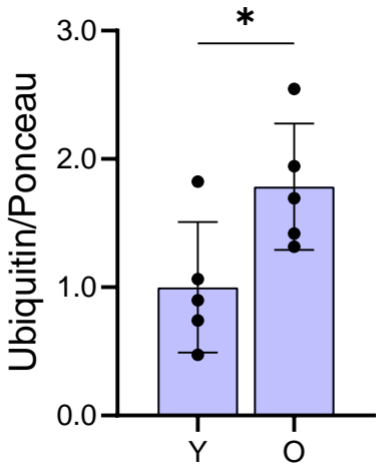

Supplement: Supplementary file 13 — Source Data [file 41467_2025_60542_MOESM13_ESM.zip › Source_data/Figure_S2/C/C.pdf]

Gsn\_K358 (Ub)

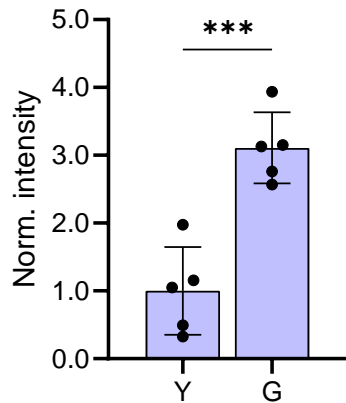

Gsn\_K366 (Ub)

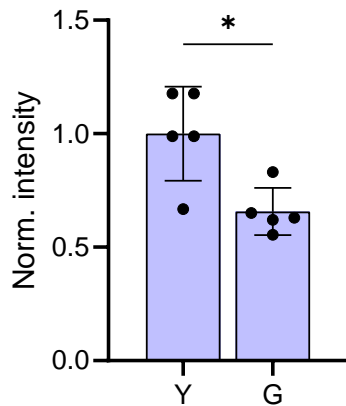

Gsn\_Protein

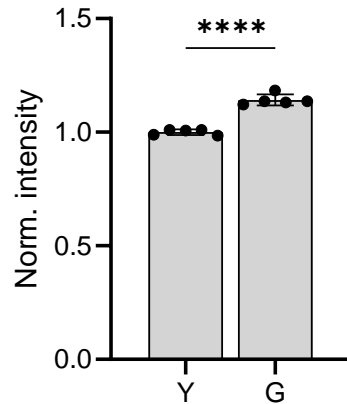

Supplement: Supplementary file 13 — Source Data [file 41467_2025_60542_MOESM13_ESM.zip › Source_data/Figure_S2/D/D.pdf]

# Number of identified Ub sites

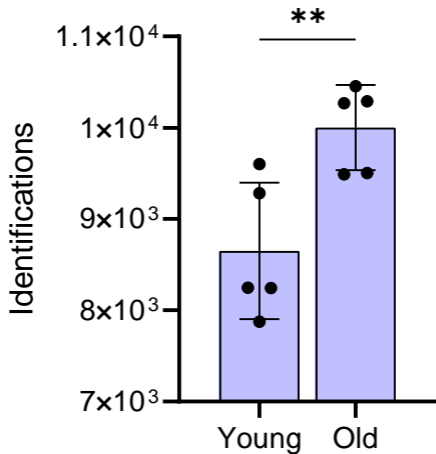

Supplement: Supplementary file 13 — Source Data [file 41467_2025_60542_MOESM13_ESM.zip › Source_data/Figure_S2/B/B.pdf]

# Transcriptome - Mouse brain aging

OvsY

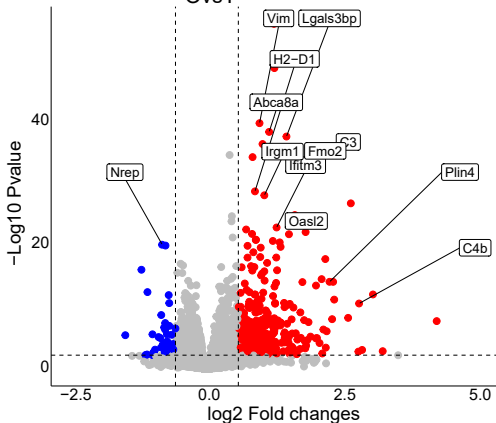

Supplement: Supplementary file 13 — Source Data [file 41467_2025_60542_MOESM13_ESM.zip › Source_data/Figure_S3/F/F.pdf]

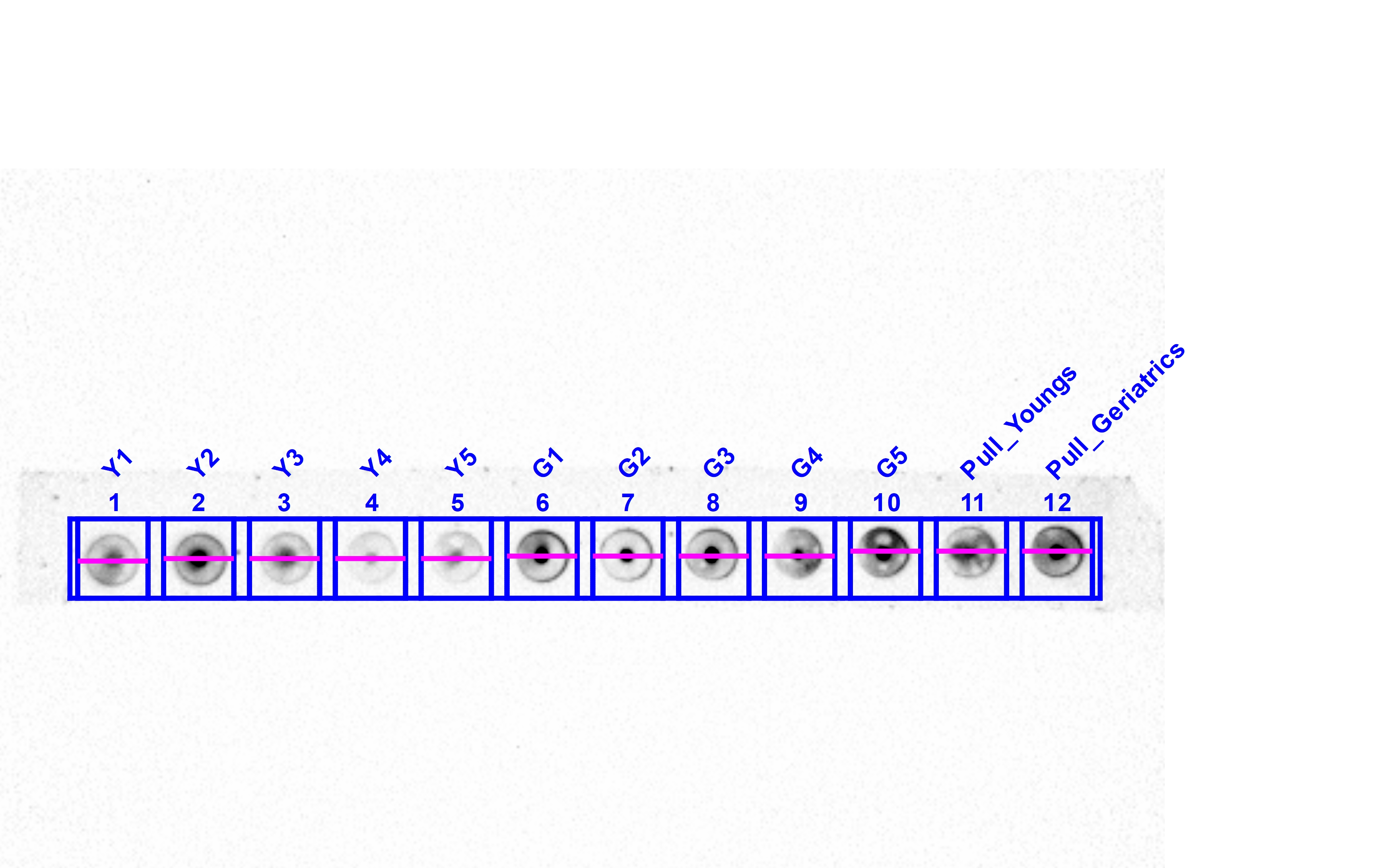

Supplement: Supplementary file 13 — Source Data [file 41467_2025_60542_MOESM13_ESM.zip › Source_data/Figure_S2/C/Ubiquitin_DotImage.jpg]

# Gene Ontology Enrichment – Biological Process

Ac enrichment – OvsY – P.adj.  $\leq 0.05$

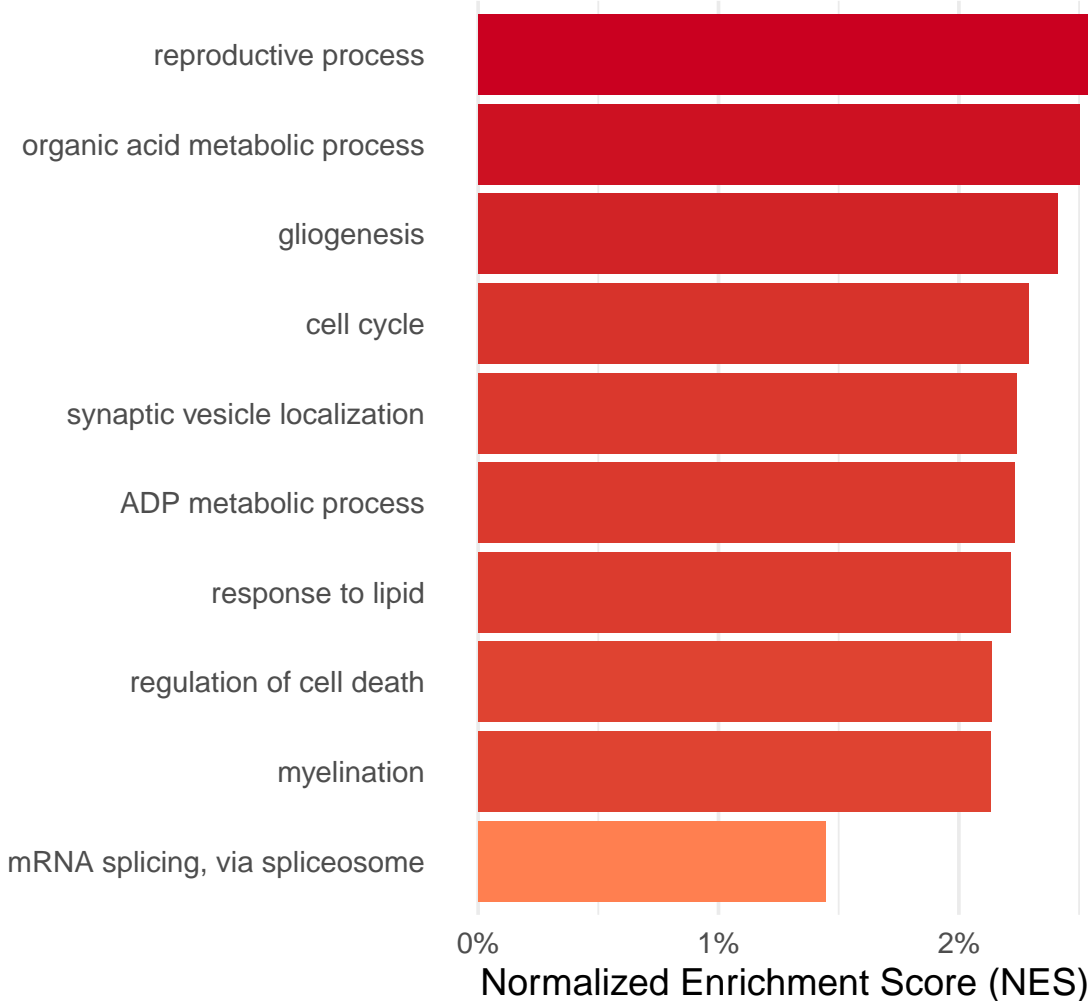

Supplement: Supplementary file 13 — Source Data [file 41467_2025_60542_MOESM13_ESM.zip › Source_data/Figure_S1/E/E.pdf]

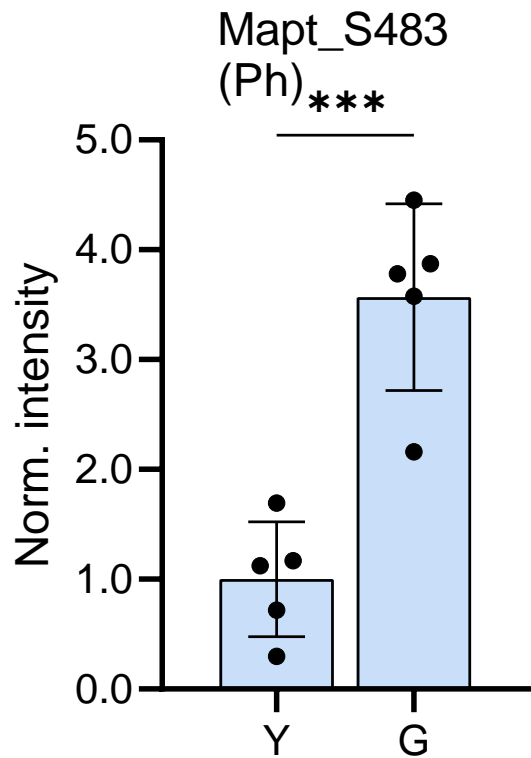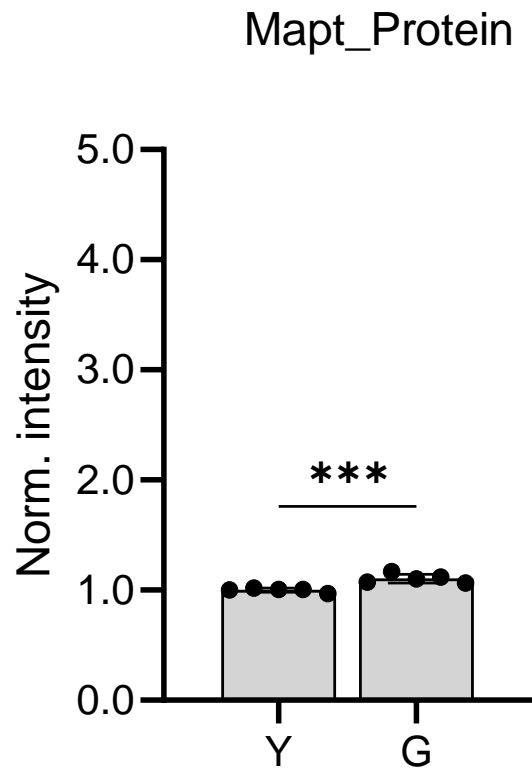

Supplement: Supplementary file 13 — Source Data [file 41467_2025_60542_MOESM13_ESM.zip › Source_data/Figure_S1/G/G.pdf]

Usp9x\_K218 (Ub)

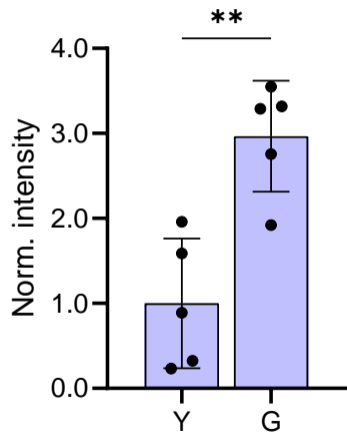

Usp9x\_K1899 (Ub)

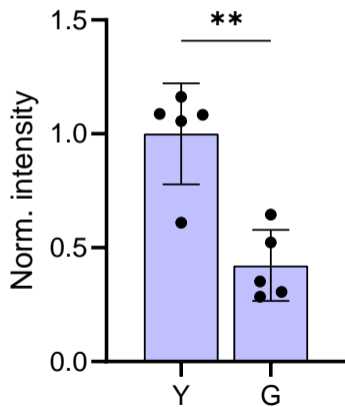

Usp9x\_Protein

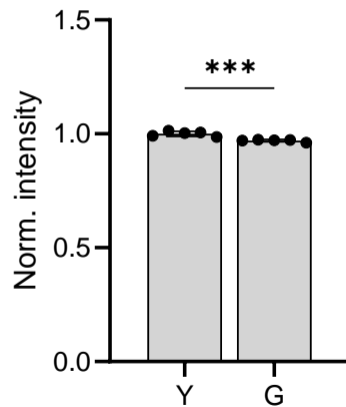

Supplement: Supplementary file 13 — Source Data [file 41467_2025_60542_MOESM13_ESM.zip › Source_data/Figure_S2/F/F.pdf]

# Number of Protein Groups or PTMs sites

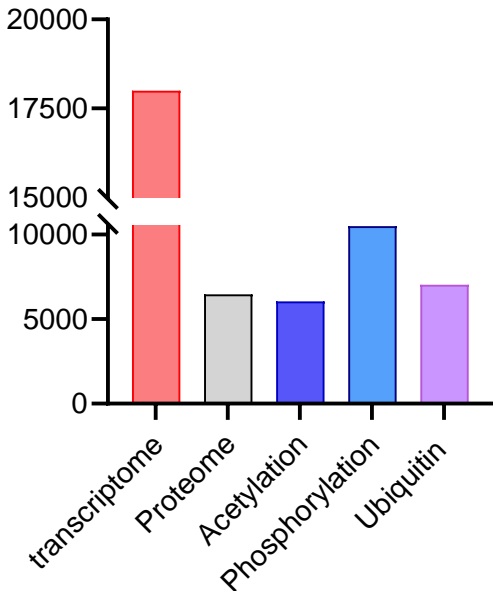

Supplement: Supplementary file 13 — Source Data [file 41467_2025_60542_MOESM13_ESM.zip › Source_data/Figure_S2/A/A.pdf]

# % affected PTM sites during aging (OvsY)

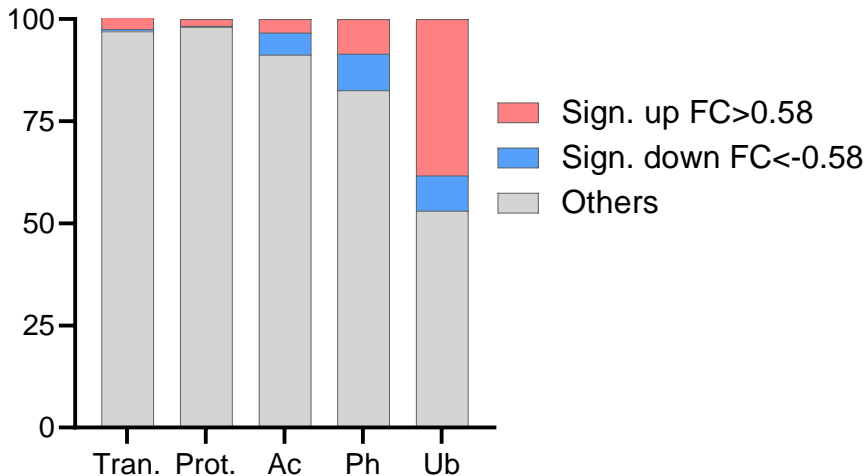

Supplement: Supplementary file 13 — Source Data [file 41467_2025_60542_MOESM13_ESM.zip › Source_data/Figure_1/B/B.pdf]

# Gene Ontology Enrichment – Cellul

Ub enrichment – OvsY – P.adj. <

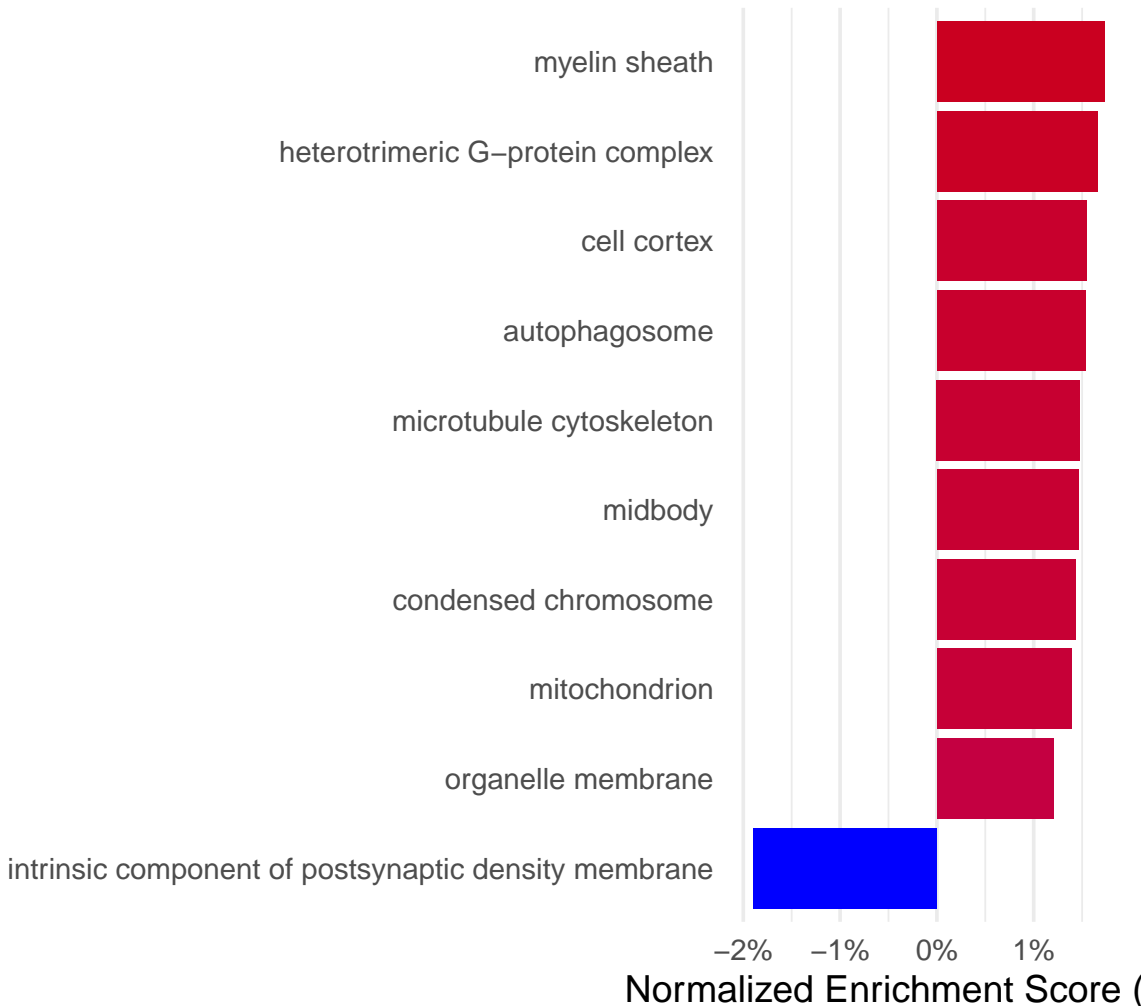

Supplement: Supplementary file 13 — Source Data [file 41467_2025_60542_MOESM13_ESM.zip › Source_data/Figure_1/E/E.pdf]

# Correlation PTM vs Whole Proteome Fold Changes

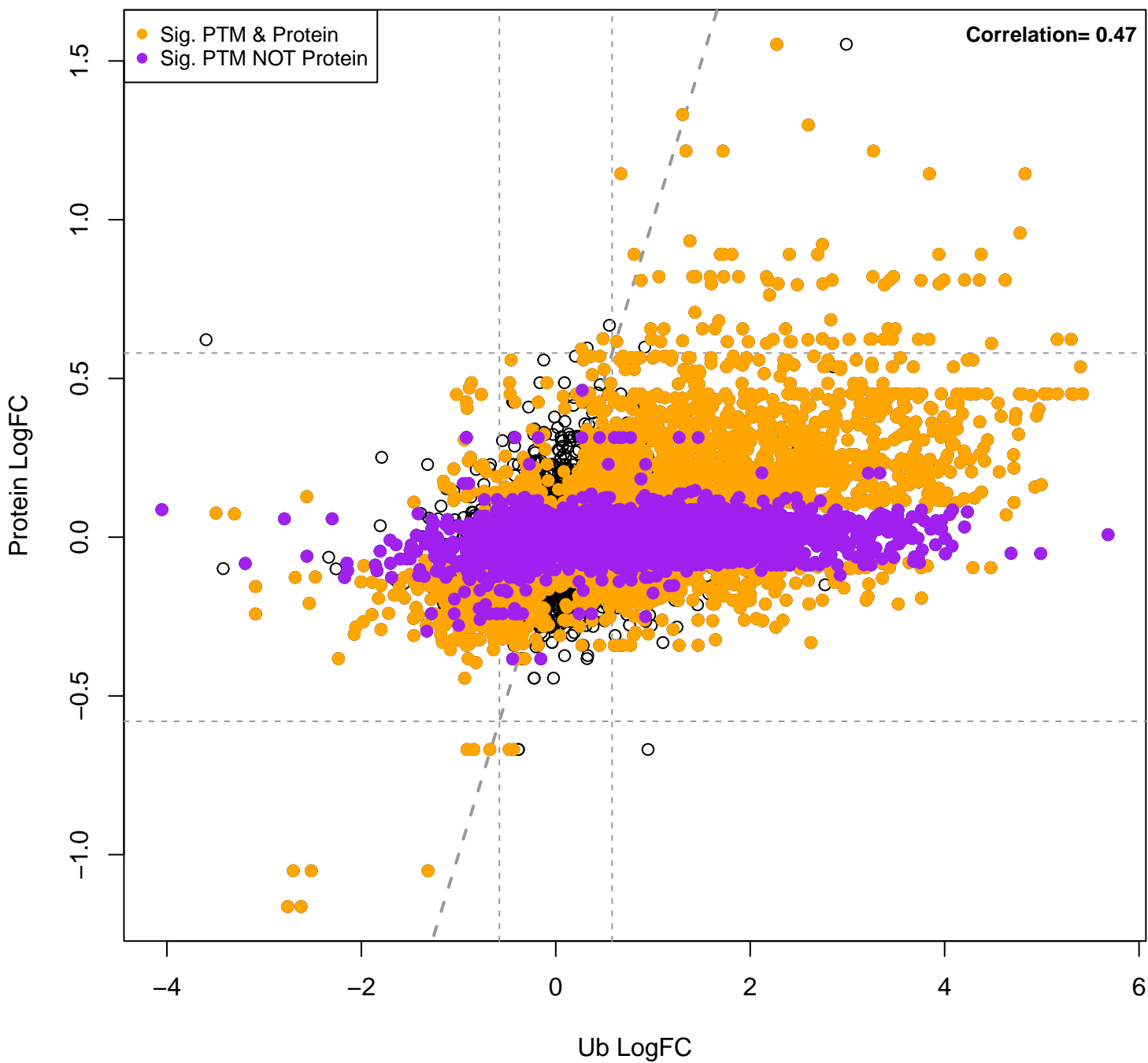

Supplement: Supplementary file 13 — Source Data [file 41467_2025_60542_MOESM13_ESM.zip › Source_data/Figure_1/F/F.pdf]

## Ub changes and Half-life

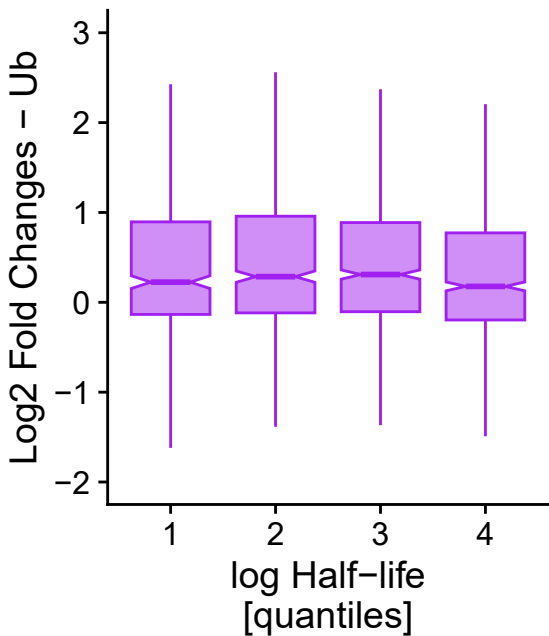

Supplement: Supplementary file 13 — Source Data [file 41467_2025_60542_MOESM13_ESM.zip › Source_data/Figure_1/I_top/I_top.pdf]

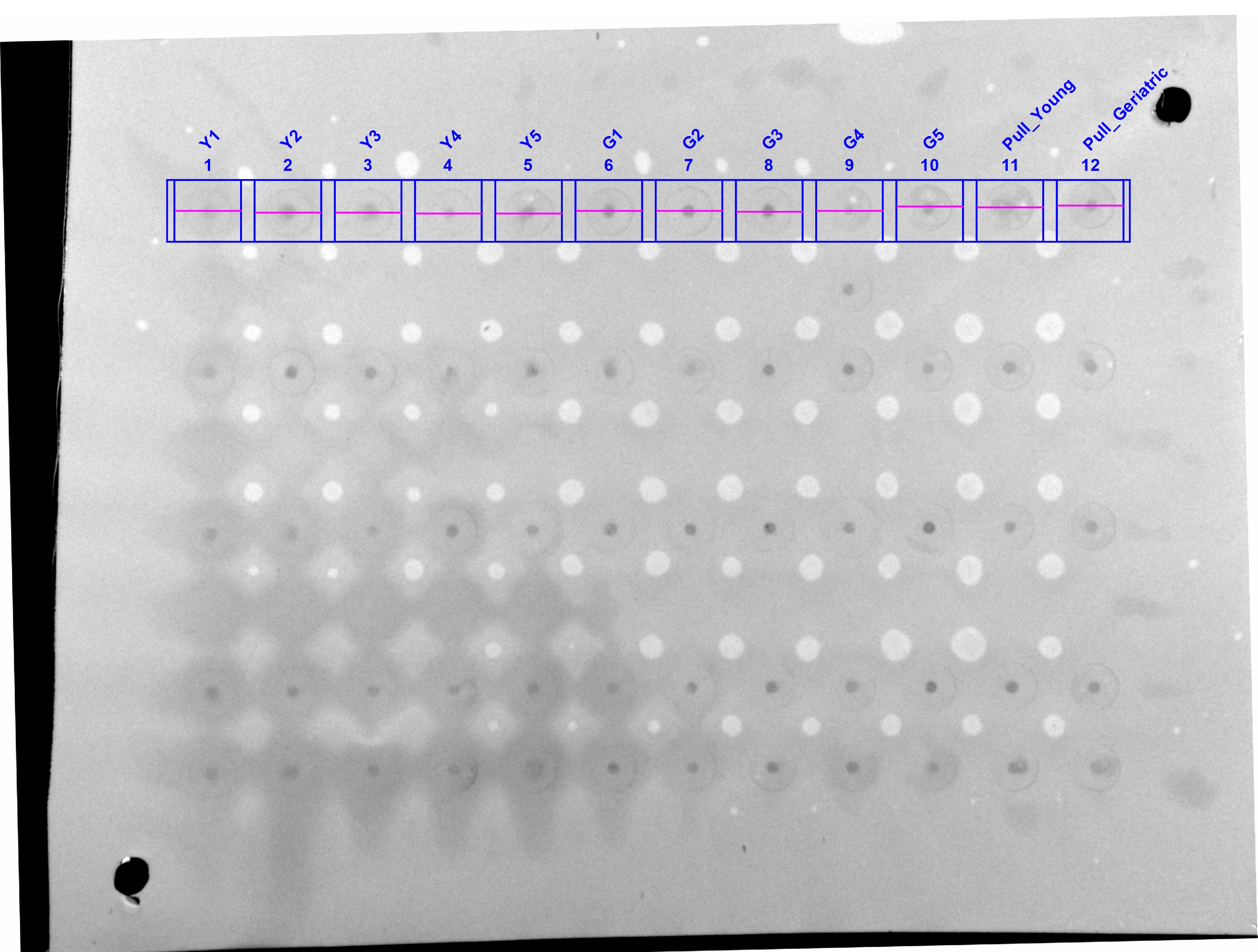

Supplement: Supplementary file 13 — Source Data [file 41467_2025_60542_MOESM13_ESM.zip › Source_data/Figure_S2/C/Ponceau_DotsImage.jpg]

Nrxn1\_K1491\_Mm (Ub)

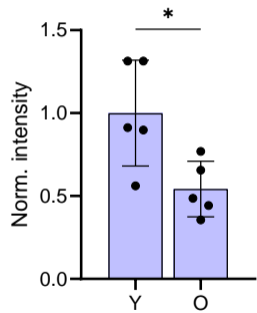

Nrxn1\_Protein\_Mm

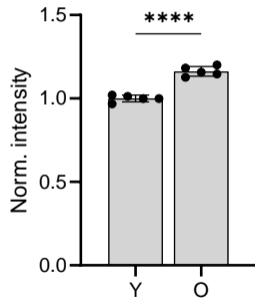

Nrxn1\_K1491\_Kf (Ub)

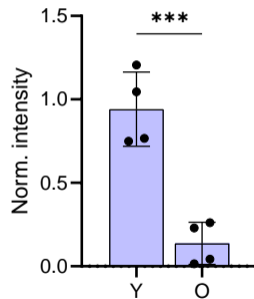

Nrxn1\_Protein\_Kf

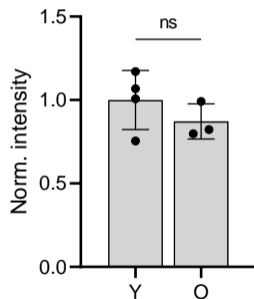

Supplement: Supplementary file 13 — Source Data [file 41467_2025_60542_MOESM13_ESM.zip › Source_data/Figure_2/F/F.pdf]

USP33\_K34 (Ub)

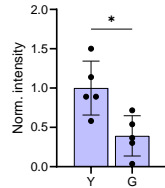

USP33\_Protein

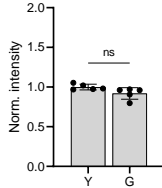

CAMK2A\_K461 (Ub)

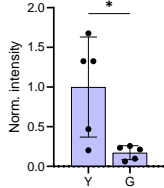

CAMK2A\_Protein

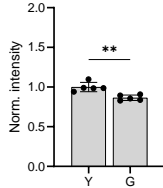

H2AFY\_K189 (Ub)

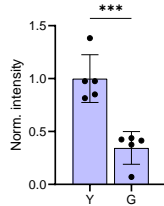

H2AFY\_Protein

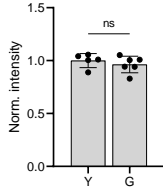

Supplement: Supplementary file 13 — Source Data [file 41467_2025_60542_MOESM13_ESM.zip › Source_data/Figure_1/H/H.pdf]

Myo1D\_K366\_Mm (Ub)

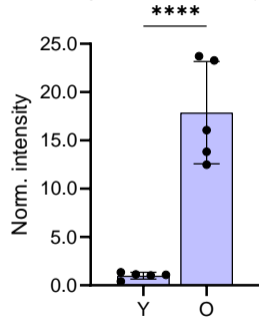

Myo1D\_Protein

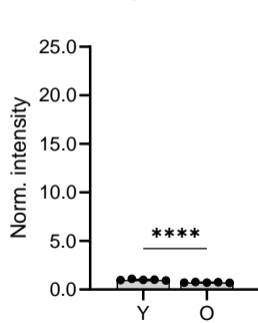

Myo1D\_K366\_Nf (Ub)

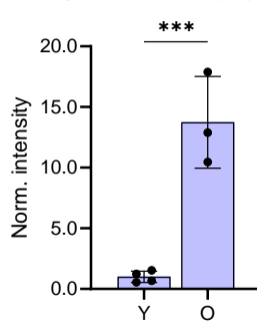

Myo1D\_Nf\_Protein

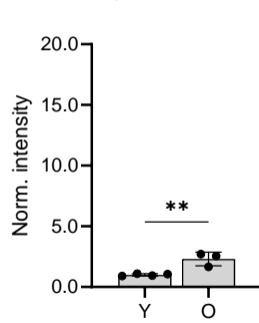

Supplement: Supplementary file 13 — Source Data [file 41467_2025_60542_MOESM13_ESM.zip › Source_data/Figure_2/D/D.pdf]

Gfap\_K336 (Ac)

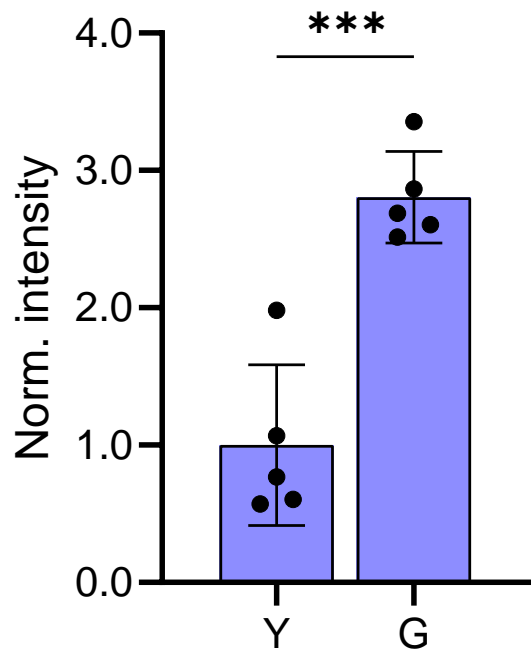

Gfap\_Protein

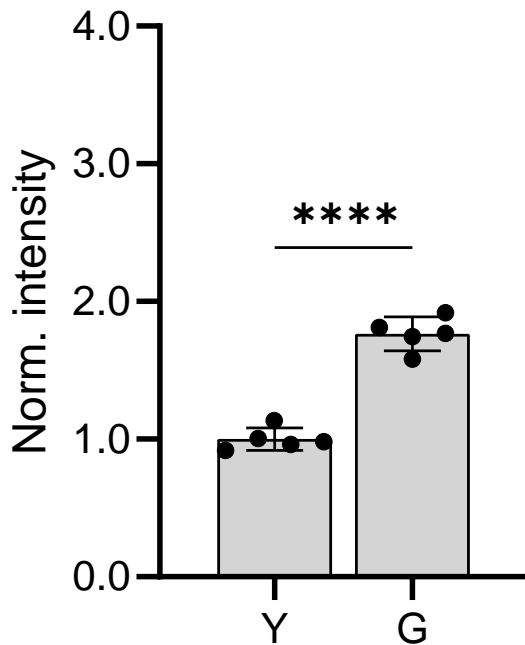

Supplement: Supplementary file 13 — Source Data [file 41467_2025_60542_MOESM13_ESM.zip › Source_data/Figure_S1/H/H.pdf]

# Ubiquitylation enrichment - OvsY

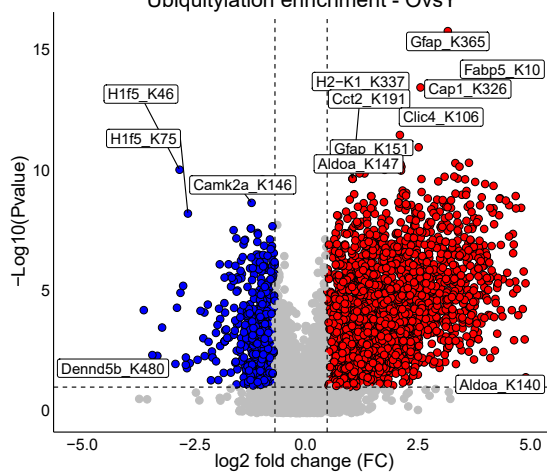

Supplement: Supplementary file 13 — Source Data [file 41467_2025_60542_MOESM13_ESM.zip › Source_data/Figure_1/D/D.pdf]

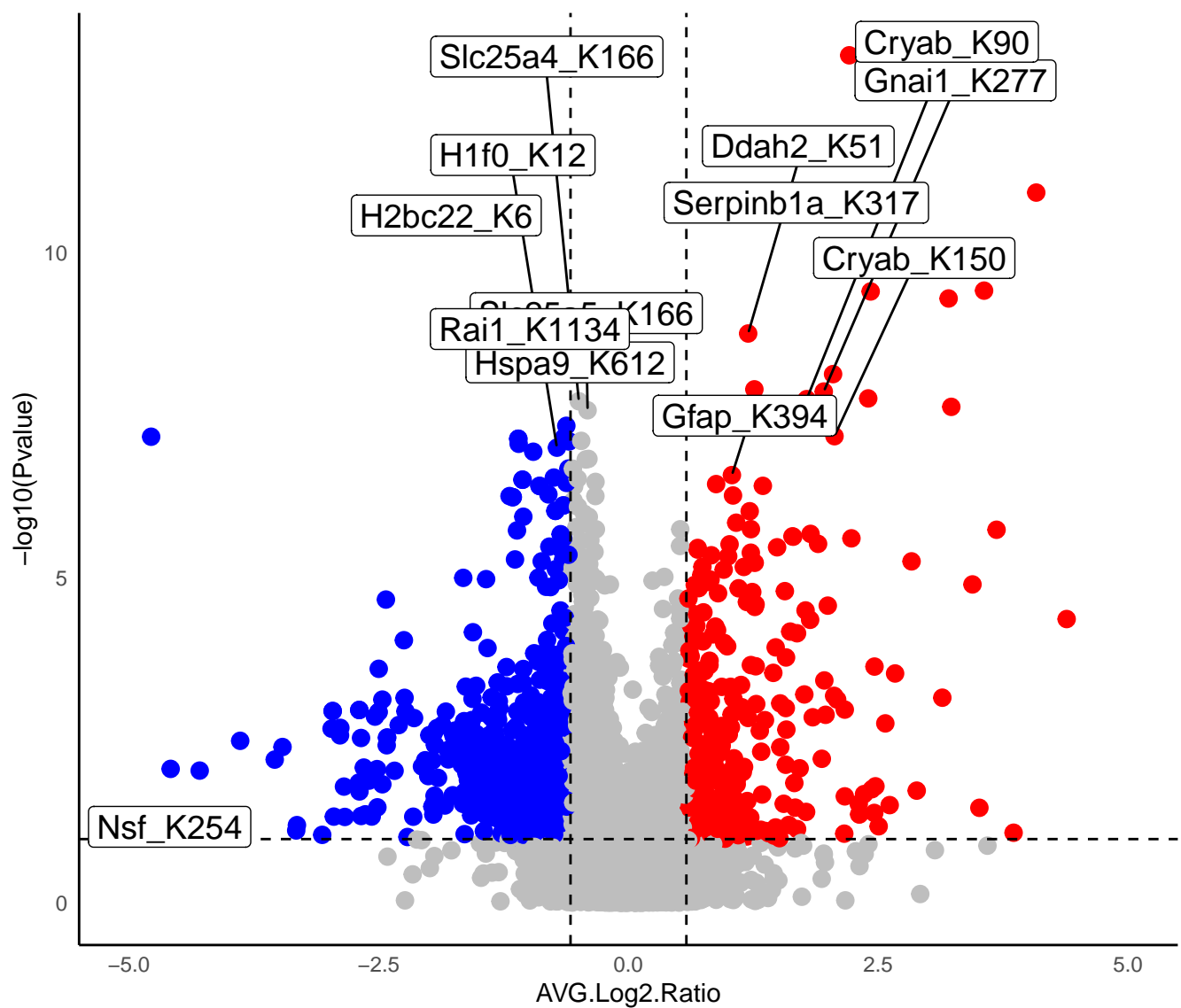

Supplement: Supplementary file 13 — Source Data [file 41467_2025_60542_MOESM13_ESM.zip › Source_data/Figure_S1/F/F.pdf]

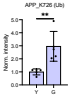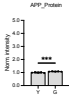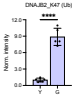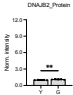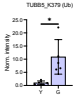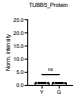

Supplement: Supplementary file 13 — Source Data [file 41467_2025_60542_MOESM13_ESM.zip › Source_data/Figure_1/G/G.pdf]

HSPA8\_K539\_Mm (Ub)

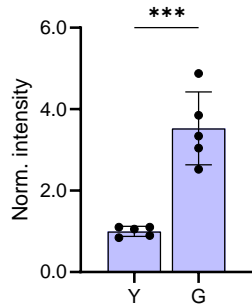

HSPA8\_Protein\_Mm

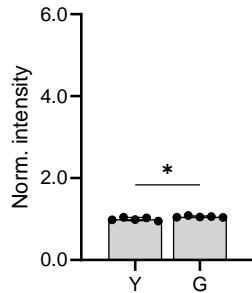

HSPA8\_K539\_Nf (Ub)

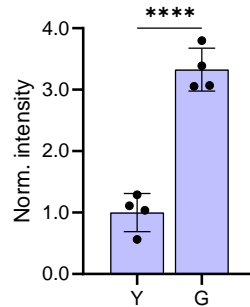

HSPA8\_Protein\_Nf

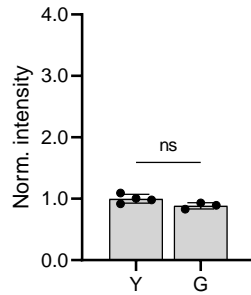

Supplement: Supplementary file 13 — Source Data [file 41467_2025_60542_MOESM13_ESM.zip › Source_data/Figure_2/E/E.pdf]

# Ub changes and Half-life Changes [Aging]

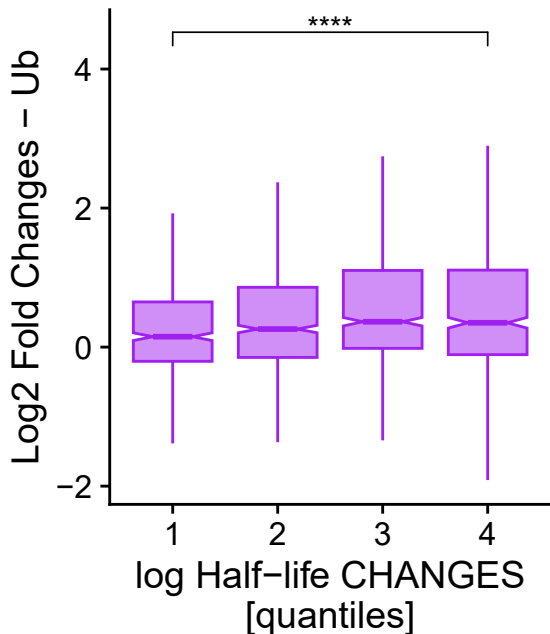

Supplement: Supplementary file 13 — Source Data [file 41467_2025_60542_MOESM13_ESM.zip › Source_data/Figure_1/I_bottom/I_bottom.pdf]

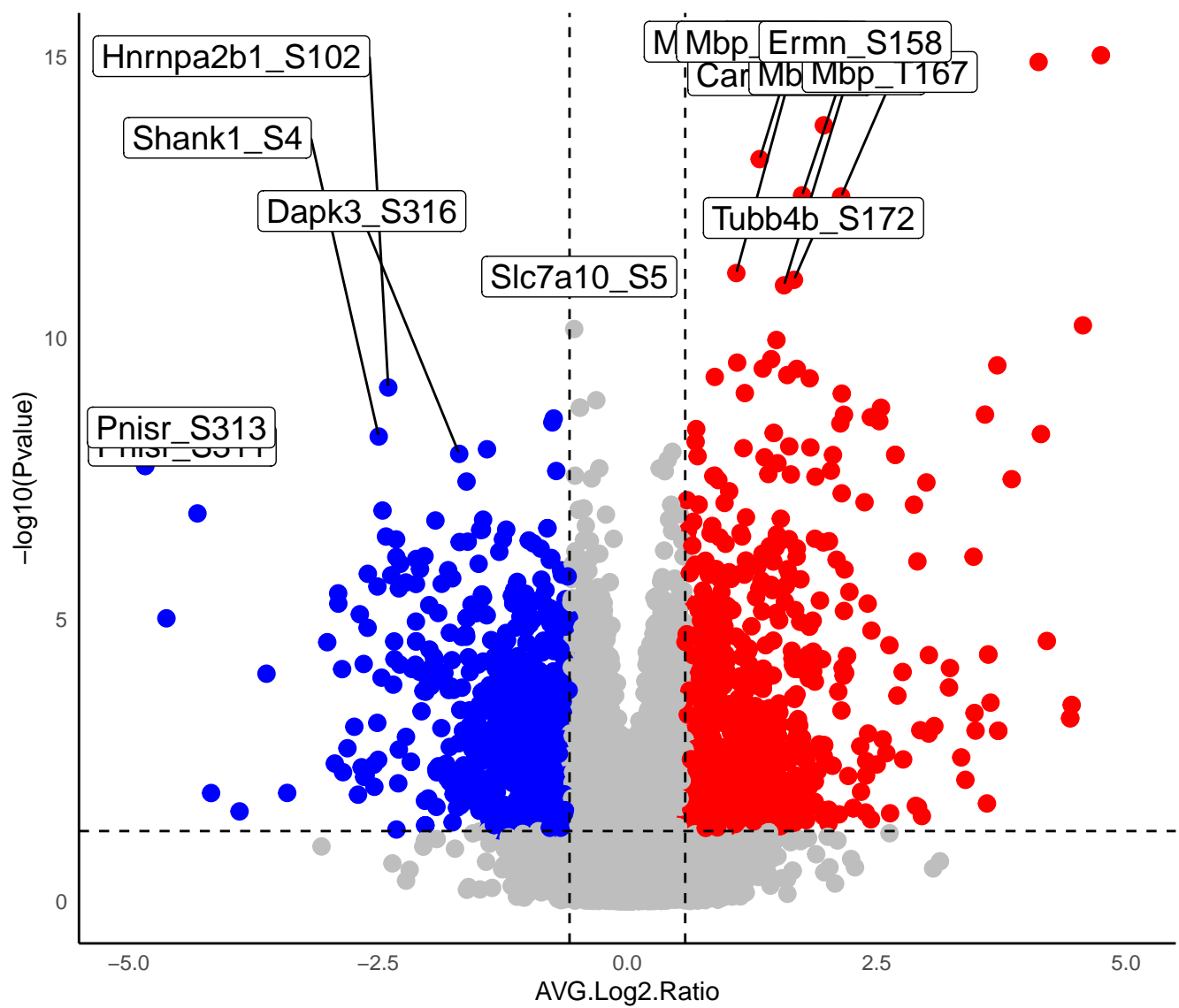

Supplement: Supplementary file 13 — Source Data [file 41467_2025_60542_MOESM13_ESM.zip › Source_data/Figure_S1/C/C.pdf]

Camk2a\_K291\_Mm (Ub)

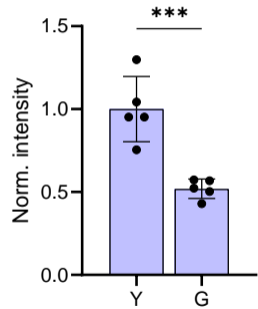

Camk2a\_Protein\_Mm

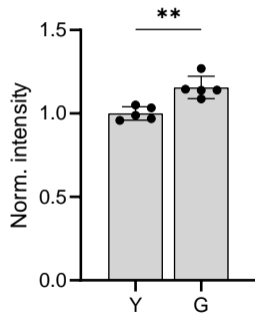

Camk2a\_K291\_Kf (Ub)

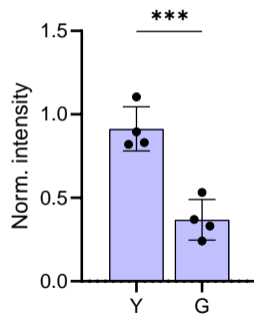

Camk2a\_Protein\_Kf

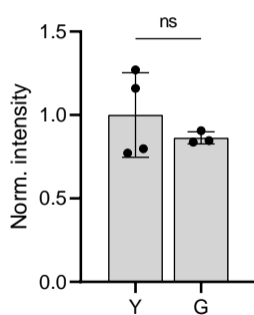

Supplement: Supplementary file 13 — Source Data [file 41467_2025_60542_MOESM13_ESM.zip › Source_data/Figure_2/G/G.pdf]

M.musculus|N.furzeri

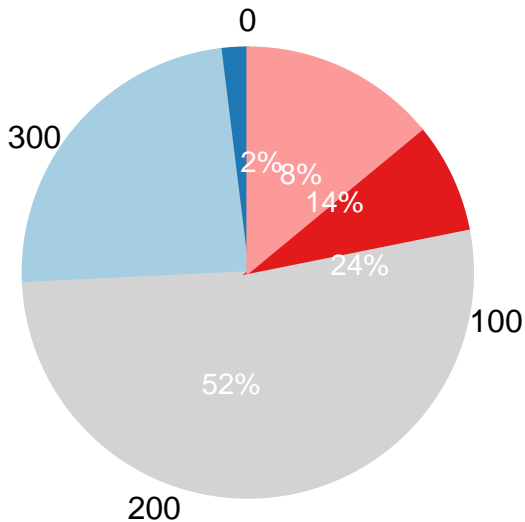

cons

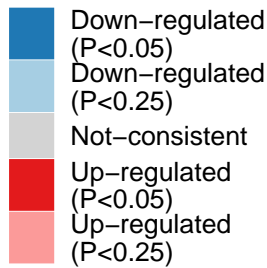

Supplement: Supplementary file 13 — Source Data [file 41467_2025_60542_MOESM13_ESM.zip › Source_data/Figure_2/A/A.pdf]

data ■ Proteome ■ Ubiquitin

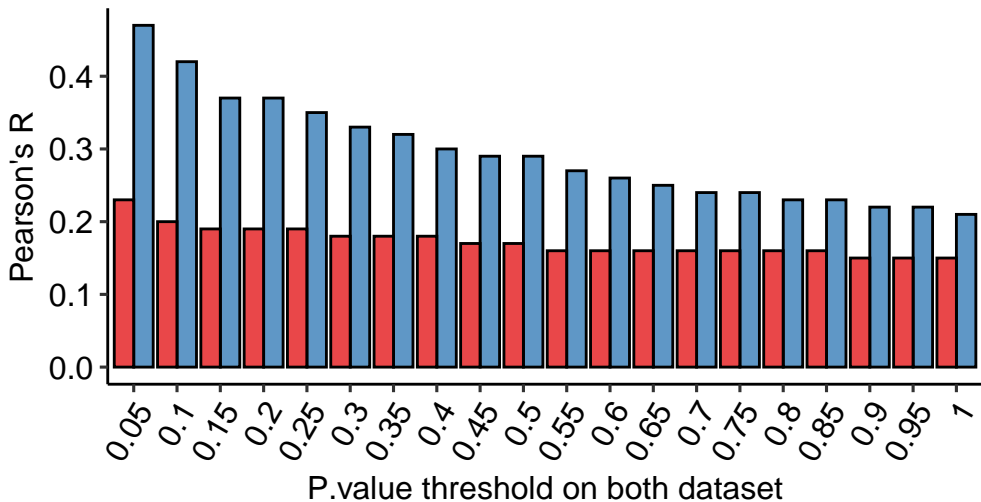

Supplement: Supplementary file 13 — Source Data [file 41467_2025_60542_MOESM13_ESM.zip › Source_data/Figure_2/B/B.pdf]

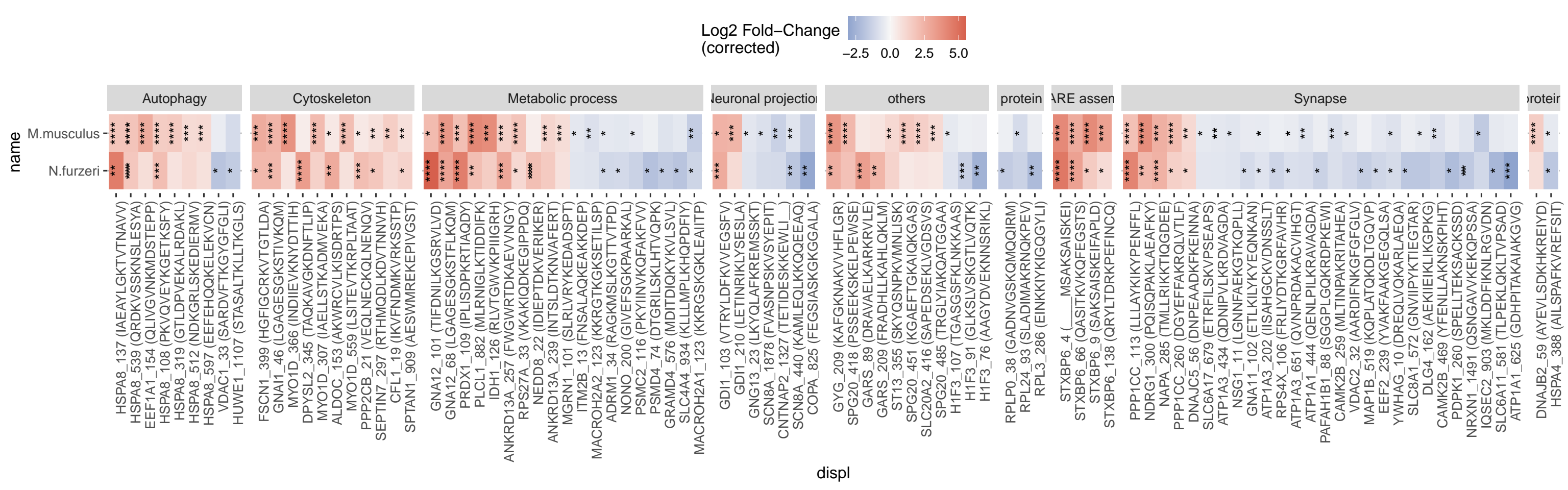

Supplement: Supplementary file 13 — Source Data [file 41467_2025_60542_MOESM13_ESM.zip › Source_data/Figure_2/C/C.pdf]
