# Supplementary material for: Aging and diet alter the protein ubiquitylation landscape in the mouse brain
Source: Nat Commun. 2025 Jun 6;16:5266. doi: 10.1038/s41467-025-60542-6 (PMC12144301; doi:10.1038/s41467-025-60542-6)
Supplement: Supplementary file 13 — Source Data [file 41467_2025_60542_MOESM13_ESM.zip › Source_data/Figure_S7/Figure_S7.pdf]

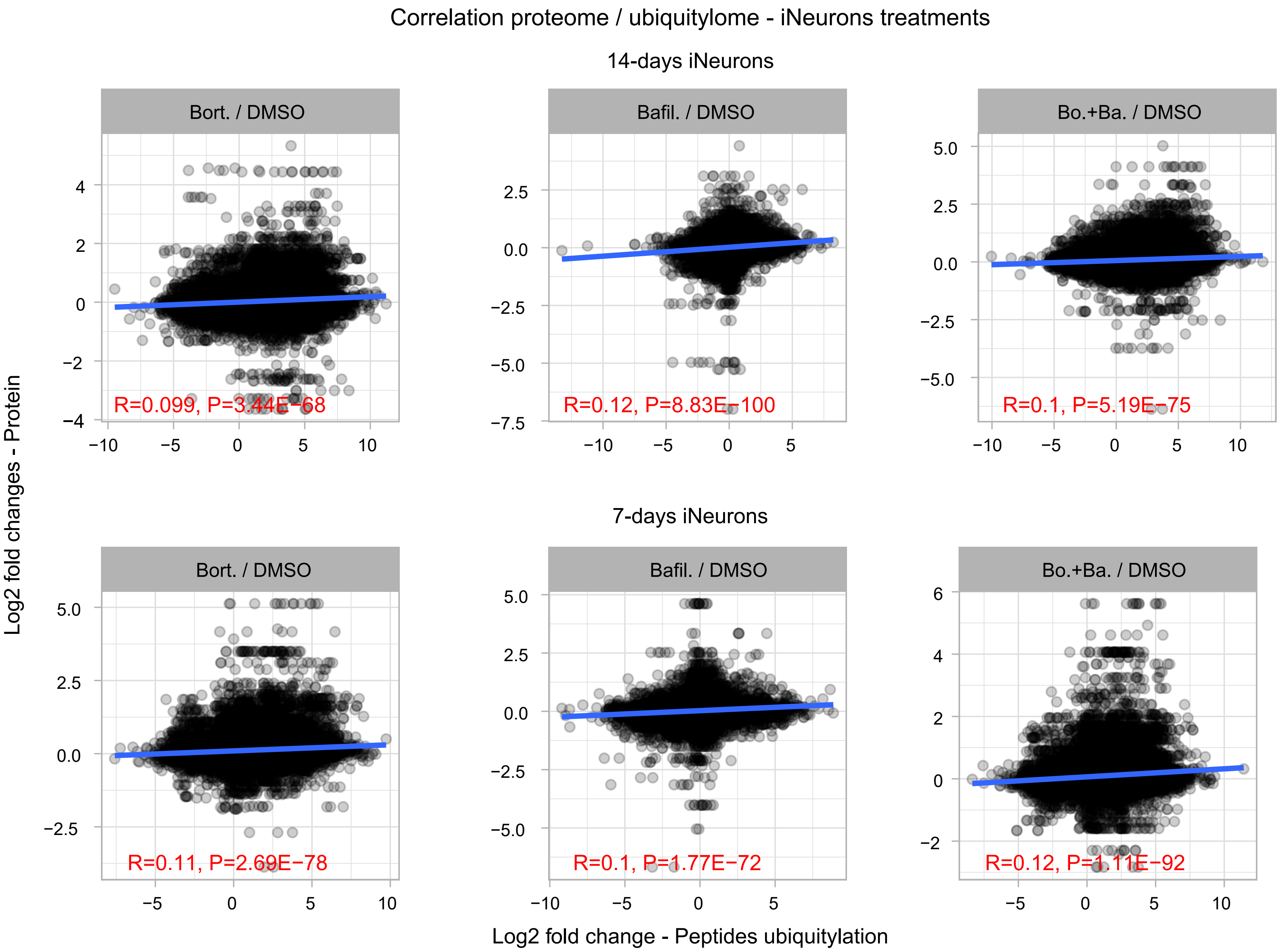

Correlation proteome / ubiquitylome - iNeurons treatments
14-days iNeurons
Bort. / DMSO
R=0.099, P=3.44E−68
Bafil. / DMSO
R=0.12, P=8.83E−100
Bo.+Ba. / DMSO
R=0.1, P=5.19E−75
7-days iNeurons
Bort. / DMSO
R=0.11, P=2.69E−78
Bafil. / DMSO
R=0.1, P=1.77E−72
Bo.+Ba. / DMSO
R=0.12, P=1.11E−92
Log2 fold changes - Protein
Log2 fold change - Peptides ubiquitylation
